# Palladium-Catalyzed Synthesis of Cyclopropylthiophenes and Their Derivatization

**DOI:** 10.3390/molecules28093770

**Published:** 2023-04-27

**Authors:** Tomas Paškevičius, Ringailė Lapinskaitė, Sigitas Stončius, Rita Sadzevičienė, Asta Judžentienė, Linas Labanauskas

**Affiliations:** Department of Organic Chemistry, Center for Physical Sciences and Technology (FTMC), Akademijos g. 7, LT-08412 Vilnius, Lithuania; ringaile.lapinskaite@ftmc.lt (R.L.); sigitas.stoncius@ftmc.lt (S.S.); rita.sadzeviciene@ftmc.lt (R.S.); asta.judzentiene@ftmc.lt (A.J.)

**Keywords:** thiophene, cyclopropylthiophene, Suzuki–Miyaura cross coupling, catalysis

## Abstract

The cyclopropylthiophene moiety has attracted the attention of the scientific community for its potential pharmaceutical applications. However, synthesis of the compounds containing this framework remains challenging, has rarely been reported and remains unresolved. Here we provide optimized syntheses for cyclopropylthiophenes and their derivatives, containing carbonyl, acetyl, carboxylic acid, methyl carboxylate, nitrile, bromide and sulfonyl chloride moieties.

## 1. Introduction

Derivatives of thiophene have been widely used in material sciences and agrochemistry, and have been successfully applied as pharmaceuticals with various biological activities—anticancer, antioxidant, anti-inflammatory, antimicrobial, etc. [1,2,3]. The cyclopropyl moiety with its specific reactivity characteristics has also attracted the attention of the scientific community because of its potential to improve pharmaceutical properties [4,5]. In new combinations it may lead to new biologically active compounds [6]. However, access to cyclopropylthiophene derivatives is limited due to their reactivity [7] and lack of general procedures for their synthesis.

**Scheme 1:**
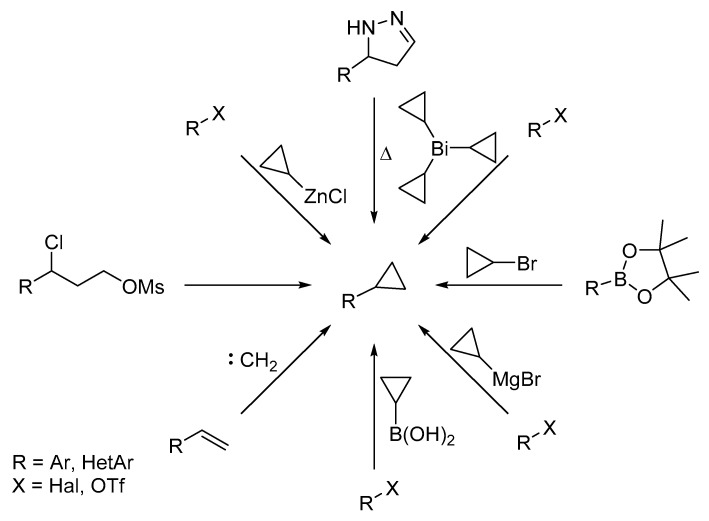
Selected synthesis methods for monosubstituted cyclopropane derivatives.

There are a few methods reported for the synthesis of monosubstituted cyclopropylarenes (Scheme 1) [8,9,10,11,12,13,14,15]. Variations of classical name reactions (Simmons–Smith, Corey–Chaykovsky, Wolff–Kishner) as well as more recent methods, such as various cross-coupling or organometallic reactions, have been successfully used for small scale synthesis. However, these reactions tend to require either harsh conditions (high temperatures), or fairly expensive and air/moisture sensitive reagents/catalysts to achieve good yields. Thiophenes are less stable and more reactive than benzene derivatives; therefore, some of the synthetic methods shown in Scheme 1 cannot be directly applied to the former. Thiophene’s reactivity usually leads to the formation of large amounts of side products and/or decomposition of the material. For example, thermal degradation of various arylpyrazolines yields 70–90% of the desired product [8]. In the case of 5-(thiophen-2-yl)-pyrazoline, the yield of 2-cyclopropylthiophene is a modest 26%, at best [16]. 

**Scheme 2:**
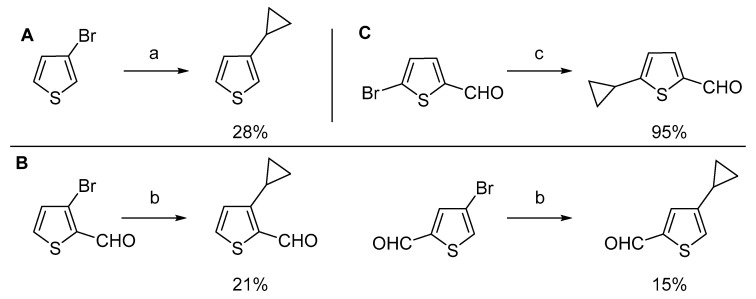
Synthesis of cyclopropylthiophenes. (**A**) Synthesis of of 3-cyclopropylthiophene; (**B**) Synthesis of 3- and 4-cyclopropylthiophenecarbaldehydes; (**C**) Synthesis of 5-cyclopropylthiophene-2-carbaldehyde. Conditions: (a) *c*-PrB(OH)_2_, Pd(OAc)_2_, PCy_3_, K_3_PO_4_, PhMe, H_2_O, 100 °C, 3 d; (b) *c*-PrBKF_3_, Pd(dppf)Cl_2_, K_3_PO_4_, PhMe, H_2_O, 100 °C, 10 h; (c) *c*-PrB(OH)_2_, Pd(OAc)_2_, cataCXium A, Cs_2_CO_3_, PhMe, H_2_O, 100 °C, 16 h.

The aim of this research was to develop general and scalable (0.2–1.5 mol) procedures for the synthesis of a variety of cyclopropylthiophene derivatives from commercially available and inexpensive starting materials. Literature overview and evaluation led to the conclusion that the Suzuki–Miyaura cross-coupling reaction might be the best choice. In general, palladium-catalyzed cross-coupling reactions tend to require mild conditions and work with a variety of catalysts. However, in stark contrast to benzene derivatives, the application of the Suzuki–Miyaura cross-coupling reaction for the synthesis of functionalized cyclopropylthiophenes remains underexplored [6,17,18,19]. In addition, despite high catalyst loading and/or extended reaction times, some of the reported Suzuki–Miyaura reactions of bromothiophenes using standard ligands and Pd sources are rather low-yielding (Scheme 2).

For example, the reaction of 3-bromothiophene using a combination of palladium (II) acetate (5 mol%) and tricyclohexylphosphine (10 mol%) yielded only 28% of 3-cyclopropylthiophene (Scheme 2A) [6]. Suzuki–Miyaura cross-coupling of 3- and 4-bromothiophenecarbaldehydes with potassium cyclopropyltrifluoroborate (Scheme 2B) catalyzed by [1,1′-bis(diphenylphosphino)ferrocene]dichloropalladium(II) (Pd(dppf)Cl_2_, 2 mol%,) furnished corresponding cyclopropylthiophenecarbaldehydes only in 21% and 15% yields, respectively [17]. On the other hand, a combination of palladium (II) acetate (6 mol%) with bulkier and more electron-rich ligand cataCXium A (10 mol%) gave a 95% yield of 5-cyclopropylthiophene-2-carbaldehyde (Scheme 2C) [18]. Herein, we report the application of the Suzuki–Miyaura cross-coupling reaction for the synthesis of functionalized cyclopropylthiophene derivatives and their further derivatization.

## 2. Results and Discussion

**Scheme 3:**
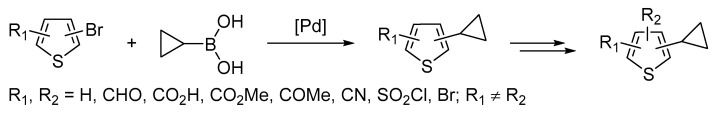
General approach to the synthesis of cyclopropylthiophene derivatives.

During our custom synthesis projects related to the production of various building blocks containing cyclopropylbenzene, amino- and bromocyclopropylpyridine [20] moieties, we successfully applied a catalytic system developed by Buchwald [21]. The palladium (II) acetate and dicyclohexyl(2′,6′-dimethoxy [1,1′-biphenyl]-2-yl)phosphane (SPhos) ligand’s system allows the use of small catalyst loading for the Suzuki–Miyaura cross-coupling reactions. High conversion and good yields can be achieved with this combination using 0.5–5 mol% catalyst loading instead of the more typical 5–10 mol%. We aimed to optimize this reaction for cyclopropanation of various bromothiophenes and, thereafter, to use these molecules for the synthesis of desired building blocks containing various fragments indicated in Scheme 3.

**Scheme 4:**
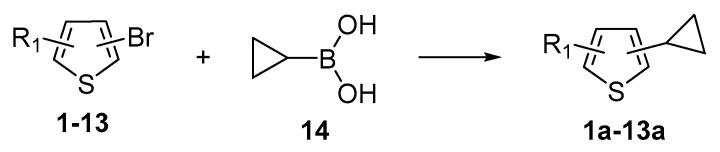
The Suzuki–Miyaura cross-coupling of bromothiophenes. Conditions: Pd(OAc)_2_, SPhos, K_3_PO_4_, PhMe, H_2_O, 90 °C, 2 h.

Thus, large scale Suzuki–Miyaura coupling of parent 2- and 3-bromothiophenes **1** and **2** with 1.3 eq. of cyclopropylboronic acid (**14**) in the presence of K_3_PO_4_ (2 eq.), Pd(OAc)_2_ (1 mol%) and Sphos (2 mol%) furnished the corresponding cyclopropyl derivatives **1a** and **2a** in good yields (Scheme 4, Table 1, entry 1, 2). The scope of the Suzuki–Miyaura coupling reaction was then explored using a range of 2,3-, 2,4-, 2,5- and 3,4-disubstituted bromothiophenes (**3**–**13**, procedure is provided in Supplementary Materials, GP1). High yields of the corresponding cyclopropyl derivatives **3a**–**13a**, bearing ester, methyl ketone and aldehyde moieties, were attained on 0.3–1.5 mol scale (Table 1, entries 3–13). The conversion of starting materials, as well as product purity, was evaluated using GC-MS. In all cases, starting material was consumed after 2 h of heating at 90 °C. In the case of compounds **1**, **2** and esters **3**–**6** (Table 1, entries 1–6), 1 mol% of the Pd precatalyst and 2 mol% of the ligand were used. It was noticed that larger scale reactions with 1 mol% catalyst loading were highly exothermic, leading to the boiling of the reaction mixtures even without an external source of heating. Although the Suzuki–Miyaura reaction of bromoesters was still feasible with 0.5 mol% of Pd(OAc)_2_, e.g., derivative **7** (Table 1, entry 7), lower catalyst loadings led to incomplete conversions and/or higher amounts of by-products. On the other hand, less fastidious coupling partners, such as bromothiophenes bearing methyl ketone (**8**, Table 1, entry 8) and particularly aldehyde (**9**–**13**, Table 1, entries 9–13) functional groups, reacted uneventfully using just 0.5 and 0.25 mol% of Pd precatalyst, respectively.

The products **1a**–**13a** were isolated using filtration through a Celite^®^, liquid-liquid extraction, followed by filtration through a plug of silica gel and distillation in 77–93% yields. Only in one instance was additional recrystallization necessary to reach a purity above 98%. The purification of methyl 2-cyclopropylthiophene-3-carboxylate (**7a**, Table 1, entry 7) by distillation yielded a product of only 94% purity. Following the crystallization from hexanes, compound **7a** was obtained in a 69% yield and exceeded 99% purity.

**Scheme 5:**
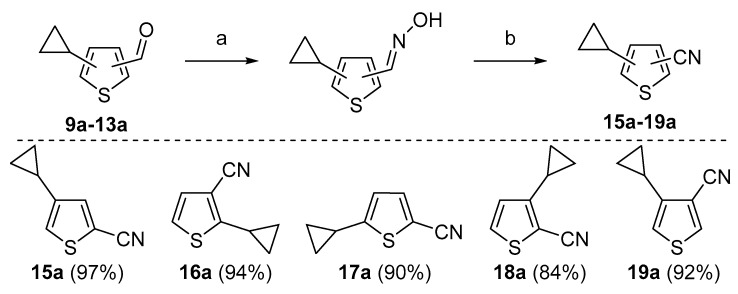
Conversion of cyclopropylthiophene aldehydes (**9a**–**13a**) to the corresponding nitriles (**15a**–**19a**). Conditions: (**a**) NH_2_OH·HCl, Py, 90 °C; (**b**) Ac_2_O, Py, 80 °C.

Cyclopropylthiophenecarbonitriles were envisioned as an additional set of targeted molecules. However, due to the scarcity of bromothiophenecarbonitriles as starting materials at the beginning of this project, it was decided to access this group of compounds by converting Suzuki–Miyaura cross-coupling products. After evaluating common procedures for the nitrile synthesis that might be applicable to access thiophenecarbonitriles, the most environmentally friendly, economical and safe path was chosen for the aldehydes **9a**–**13a** (Scheme 5, procedure is provided in Supplementary Materials, GP2). The one pot procedure consisted of two steps—the conversion of aldehydes to oximes and their subsequent dehydration to the nitriles with acetic anhydride. This allowed us to avoid additional isolation and purification of oxime intermediates. After all the starting aldehyde was consumed, dropwise addition of acetic anhydride to a hot reaction mixture furnished the desired nitriles **15a**–**19a** in good to excellent yields after liquid-liquid extraction and vacuum distillation (84–97%).

**Scheme 6:**
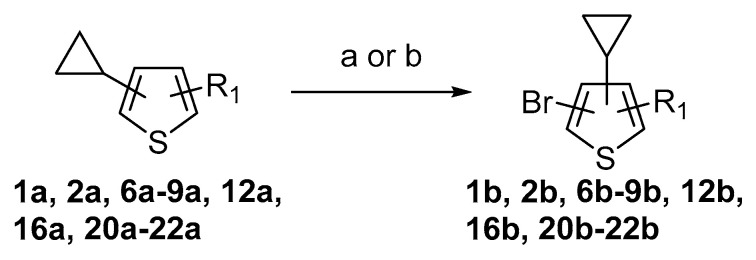
Synthesis of bromocyclopropylthiophenes **1b**, **2b**, **6b**–**9b**, **12b**, **16b**, **20b**–**22b**. Conditions: (**a**) NBS, DCM, AcOH; (**b**) Br_2_, NaOAc, AcOH.

A set of the cyclopropylthiophenes was submitted to the bromination reaction to increase the variety and functionality of the di- and tri-substituted building blocks (Scheme 6, Table 2). Cyclopropylthiophenes **1a** and **2a** were successfully brominated using *N*-bromosuccinimide (NBS) as a mild brominating agent (procedure is provided in Supplementary Materials, GP4). Desired products **1b** and **2b** (Table 2, entries 1–2) were isolated in 72% and 94% yields, respectively. With careful control of NBS addition and reaction temperature (15 °C), only traces of overbrominated products were detected. However, these conditions proved to be too mild for the bromination of disubstituted thiophenes containing aldehyde, ketone, nitrile, ester or carboxylic acid functionalities. In most cases, even after extended reaction times, only a small amount of product was detected (GC-MS). We found that elemental bromine (Br_2_) can be used for more complex substrates. Because of the cyclopropyl moiety’s sensitivity to acids the reactions had to be performed in buffered solutions (NaOAc/AcOH). The products were isolated in 74–96% yields through liquid-liquid extraction and crystallization.

In most cases, excellent yields of bromothiophenes **6b**–**9b** and **16b**, bearing ester (Table 2, entries 3–4), ketone (entry 5), aldehyde (entry 6) and nitrile (entry 8) functionalities, were attained (procedure is provided in Supplementary Materials, GP5). Alternatively, bromonitriles, such as **15b** (Table 2, entry 9), can be obtained from the corresponding bromoaldehyde via oxime dehydration as described above (Scheme 5). Carboxylic acids **20a**–**22a** were synthesized via saponification (1M aq. NaOH) of the corresponding esters **3a**, **5a and 6a** in 98–99% yields (procedure is provided in Supplementary Materials, GP3). The bromination of acids **20a** and **22a** proceeded well and yielded products in 84% and 96% yields, respectively (Table 2, entries 10 and 12). Under the same conditions 3-cyclopropylthiophene-2-carboxylic acid (**21a**, Table 2, entry 11) provided mainly a mixture of decarboxylative bromination products instead of the desired compound **21b**. Similar unsatisfactory results were obtained in the bromination of 3-cyclopropylthiophene-2-carbaldehyde (**12a**, Table 2**,** entry 7).

**Scheme 7:**
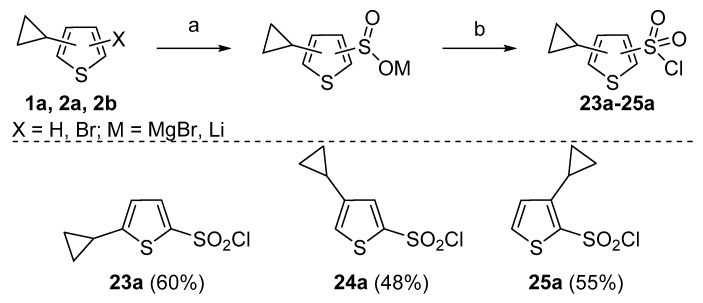
Synthesis of cyclopropylthiophenesulfonyl chlorides **23a**–**25a**. Conditions: (**a**) 1. Mg or *n*-BuLi; 2. SO_2_; (**b**) NCS.

Last, we aimed to prepare a set of cyclopropylthiophenesulfonyl chlorides **23a**–**25a** (Scheme 7). Chlorosulfonic acid (ClSO_3_H) has been successfully used for the synthesis of various *para*-substituted arylsulfonylchlorides [22,23]. However, the reagent’s substantial oxidative properties limit its application for the synthesis of cyclopropylthiophene derivatives. On the other hand, formation of the organometallic derivatives of the corresponding bromocyclopropylthiophenes is a more versatile way to access cyclopropylthiophenesulfonyl chlorides [24]. Organolithium and organomagnesium compounds react with sulfur dioxide to form sulfinic acid salts. Furthermore, they can be oxidatively chlorinated with sulfuryl chloride to provide sulfonyl chlorides. It was found that when this transformation was attempted with organomagnesium intermediate, reactions provided inseparable mixtures of corresponding sulfonyl chlorides and bromides. This might have been caused by the oxidative nature of sulfuryl chloride towards bromide ions and the presence of magnesium. The use of *N*-chlorosuccinimide (NCS) instead of sulfuryl chloride was able to partially suppress formation of sulfonyl bromides in the reaction mixture. However, this side reaction was completely eliminated when *n*-butyl lithium (*n*-BuLi) was used. The direct lithiation of compounds **1a** and **2a** and subsequent treatment with SO_2_ and NCS yielded the desired products **23a** and **24a** in adequate yields (60 and 48%, respectively) (procedure is provided in Supplementary Materials, GP6). Analogously, sulfonyl chloride **25a** was synthesized from 2-bromo-3-cyclopropylthiophene (**2b**) via bromine-lithium exchange (55%). It is worth mentioning that in the reaction of **2b,** the formation of compound **24a** was detected (6%). Sulfonyl chlorides **23a**–**25a** were isolated by filtration through a layer of Celite^®^ and crystallization from hexanes.

## 3. Materials and Methods

### 3.1. General Information

All reactions were performed under an argon atmosphere unless otherwise stated. Commercially available starting materials were generously provided by Apollo Scientific and used without further purification. Tetrahydrofuran (THF) was freshly distilled from Na/benzophenone. Toluene for the Suzuki–Miyaura cross-coupling reactions was redistilled prior to use. Some of the compounds described in this article are known and have been previously reported; for compounds **1a**, **2a**, **11a**, **12a**, **1b**, **2b**, see reference [7], for compound **6a**—[25], **8a**—[9] and **9a**—[17].

NMR spectra were recorded on a Bruker Avance III spectrometer (400 MHz for ^1^H NMR and 100 MHz for ^13^C NMR). Chemical shifts δ are reported with respect to the residual solvent peak and are given in ppm. GC-MS analyses were performed on a Shimadzu GCMS-QP2010 ultra plus system using a Restek Rxi-5 ms column (30 m, 0.25 mmID). Reactions were monitored by TLC using Merk TLC silica gel 60 F_254_ plates and visualized using UV light (254 nm) or KMnO_4_ stain. Column chromatography was performed on ZEOprep 60 silica gel (35–70 µm, Apollo Scientific). Melting points were measured in open capillaries using a Mettler Toledo FP90 central processor equipped with Mettler Toledo FP81HT MBC cell.

### 3.2. Synthesis

Selected general procedures are provided below. All details for the synthesis and characterization of the products are provided in Supplementary Information (SI).

**General procedure for Suzuki**–**Miyaura cross-coupling reaction** (**1a**–**13a**): A solution of bromothiophene (1 eq.) in toluene (2 mL/mmol) was added to a three-neck round bottom flask equipped with mechanical stirring and a reflux condenser. It was followed by addition of cyclopropylboronic acid (1.3 eq.) and anhydrous potassium phosphate (2 eq.). After degassing the reaction mixture with a flow of argon through the solution for 30 min, palladium (II) acetate (1–0.25 mol%) and SPhos (2–0.5 mol%) (Pd:L 1:2) were added. The reaction mixture was stirred under argon for 10 min and degassed water (0.1 mL/mmol of substrate) was added in one portion. The mixture was then heated to 90 °C and the reaction progress was monitored by GC-MS analysis until the bromothiophene was completely consumed (1–2 h). Then the reaction content was allowed to cool to room temperature and enough water to dissolve all inorganic material was added. The resulting solution was filtered through a layer of Celite^®^ and the phases were separated. The organic phase was washed with 0.5 M HCl (aq), and water, then dried with sodium sulfate, and filtered through a layer of silica gel and concentrated in vacuo. The product was purified by vacuum distillation.

**General procedure for the synthesis of cyclopropylthiophenecarbonitriles 15a**–**19a**, **15b**: Hydroxylamine hydrochloride (1.4 eq.) was added in a single portion to a solution of cyclopropylthiophenecarboxaldehyde in pyridine (0.8 mL/mmol). The resulting mixture was heated at 90 °C for 1h. Then it was cooled down to 60 °C and acetic anhydride (10 eq.) was added in a dropwise manner (caution: highly exothermic reaction!). After the addition was completed, the reaction mixture was heated at 90 °C for 2 h, then cooled to room temperature and poured onto ice and acidified to pH = 5 with 6 M HCl. The product was extracted with hexanes, combined organic phases were washed with water, and with saturated sodium bicarbonate solution, then dried with sodium sulfate, filtered, and concentrated in vacuo. The product was purified by vacuum distillation or crystallization. A small scale sample of compound **18a** was purified by flash chromatography.

**General procedure for the bromination of cyclopropylthiophenes 6a**–**9a**, **16a**, **20a**, **22a:** Sodium acetate trihydrate (1 eq.) was added to a solution of cyclopropylthiophene (1 eq.) in glacial acetic acid (1.43 mL/mmol) at 15 °C. A solution of elemental bromine in glacial acetic acid (1.5 M, 1.05 eq.) was added dropwise maintaining the reaction temperature below 20 °C. After the completed addition of bromine, the reaction mixture was stirred at room temperature for 12 h and then poured into water.

**For compounds 6b**–**9b**, **16b:** products were extracted with hexanes, combined organic phases were washed with water, then saturated sodium bicarbonate solution, then dried with sodium sulfate and filtered. After removal of solvents in vacuo the product was purified by crystallization from hexanes.

**For compounds 20b and 22b:** products were collected by filtration and washed with deionized water.

**General procedure for the synthesis of cyclopropylthiophenesulfochlorides 23a**–**25a**: *n*-BuLi (2.5 M in hexanes, 1.1 eq.) was added dropwise to a solution of cyclopropylthiophene (1 eq.) in THF (3 mL/mmol) while maintaining reaction temperature at −78 °C. The reaction mixture was stirred at −78 °C for 40 min to ensure complete lithiation. Liquefied sulfur dioxide (2 eq.) was then added dropwise via cannula to the reaction mixture while keeping the temperature below −60 °C and stirred for an additional 30 min. *N*-chlorosuccinimide (NCS) (1 eq.) was then added in a single portion and the mixture was allowed to warm up to room temperature. Solvents were removed on a rotary evaporator. The remaining residue was suspended in hexanes, filtered through a layer of Celite^®^ and concentrated in vacuo. The product was purified by recrystallization from a minimal amount of hexanes at 0 °C.

## 4. Conclusions

Through this study we were able to optimize general procedures for an easily scalable synthesis and derivatization of various cyclopropylthiophenes. The Suzuki–Miyaura cross-coupling reaction using palladium (II) acetate (0.25–1 mol%) and SPhos (0.5–2 mol%) yielded the desired compounds in 69–93% yields. Further derivatization was accomplished to access di- and trisubstituted cyclopropylthiophenes containing nitrile, bromide, and sulfonyl chloride functionalities. Unexpected reactivities were noticed for 3-cyclopropyl-2-substituted thiophenes during the attempted bromination. Compounds **21a** and **12a** underwent decarboxylative/decarbonylative bromination side reactions. All targeted products were successfully purified using techniques fit for larger scale reactions—liquid-liquid extraction, followed by vacuum distillation and/or crystallization.

## Figures and Tables

**Table 1:** The Suzuki–Miyaura coupling of bromothiophenes **1**–**13** with boronic acid **14**.

Entry ^a^	Substrate		Scale, mol	Pd, mol%	Product		Yield, %
1	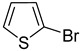	**1**	0.74	1	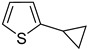	**1a**	77
2	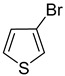	**2**	0.74	1	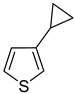	**2a**	78
3	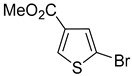	**3**	0.31	1	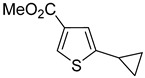	**3a**	89
4	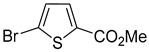	**4**	0.43	1	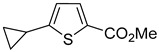	**4a**	87
5	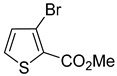	**5**	0.47	1	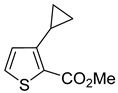	**5a**	84
6	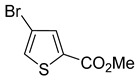	**6**	0.43	1	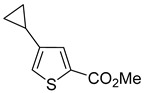	**6a**	88
7	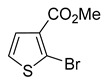	**7**	0.39	0.5	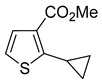	**7a**	86 (69 ^b^)
8	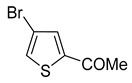	**8**	0.48	0.5	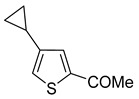	**8a**	93
9	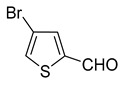	**9**	1.54	0.5	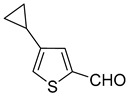	**9a**	89
10	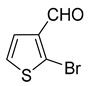	**10**	0.34	0.5	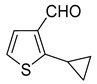	**10a**	86
11	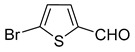	**11**	1.05	0.25	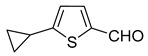	**11a**	88
12	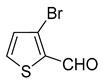	**12**	0.78	0.25	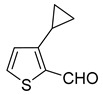	**12a**	87
13	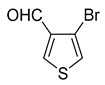	**13**	0.78	0.25	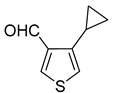	**13a**	80

^a^ Pd(OAc)_2_, SPhos (Pd:L = 1:2), K_3_PO_4_, PhMe, H_2_O, 90 °C, 2 h; ^b^ Yield after additional recrystallization.

**Table 2:** Synthesis of bromocyclopropylthiophenes **1b**, **2b**, **6b**–**9b**, **12b**, **15b**, **16b**, **20b**–**22b**.

Entry	Substrate		Product		Yield, %
1 ^a^	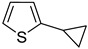	**1a**	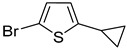	**1b**	72
2 ^a^	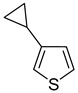	**2a**	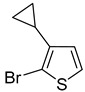	**2b**	94
3 ^b^	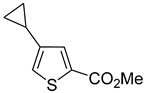	**6a**	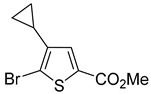	**6b**	74
4 ^b^	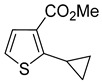	**7a**	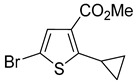	**7b**	74
5 ^b^	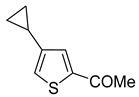	**8a**	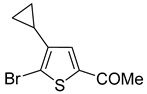	**8b**	87
6 ^b^	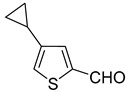	**9a**	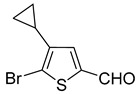	**9b**	80
7 ^b^	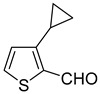	**12a**	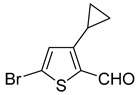	**12b**	n/a
8 ^b^	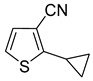	**16a**	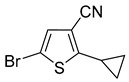	**16b**	91
9 ^c^	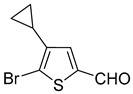	**9b**	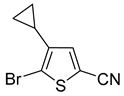	**15b**	90
10 ^b^	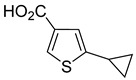	**20a**	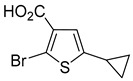	**20b**	84
11 ^b^	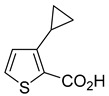	**21a**	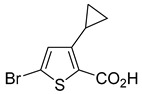	**21b**	n/a
12 ^b^	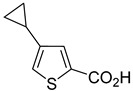	**22a**	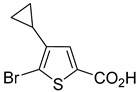	**22b**	96

^a^ NBS, DCM, AcOH, 15 °C; ^b^ Br_2_, NaOAc, AcOH, 10–15 °C; ^c^ (a) NH_2_OH·HCl, Py, 90 °C; (b) Ac_2_O, Py, 80 °C.

## Data Availability

The data for the synthesized compounds presented in this study are available in Supplementary Material.

## Supplementary Materials

The following supporting information can be downloaded at: https://www.mdpi.com/article/10.3390/molecules28093770/s1, compound synthesis details and characterization data for synthesized products.
