# Supplementary material for: Palladium-Catalyzed Synthesis of Cyclopropylthiophenes and Their Derivatization
Source: Molecules. 2023 Apr 27;28(9):3770. doi: 10.3390/molecules28093770 (PMC10180236; doi:10.3390/molecules28093770)
Supplement: Supplementary file 1 [file molecules-28-03770-s001.zip › molecules-2383860-supplementary.pdf]

Palladium-Catalyzed Synthesis of Cyclopropylthiophenes and Their Derivatization

Tomas Paškevičius * , RRingailė Lapinskaitė , Sigitas Stončius, Rita Sadzevičienė, Asta Judžentienė and Linas Labanauskas *

Department of Organic Chemistry, Center for Physical Sciences and Technology (FTMC), Akademijos g. 7, LT-08412 Vilnius, Lithuania; ringaile.lapinskaite@ftmc.lt (R.L.); sigitas.stoncius@ftmc.lt (S.S.); rita.sadzeviciene@ftmc.lt (R.S.); asta.judzentiene@ftmc.lt (A.J.)
* Correspondence: tomas.paskevicius@ftmc.lt (T.P.); linas.labanauskas@ftmc.lt (L.L.)

Supplementary Materials

Contents

1. Materials and Methods 2
1. 1. General information. 2
1. 2. Synthesis. 2
1. 2. 1. GP1. General procedure for Suzuki-Miyaura cross coupling reaction 2
1. 2. 2. GP2. General procedure for the synthesis of cyclopropylthiophenecarbonitriles (**15a**-**19a**, **15b**)..5
1. 2. 3. GP3. General procedure for the hydrolysis of esters (**3a**, **5a**, **6a**) 7
1. 2. 4. GP4. General procedure for the bromination of cyclopropylthiophenes **1a** and **2a** 8
1. 2. 5. GP5. General procedure for the synthesis of cyclopropylthiophenes **6b**-**9b**, **16b**, **20b**, **22b** 9
1. 2. 6. GP6. General procedure for the synthesis of cyclopropylthiophenesulfochlorides **23a**-**25a** 11
2. NMR spectral outtakes 12
3. References 46

1. Materials and Methods

1. 1. General information.

All reactions were performed under an argon atmosphere unless otherwise stated. Commercially available starting materials were generously provided by Apollo Scientific and used without further purification. Tetrahydrofuran (THF) was freshly distilled from Na/benzophenone. Toluene for the Suzuki-Miyaura cross coupling reactions was redistilled prior to use. Compounds **1a**, **2a**, **11a**, **12a**, **1b**, **2b** [1], **6a** [2], **8a** [3] and **9a** [4] are known and have been previously reported.

NMR spectra were recorded on Bruker Avance III spectrometer (400 MHz for ^{1}H NMR and 100 MHz for ^{13}C NMR). Chemical shifts δ are reported with respect to the residual solvent peak and are given in ppm. GC-MS analyzes were performed on Shimadzu GCMS-QP2010 ultra plus system using a Restek Rxi-5ms (30 m, 0.25 mmID) column. Reactions were monitored by TLC using Merk TLC silica gel 60 F_{254} plates and visualized using UV light (254 nm) or $KMnO_4$ stain. Column chromatography was performed on ZEOprep 60 silica gel (35-70 μm, Apollo Scientific). Melting points were measured in open capillaries using Mettler Toledo FP90 central processor equipped with Mettler Toledo FP81HT MBC cell.

1. 2. Synthesis.

1. 2. 1. GP1. General procedure for Suzuki-Miyaura cross coupling reaction

A solution of bromothiophene (1 eq.) in toluene (2 ml/mmol) was added to a three-neck round bottom flask equipped with mechanical stirring and a reflux condenser. It was followed by addition of cyclopropylboronic acid (1.3 eq.) and anhydrous potassium phosphate (2 eq.). After degassing reaction mixture with a flow of argon through the solution for 30 min, palladium (II) acetate (1-0.25 mol%) and SPhos (2-0.5 mol%) (Pd:L 1:2) was added. Reaction mixture was stirred under argon for 10 min and degassed water (0.1 ml/mmol of substrate) was added in one portion. The mixture was then heated to 90 °C and the reaction progress was monitored by GC-MS analysis until the bromothiophene was completely consumed (1-2 h). Then the reaction content was allowed to cool to room temperature and enough water to dissolve all inorganic material was added. The resulting solution was filtered through a layer of Celite® and phases were separated. Organic phase was washed with 0.5 M HCl, water, dried with sodium sulfate, filtered through a layer of silica gel and concentrated *in vacuo*. The product was purified by vacuum distillation.

2-Cyclopropylthiophene (1a)

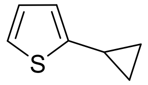

GP1 was performed using 2-bromothiophene (**1**, 120 g, 0.736 mol), palladium acetate (1 mol%), SPhos (2 mol%). After distillation **1a** (69.97 g, 77%) was obtained as a colorless liquid.

(**1a**): bp = 53-55 °C (15 mbar), lit. bp = 58 °C (10 mmHg) [1]; ^{1}H NMR (400 MHz, $CDCl_3$) δ 7.04 (dd, *J* = 5.2, 1.3 Hz, 1H), 6.89 (dd, *J* = 5.2, 3.5 Hz, 1H), 6.77 (dd, *J* = 3.2, 1.1 Hz, 1H), 2.16 – 2.04 (m, 1H), 1.05 – 0.93 (m, 2H), 0.80 – 0.70 (m, 2H); ^{13}C NMR (100 MHz, $CDCl_3$) δ 148.7, 126.8, 122.7, 122.1, 11.1, 9.9; MS (EI) calcd for C_7H_8S [M$^+$]: 124; found 124.

3-Cyclopropylthiophene (2a)

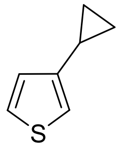

GP1 was performed using 3-bromothiophene (**2**, 120 g, 0.736 mol), palladium acetate (1 mol%), SPhos (2 mol%). After distillation **2a** (71.59 g, 78%) was obtained as a colorless liquid.
(**2a**): bp = 56-58 °C (14 mbar), lit. bp = 58 °C (10 mmHg) [1]; ^{1}H NMR (400 MHz, $CDCl_3$) δ 7.22 (td, *J* = 3.3, 1.5 Hz, 1H), 6.90 – 6.86 (m, 1H), 6.86 – 6.82 (m, 1H), 2.00 – 1.88 (m, 1H), 0.95 – 0.88 (m, 2H), 0.69 – 0.62 (m, 2H); ^{13}C NMR (100 MHz, $CDCl_3$) δ 145.2, 126.3, 125.4, 118.1, 11.4, 8.6; MS (EI) calcd for C_7H_8S [M^+]: 124; found 124.

Methyl 5-cyclopropylthiophene-3-carboxylate (3a)

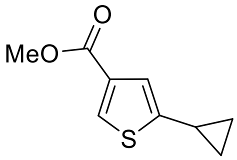

GP1 was performed using methyl 5-bromothiophene-3-carboxylate (**3**, 69.14 g, 0.313 mol), palladium acetate (1 mol%), SPhos (2 mol%). After distillation **3a** (50.48 g, 89%) was obtained as a colorless liquid.
(**3a**): bp = 91 °C (1 mbar); ^{1}H NMR (400 MHz, $CDCl_3$) δ 7.81 (d, *J* = 1.4 Hz, 1H), 7.14 (d, *J* = 1.1 Hz, 1H), 3.83 (s, 3H), 2.09 – 1.98 (m, 1H), 1.06 – 0.91 (m, 2H), 0.80 – 0.69 (m, 2H); ^{13}C NMR (100 MHz, $CDCl_3$) δ 163.4, 149.4, 133.0, 129.8, 123.0, 51.8, 11.1, 9.8; MS (EI) calcd for $C_9H_{10}O_2S$ [M^+]: 182; found 182.

Methyl 5-cyclopropylthiophene-2-carboxylate (4a)

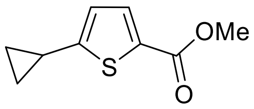

GP1 was performed using methyl 5-bromothiophene-2-carboxylate (**4**, 95.24 g, 0.430 mol), palladium acetate (1 mol%), SPhos (2 mol%). After distillation **4a** (67.80 g, 87%) was obtained as a colorless liquid.
(**4a**): bp = 81 °C (1 mbar); ^{1}H NMR (400 MHz, $CDCl_3$) δ 7.59 (d, *J* = 3.8 Hz, 1H), 6.74 (dd, *J* = 3.8, 0.7 Hz, 1H), 3.84 (s, 3H), 2.16 – 2.05 (m, 1H), 1.14 – 1.01 (m, 2H), 0.83 – 0.74 (m, 2H); ^{13}C NMR (100 MHz, $CDCl_3$) δ 162.8, 157.4, 133.9, 129.3, 123.5, 52.1, 12.0, 11.1; MS (EI) calcd for $C_9H_{10}O_2S$ [M^+]: 182; found 182.

Methyl 3-cyclopropylthiophene-2-carboxylate (5a)

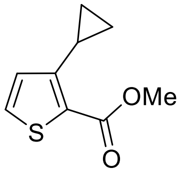

GP1 was performed using methyl 3-bromothiophene-2-carboxylate (**5**, 102.81 g, 0.465 mol), palladium acetate (1 mol%), SPhos (2 mol%). After distillation **5a** (71.13 g, 84%) was obtained as a colorless liquid.
(**5a**): bp = 78-80 °C (1 mbar); ^{1}H NMR (400 MHz, $CDCl_3$) δ 7.34 (dd, *J* = 5.2, 0.6 Hz, 1H), 6.55 (d, *J* = 5.2 Hz, 1H), 3.87 (s, 3H), 3.07 – 2.96 (m, 1H), 1.15 – 1.03 (m, 2H), 0.77 – 0.69 (m, 2H); ^{13}C NMR (100 MHz, $CDCl_3$) δ 163.6, 153.6, 130.8, 126.0, 125.0, 51.9, 10.8, 10.4; MS (EI) calcd for $C_9H_{10}O_2S$ [M^+]: 182; found 182.

Methyl 4-cyclopropylthiophene-2-carboxylate (6a)

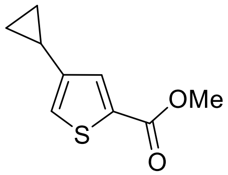

GP1 was performed using methyl 4-bromothiophene-2-carboxylate (**6**, 94.29 g, 0.431 mol), palladium acetate (1 mol%), SPhos (2 mol%). After distillation **6a** (68.41 g, 88%) was obtained as a colorless liquid.
(**6a**): bp = 80 °C (0.7 mbar); ^{1}H NMR (400 MHz, $CDCl_3$) δ 7.49 (d, *J* = 1.4 Hz, 1H), 7.09 (d, *J* = 1.5 Hz, 1H), 3.86 (s, 3H), 1.89 (tt, *J* = 8.4, 5.0 Hz, 1H), 0.99 – 0.86 (m, 2H), 0.68 – 0.59 (m, 2H); ^{13}C NMR (100 MHz, $CDCl_3$) δ 162.8, 146.1, 133.3, 132.3, 125.6, 52.2, 11.3, 8.7, data matched with literature [2]; MS (EI) calcd for $C_9H_{10}O_2S$ [M$^+$]: 182; found 182.

Methyl 2-cyclopropylthiophene-3-carboxylate (7a)

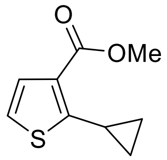

GP1 was performed using methyl 2-bromothiophene-3-carboxylate (**7**, 85.83 g, 0.388 mol), palladium acetate (0.5 mol%), SPhos (1 mol%). After distillation and recrystallization from hexanes **7a** (48.98 g, 69%) was obtained as a colorless low-melting crystalline solid.
(**7a**): bp = 70 °C (0.7 mbar); ^{1}H NMR (400 MHz, $CDCl_3$) δ 7.33 (d, *J* = 5.4 Hz, 1H), 6.90 (d, *J* = 5.4 Hz, 1H), 3.86 (s, 3H), 3.01 (tt, *J* = 8.4, 5.2 Hz, 1H), 1.22 – 1.10 (m, 2H), 0.82 – 0.72 (m, 2H); ^{13}C NMR (100 MHz, $CDCl_3$) δ 164.4, 159.4, 129.5, 128.6, 119.6, 51.5, 12.1, 11.7; MS (EI) calcd for $C_9H_{10}O_2S$ [M$^+$]: 182; found 182.

1-(4-Cyclopropylthiophen-2-yl)ethan-1-one (8a)

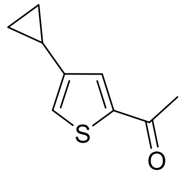

GP1 was performed using 1-(4-bromothiophen-2-yl)ethan-1-one (**8**, 98.0 g, 0.478 mol), palladium acetate (0.5 mol%), SPhos (1 mol%). After distillation **8a** (73.98 g, 93%) was obtained as a colorless liquid.
(**8a**): bp = 80 °C (0.5 mbar); ^{1}H NMR (400 MHz, $CDCl_3$) δ 7.41 (d, *J* = 1.6 Hz, 1H), 7.17 (d, *J* = 1.4 Hz, 1H), 2.52 (s, 3H), 1.95 – 1.84 (m, 1H), 1.00 – 0.88 (m, 2H), 0.69 – 0.59 (m, 2H); ^{13}C NMR (100 MHz, $CDCl_3$) δ 190.8, 146.4, 144.2, 131.5, 127.0, 26.9, 11.3, 8.7, data matched with literature [3]; MS (EI) calcd for $C_9H_{10}OS$ [M$^+$]: 166; found 166.

4-Cyclopropylthiophene-2-carbaldehyde (9a)

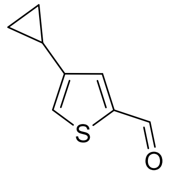

GP1 was performed using 4-bromothiophene-2-carbaldehyde (**9**, 293.81 g, 1.538 mol), palladium acetate (0.5 mol%), SPhos (1 mol%). After distillation **9a** (207.0 g, 89%) was obtained as a colorless liquid.
(**9a**): bp = 84 °C (1.2 mbar); ^{1}H NMR (400 MHz, $CDCl_3$) δ 9.81 (s, 1H), 7.47 (d, *J* = 1.5 Hz, 1H), 7.28 (d, *J* = 1.5 Hz, 1H), 1.97 – 1.86 (m, 1H), 0.99 – 0.88 (m, 2H), 0.68 – 0.59 (m, 2H), data matched with literature [4]; ^{13}C NMR (100 MHz, $CDCl_3$) δ 182.9, 146.7, 143.6, 135.0, 128.4, 11.1, 8.8; MS (EI) calcd for C_8H_8OS [M$^+$]: 152; found 152.

2-Cyclopropylthiophene-3-carbaldehyde (10a)

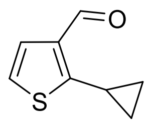

GP1 was performed using 2-bromothiophene-3-carbaldehyde (**10**, 64.0 g, 0.335 mol), palladium acetate (0.5 mol%), SPhos (1 mol%). After distillation **10a** (50.92 g, 86%) was obtained as a colorless liquid.
(**10a**): bp = 76-77 °C (2 mbar); ^{1}H NMR (400 MHz, $CDCl_3$) δ 10.17 (s, 1H), 7.32 (d, *J* = 5.4 Hz, 1H), 6.96 (d, *J* = 5.4 Hz, 1H), 2.67 (tt, *J* = 8.3, 5.0 Hz, 1H), 1.27 – 1.15 (m, 2H), 0.87 (dt, *J* = 6.9, 4.8 Hz, 2H); ^{13}C NMR (100 MHz, $CDCl_3$) δ 184.9, 161.9, 138.3, 127.3, 121.5, 11.7, 9.9; MS (EI) calcd for C_8H_8OS [M$^+$]: 152; found 152.

5-Cyclopropylthiophene-2-carbaldehyde (11a)

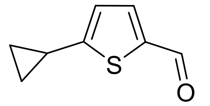

GP1 was performed using 5-bromothiophene-2-carbaldehyde (**11**, 200 g, 1.046 mol), palladium acetate (0.25 mol%), SPhos (0.5 mol%). After distillation **11a** (139.53 g, 88%) was obtained as a colorless liquid.
(**11a**): bp = 84 °C (0.6 mbar), lit. bp = 135 °C (18 mmHg) [1]; ^{1}H NMR (400 MHz, $CDCl_3$) δ 9.75 (s, 1H), 7.56 (d, *J* = 3.8 Hz, 1H), 6.84 (dd, *J* = 3.8, 0.6 Hz, 1H), 2.20 – 2.09 (m, 1H), 1.20 – 1.08 (m, 2H), 0.87 – 0.78 (m, 2H); ^{13}C NMR (100 MHz, $CDCl_3$) δ 182.4, 161.4, 140.2, 137.3, 123.9, 12.6, 11.9; MS (EI) calcd for C_8H_8OS [M$^+$]: 152; found 152.

3-Cyclopropylthiophene-2-carbaldehyde (12a)

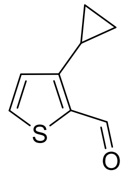

GP1 was performed using 3-bromothiophene-2-carbaldehyde (**12**, 148 g, 0.775 mol), palladium acetate (0.25 mol%), SPhos (0.5 mol%). After distillation **12a** (102.16 g, 87%) was obtained as a colorless liquid.
(**12a**): bp = 80 °C (1 mbar), lit. bp = 132 °C (10 mmHg) [1]; ^{1}H NMR (400 MHz, DMSO-d_6) δ 10.18 (s, 1H), 7.95 (dd, *J* = 5.1, 1.1 Hz, 1H), 6.82 (d, *J* = 5.1 Hz, 1H), 2.65 (tt, *J* = 8.3, 5.0 Hz, 1H), 1.10 (dt, *J* = 8.3, 3.2 Hz, 2H), 0.91 – 0.82 (m, 2H); ^{13}C NMR (100 MHz, DMSO-d_6) δ 183.3, 155.5, 137.1, 135.7, 126.3, 10.4, 9.8; MS (EI) calcd for C_8H_8OS [M$^+$]: 152; found 152.

4-Cyclopropylthiophene-3-carbaldehyde (13a)

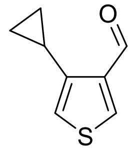

GP1 was performed using 4-bromothiophene-3-carbaldehyde (**13**, 148.35 g, 0.775 mol), palladium acetate (0.25 mol%), SPhos (0.5 mol%). After distillation **13a** (94.56 g, 80%) was obtained as a colorless liquid.
(**13a**): bp = 80 °C (1 mbar); ^{1}H NMR (400 MHz, $CDCl_3$) δ 10.09 (s, 1H), 8.05 (d, *J* = 3.2 Hz, 1H), 6.78 (d, *J* = 3.2 Hz, 1H), 2.46 – 2.32 (m, 1H), 1.03 – 0.91 (m, 2H), 0.69 – 0.60 (m, 2H); ^{13}C NMR (100 MHz, $CDCl_3$) δ 185.9, 145.0, 141.1, 138.2, 120.4, 9.7, 8.3; MS (EI) calcd for C_8H_8OS [M$^+$]: 152; found 152.

1. 2. 2. GP2. General procedure for the synthesis of cyclopropylthiophenecarbonitriles (15a-19a, 15b)
Hydroxylamine hydrochloride (1.4 eq.) was added in a single portion to a solution of cyclopropylthiophenecarboxaldehyde in pyridine (0.8 ml/mmol). The resulting mixture was heated at 90 °C

for 1h. Then it was cooled down to 60 °C and acetic anhydride (10 eq.) was added in a dropwise manner (caution: highly exothermic reaction!). After addition was completed, the reaction mixture was heated at 90 °C for 2 h, then cooled to room temperature and poured on to ice and acidified to pH = 5 with 6 M HCl. The product was extracted with hexanes, combined organic phases were washed with water, saturated sodium bicarbonate solution, dried with sodium sulfate, filtered, and concentrated *in vacuo*. The product was purified by vacuum distillation or crystallization. Small scale sample of compound **18a** was purified by flash chromatography.

4-Cyclopropylthiophene-2-carbonitrile (15a)

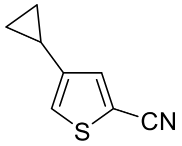

GP2 was performed using 4-cyclopropylthiophene-2-carbaldehyde (**9a**, 40.0 g, 0.263 mol). After distillation **15a** (38.0 g, 97%) was obtained as a colorless liquid.

(**15a**): bp = 90 °C (2 mbar); ^{1}H NMR (400 MHz, $CDCl_3$) δ 7.32 (d, *J* = 1.5 Hz, 1H), 7.11 (d, *J* = 1.5 Hz, 1H), 1.90 (tt, *J* = 8.4, 5.0 Hz, 1H), 1.03 – 0.90 (m, 2H), 0.64 (dt, *J* = 6.7, 4.8 Hz, 2H); ^{13}C NMR (100 MHz, $CDCl_3$) δ 146.2, 136.5, 125.5, 114.6, 109.6, 11.1, 8.9; MS (EI) calcd for C_8H_7NS [M^+]: 149; found 149.

2-Cyclopropylthiophene-3-carbonitrile (16a)

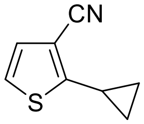

GP2 was performed using 2-cyclopropylthiophene-3-carbaldehyde (**10a**, 41.87 g, 0.275 mol). After distillation **16a** (38.5 g, 94%) was obtained as a colorless liquid.

(**16a**): bp = 70 °C (1.8 mbar); ^{1}H NMR (400 MHz, $CDCl_3$) δ 7.09 – 6.99 (m, 2H), 2.35 (tt, *J* = 8.3, 5.0 Hz, 1H), 1.29 – 1.16 (m, 2H), 0.87 (dt, *J* = 6.8, 4.8 Hz, 2H); ^{13}C NMR (100 MHz, $CDCl_3$) δ 161.0, 128.5, 122.3, 115.5, 108.2, 11.6, 11.5; MS (EI) calcd for C_8H_7NS [M^+]: 149; found 149.

5-Cyclopropylthiophene-2-carbonitrile (17a)

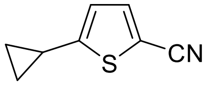

GP2 was performed using 5-cyclopropylthiophene-2-carbaldehyde (**11a**, 31.5 g, 0.207 mol). After distillation **17a** (27.75 g, 90%) was obtained as a colorless liquid.

(**17a**): bp = 78 °C (2 mbar); ^{1}H NMR (400 MHz, $CDCl_3$) δ 7.41 (d, *J* = 3.8 Hz, 1H), 6.74 (d, *J* = 3.8 Hz, 1H), 2.12 (tt, *J* = 8.3, 5.0 Hz, 1H), 1.18 – 1.05 (m, 2H), 0.80 (dt, *J* = 6.8, 4.8 Hz, 2H); ^{13}C NMR (100 MHz, $CDCl_3$) δ 157.6, 137.8, 123.2, 114.8, 105.6, 11.7, 11.2; MS (EI) calcd for C_8H_7NS [M^+]: 149; found 149.

3-Cyclopropylthiophene-2-carbonitrile (18a)

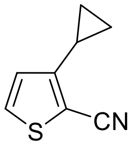

GP2 was performed using 3-cyclopropylthiophene-2-carbaldehyde (**12a**, 1 g, 6.5 mmol). After flash chromatography on silica gel (DCM-hexanes 2:3) 18**a** (0.83 g, 84%) was obtained as a colorless liquid.

(**18a**): ^1H NMR (400 MHz, $CDCl_3$) δ 7.42 (d, *J* = 5.2 Hz, 1H), 6.59 (d, *J* = 5.2 Hz, 1H), 2.18 (tt, *J* = 8.4, 5.0 Hz, 1H), 1.22 – 1.08 (m, 2H), 0.82 (dt, *J* = 6.7, 4.8 Hz, 2H); ^{13}C NMR (100 MHz, $CDCl_3$) δ 156.8, 132.0, 124.4, 114.7, 104.3, 11.7, 10.1; MS (EI) calcd for C_8H_7NS [M^+]: 149; found 149.

4-Cyclopropylthiophene-3-carbonitrile (19a)

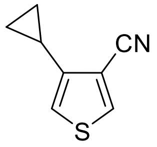

GP2 was performed using 4-cyclopropylthiophene-3-carbaldehyde (**13a**, 36.94 g, 0.243 mol). After distillation **19a** (33.36 g, 92%) was obtained as a colorless liquid.
(**19a**): bp = 66-67 °C (0.7 mbar); ^1H NMR (400 MHz, $CDCl_3$) δ 7.84 (d, *J* = 3.2 Hz, 1H), 6.80 (dd, *J* = 3.1, 0.9 Hz, 1H), 2.07 – 1.96 (m, 1H), 1.08 – 0.95 (m, 2H), 0.77 – 0.65 (m, 2H); ^{13}C NMR (100 MHz, $CDCl_3$) δ 146.5, 135.6, 119.4, 115.2, 112.7, 10.0, 8.3; MS (EI) calcd for C_8H_7NS [M^+]: 149; found 149.

5-Bromo-4-cyclopropylthiophene-2-carbonitrile (15b)

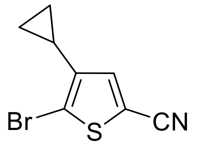

GP2 was performed using 5-bromo-4-cyclopropylthiophene-2-carbaldehyde (**9b**, 27.3 g, 0.118 mol). After recrystallization from hexanes **15b** (24.18 g, 90%) was obtained as a colorless crystalline solid.
(**15b**): mp = 44.8 °C; ^1H NMR (400 MHz, $CDCl_3$) δ 6.95 (s, 1H), 1.97 – 1.86 (m, 1H), 1.11 – 0.99 (m, 2H), 0.70 – 0.62 (m, 2H); ^{13}C NMR (100 MHz, $CDCl_3$) δ 144.9, 135.0, 116.6, 113.7, 109.6, 10.9, 8.6; MS (EI) calcd for C_8H_6BrNS [M^+]: 227/229; found 227/229.

1. 2. 3. GP3. General procedure for the hydrolysis of esters (3a, 5a, 6a)

Methyl cyclopropylthiophene carboxylate was added as a neat liquid to a solution of sodium hydroxide (1 M, 2 eq.). The reaction mixture was then heated at 50 °C for 2-3 h. After the consumption of starting material, reaction mixture was cooled down to room temperature. The reaction mixture was then acidified with 6 M HCl to pH = 2. Product was filtered off, washed with deionized water, and dried in vacuum.

5-Cyclopropylthiophene-3-carboxylic acid (20a)

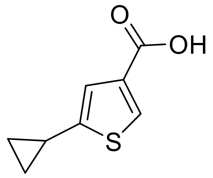

GP3 was performed using methyl 5-cyclopropylthiophene-3-carboxylate (**3a**, 50.48 g, 0.277 mol). After drying in vacuum **20a** (46.57 g, 99.8%) was obtained as a white solid.
(**20a**): mp = 81.9 °C; ^1H NMR (400 MHz, $CDCl_3$) δ 11.51 (s, 1H), 7.95 (d, *J* = 1.3 Hz, 1H), 7.21 – 7.16 (m, 1H), 2.12 – 2.00 (m, 1H), 1.08 – 0.96 (m, 2H), 0.80 – 0.71 (m, 2H); ^{13}C NMR (100 MHz, $CDCl_3$) δ 168.4, 149.8, 132.4, 131.9, 123.2, 11.1, 9.9.

3-Cyclopropylthiophene-2-carboxylic acid (21a)

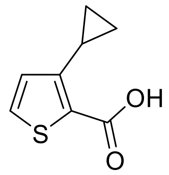

GP3 was performed using methyl 3-cyclopropylthiophene-2-carboxylate (**5a**, 45.0 g, 0.246 mol). After drying in vacuum **21a** (40.75 g, 98%) was obtained as a white solid.
(**21a**): mp = 170.1 °C; ^{1}H NMR (400 MHz, $CDCl_3$) δ 11.10 (s, 1H), 7.45 (d, *J* = 5.2 Hz, 1H), 6.58 (d, *J* = 5.2 Hz, 1H), 3.08 – 2.96 (m, 1H), 1.18 – 1.06 (m, 2H), 0.82 – 0.72 (m, 2H); ^{13}C NMR (100 MHz, $CDCl_3$) δ 169.0, 155.3, 132.5, 125.7, 125.3, 11.1, 10.8.

4-Cyclopropylthiophene-2-carboxylic acid (22a)

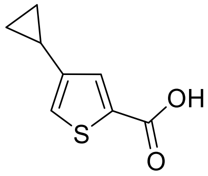

GP3 was performed using methyl 4-cyclopropylthiophene-2-carboxylate (**6a**, 68.41 g, 0.375 mol). After drying in vacuum **22a** (61.86 g, 98%) was obtained as a white solid.
(**22a**): mp = 110.6 °C; ^{1}H NMR (400 MHz, DMSO-d_6) δ 12.99 (s, 1H), 7.48 – 7.41 (m, 2H), 1.95 (tt, *J* = 8.4, 5.0 Hz, 1H), 0.95 – 0.82 (m, 2H), 0.68 – 0.60 (m, 2H); ^{13}C NMR (100 MHz, DMSO-d_6) δ 162.9, 145.8, 134.3, 131.6, 125.6, 11.0, 8.9.

1. 2. 4. GP4. General procedure for the bromination of cyclopropylthiophenes 1a and 2a

N-bromosuccinimide (NBS, 1 eq.) was added in portions to a cooled solution of cyclopropylthiophene (1 eq.) in a mixture of dichloromethane (1.14 ml/mmol) and glacial acetic acid (1.14 ml/mmol) maintaining the temperature below 15 °C. After complete consumption of the starting material, the reaction mixture was transferred to a separatory funnel, washed with water, and saturated sodium bicarbonate solution. The organic phase was dried with sodium sulfate, filtered, and concentrated *in vacuo*. The product was purified by vacuum distillation.

2-Bromo-5-cyclopropylthiophene (1b)

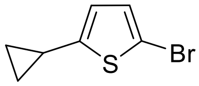

GP4 was performed using 2-cyclopropylthiophene (**1a**, 59.29 g, 0.477 mol). After distillation **1b** (58.26 g, 72%) was obtained as a colorless liquid.
(**1b**): bp = 65 °C (2.6 mbar), lit. bp = 106 °C (14 mmHg) [1]; ^{1}H NMR (400 MHz, $CDCl_3$) δ 6.81 (d, *J* = 3.7 Hz, 1H), 6.52 (dd, *J* = 3.7, 1.0 Hz, 1H), 2.06 – 1.94 (m, 1H), 1.05 – 0.88 (m, 2H), 0.76 – 0.62 (m, 2H); ^{13}C NMR (100 MHz, $CDCl_3$) δ 150.3, 129.5, 123.3, 107.8, 11.3, 9.5; MS (EI) calcd for C_7H_7BrS [M^+]: 204/206; found 204/206.

2-Bromo-3-cyclopropylthiophene (2b)

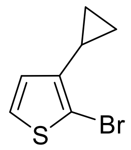

GP4 was performed using 3-cyclopropylthiophene (**2a**, 71.3 g, 0.574 mol). After distillation **2b** (110.16 g, 94%) was obtained as a colorless liquid.

(**2b**): bp = 75 °C (3.3 mbar), lit bp = 97 °C (10 mmHg) [1]; ^{1}H NMR (400 MHz, $CDCl_3$) δ 7.15 (d, *J* = 5.6 Hz, 1H), 6.44 (d, *J* = 5.7 Hz, 1H), 2.01 – 1.88 (m, 1H), 1.07 – 0.93 (m, 2H), 0.73 – 0.58 (m, 2H); ^{13}C NMR (100 MHz, $CDCl_3$) δ 143.3, 125.4, 124.6, 108.5, 11.0, 8.3; MS (EI) calcd for C_7H_7BrS [M^+]: 204/206; found 204/206.

1. 2. 5. GP5. General procedure for the synthesis of cyclopropylthiophenes 6b-9b, 16b, 20b, 22b

Sodium acetate trihydrate (1 eq.) was added to a solution of cyclopropylthiophene (1 eq.) in glacial acetic acid (1.43 ml/mmol) at 15 °C. A solution of elemental bromine in glacial acetic acid (1.5 M, 1.05 eq.) was added dropwise maintaining the reaction temperature below 20 °C. After complete addition of bromine, the reaction mixture was stirred at room temperature for 12 h and then poured into water.

For compounds 6b – 9b, 16b: products were extracted with hexanes, combined organic phases were washed with water, saturated sodium bicarbonate solution, dried with sodium sulfate, and filtered. After removal of solvents *in vacuo* product was purified by crystallization from hexanes.

For compounds 20b and 22b: products were collected by filtration and washed with deionized water.

Methyl 5-bromo-4-cyclopropylthiophene-2-carboxylate (6b)

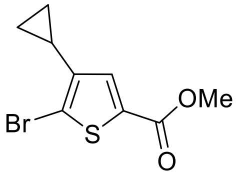

GP5 was performed using methyl 4-cyclopropylthiophene-2-carboxylate (**6a**, 25 g, 0.137 mol). After recrystallization from hexanes **6b** (26.51 g, 74%) was obtained as a colorless crystalline solid.

(**6b**): mp = 67.7 °C; ^{1}H NMR (400 MHz, $CDCl_3$) δ 7.13 (d, *J* = 2.5 Hz, 1H), 3.84 (s, 3H), 1.97 – 1.85 (m, 1H), 1.06 – 0.95 (m, 2H), 0.73 – 0.60 (m, 2H); ^{13}C NMR (100 MHz, $CDCl_3$) δ 161.9, 144.9, 132.5, 130.6, 116.9, 52.4, 11.0, 8.6; MS (EI) calcd for $C_9H_9BrO_2S$ [M^+]: 260/262; found 260/262.

Methyl 5-bromo-2-cyclopropylthiophene-3-carboxylate (7b)

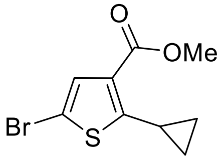

GP5 was performed using methyl 2-cyclopropylthiophene-3-carboxylate (**7a**, 20.33 g, 0.112 mol). After recrystallization from hexanes **7b** (21.64 g, 74%) was obtained as a colorless crystalline solid.

(**7b**): mp = 28.1 °C; ^{1}H NMR (400 MHz, $CDCl_3$) δ 7.28 (s, 1H), 3.84 (s, 3H), 2.95 (tt, *J* = 8.4, 5.2 Hz, 1H), 1.21 – 1.09 (m, 2H), 0.73 (dt, *J* = 6.8, 4.9 Hz, 2H); ^{13}C NMR (100 MHz, $CDCl_3$) δ 163.1, 160.7, 131.8, 129.1, 106.2, 51.7, 12.0, 11.9; MS (EI) calcd for $C_9H_9BrO_2S$ [M^+]: 260/262; found 260/262.

1-(5-Bromo-4-cyclopropylthiophen-2-yl)ethan-1-one (8b)

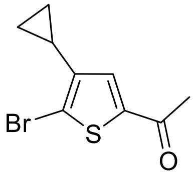

GP5 was performed using 1-(4-cyclopropylthiophen-2-yl)ethan-1-one (**8a**, 30 g, 0.180 mol). After recrystallization from hexanes **8b** (38.63 g, 87%) was obtained as a colorless crystalline solid.

(**8b**): mp = 69.7 °C; ^{1}H NMR (400 MHz, $CDCl_3$) δ 7.02 (s, 1H), 2.46 (s, 3H), 1.97 – 1.86 (m, 1H), 1.08 – 0.97 (m, 2H), 0.72 – 0.63 (m, 2H); ^{13}C NMR (100 MHz, $CDCl_3$) δ 189.7, 145.1, 143.6, 129.5, 119.6, 26.4, 11.0, 8.4; MS (EI) calcd for C_9H_9BrOS [M$^+$]: 244/246; found 244/246.

5-Bromo-4-cyclopropylthiophene-2-carbaldehyde (9b)

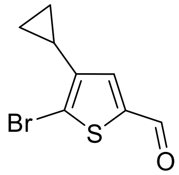

GP5 was performed using 4-cyclopropylthiophene-2-carbaldehyde (**9a**, 46 g, 0.302 mol). After recrystallization from hexanes **9b** (55.94 g, 80%) was obtained as a colorless crystalline solid.
(**9b**): mp = 39.0 °C; ^{1}H NMR (400 MHz, $CDCl_3$) δ 9.70 (s, 1H), 7.11 (s, 1H), 1.94 (tt, *J* = 8.4, 5.1 Hz, 1H), 1.12 – 0.99 (m, 2H), 0.73 – 0.65 (m, 2H); ^{13}C NMR (100 MHz, $CDCl_3$) δ 181.9, 145.5, 142.9, 133.3, 121.9, 11.0, 8.6; MS (EI) calcd for C_8H_7BrOS [M$^+$]: 230/232; found 230/232.

5-Bromo-2-cyclopropylthiophene-3-carbonitrile (16b)

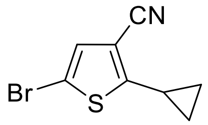

GP5 was performed using 2-cyclopropylthiophene-3-carbonitrile (**16a**, 11.73 g, 0.078 mol). After recrystallization from hexanes **16b** (16.35 g, 91%) was obtained as a colorless crystalline solid.
(**16b**): mp = 58.2 - 58.8 °C; ^{1}H NMR (400 MHz, $CDCl_3$) δ 7.01 (s, 1H), 2.30 (tt, *J* = 8.2, 4.9 Hz, 1H), 1.29 – 1.16 (m, 2H), 0.91 – 0.81 (m, 2H) ; ^{13}C NMR (100 MHz, $CDCl_3$) δ 162.4, 130.3, 114.1, 108.7, 108.6, 11.8, 11.5 ; MS (EI) calcd for C_8H_6BrNS [M$^+$]: 227/229; found 227/229.

2-Bromo-5-cyclopropylthiophene-3-carboxylic acid (20b)

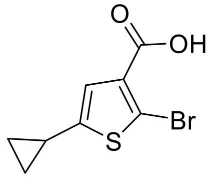

GP5 was performed using 5-cyclopropylthiophene-3-carboxylic acid (**20a**, 13.05 g, 0.078 mol). After drying in vacuum **20b** (16.06 g, 84%) was obtained as a white solid.
(**20b**): mp = 146.6 °C; ^{1}H NMR (400 MHz, DMSO-*d*$_6$) δ 13.03 (s, 1H), 7.01 (s, 1H), 2.16 – 2.04 (m, 1H), 1.11 – 0.91 (m, 2H), 0.85 – 0.59 (m, 2H); ^{13}C NMR (101 MHz, DMSO-*d*$_6$) δ 162.6, 148.4, 131.7, 124.4, 114.0, 10.8, 9.7.

5-Bromo-4-cyclopropylthiophene-2-carboxylic acid (22b)

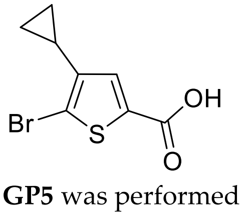

GP5 was performed using 4-cyclopropylthiophene-2-carboxylic acid (**22a**, 20 g, 0.118 mol). After drying in vacuum **22b** (28.07 g, 96%) was obtained as a white solid.

(**22b**): mp = 180.3 °C; ^{1}H NMR (400 MHz, DMSO-d_6) δ 7.18 (d, *J* = 1.1 Hz, 1H), 3.55 (s, 1H), 1.86 (tt, *J* = 8.6, 5.1 Hz, 1H), 1.04 – 0.91 (m, 2H), 0.77 – 0.66 (m, 2H); ^{13}C NMR (100 MHz, DMSO-d_6) δ 161.9, 144.9, 134.2, 130.1, 114.7, 10.9, 8.5.

1. 2. 6. GP6. General procedure for the synthesis of cyclopropylthiophenesulfochlorides 23a-25a

n-BuLi (2.5 M in hexanes, 1.1 eq.) was added dropwise to a solution of cyclopropylthiophene (1 eq.) in THF (3 ml/mmol) while maintaining reaction temperature at -78 °C. The reaction mixture was stirred at -78 °C for 40 min to ensure complete lithiation. Liquefied sulfur dioxide (2 eq.) was then added dropwise via cannula to the reaction mixture while keeping the temperature below -60 °C and stirred for an additional 30 min. *N*-chlorosuccinimide (NCS) (1 eq.) was then added in a single portion and the mixture was allowed to warm-up to room temperature. Solvents were removed on a rotary evaporator. The remaining residue was suspended in hexanes, filtered through a layer of Celite® and concentrated *in vacuo*. The product was purified by recrystallization from minimal amount of hexanes at 0 °C.

5-Cyclopropylthiophene-2-sulfonyl chloride (23a)

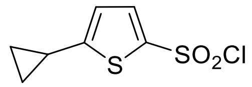

GP6 was performed using 2-cyclopropylthiophene (**1a**, 30 g, 0.242 mol). After recrystallization from hexanes **23a** (32.59 g, 60%) was obtained as an off-white crystalline solid.
(**23a**): mp = 38.7 °C; ^{1}H NMR (400 MHz, $CDCl_3$) δ 7.68 (d, *J* = 4.0 Hz, 1H), 6.80 (d, *J* = 4.1 Hz, 1H), 2.17 (tt, *J* = 8.3, 5.0 Hz, 1H), 1.28 – 1.15 (m, 2H), 0.87 (dt, *J* = 7.0, 4.8 Hz, 2H); ^{13}C NMR (100 MHz, $CDCl_3$) δ 162.4, 138.7, 135.6, 123.2, 12.2, 12.0; MS (EI) calcd for $C_7H_7ClO_2S_2$ [M^+]: 222; found 222.

4-Cyclopropylthiophene-2-sulfonyl chloride (24a)

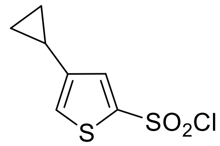

GP6 was performed using 3-cyclopropylthiophene (**2a**, 30 g, 0.242 mol). After recrystallization from hexanes **24a** (26.0 g, 48%) was obtained as an off-white crystalline solid.
(**24a**): mp = 37.2 °C; ^{1}H NMR (400 MHz, $CDCl_3$) δ 7.58 (d, *J* = 1.7 Hz, 1H), 7.35 (d, *J* = 1.7 Hz, 1H), 1.94 (tt, *J* = 8.3, 5.0 Hz, 1H), 1.08 – 0.95 (m, 2H), 0.76 – 0.61 (m, 2H); ^{13}C NMR (100 MHz, $CDCl_3$) δ 146.5, 143.5, 133.7, 128.9, 11.3, 9.1; MS (EI) calcd for MS (EI) $C_7H_7ClO_2S_2$ [M^+]: 222; found 222.

3-Cyclopropylthiophene-2-sulfonyl chloride (25a)

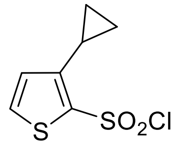

GP6 was performed using 2-bromo-3-cyclopropylthiophene (**2b**, 50 g, 0.253 mol). After recrystallization from hexanes **25a** (25.5 g, 55%) was obtained as an off-white crystalline solid.
(**25a**): mp = 35.7 °C; ^{1}H NMR (400 MHz, $CDCl_3$) δ 7.62 (dd, *J* = 5.3, 0.6 Hz, 1H), 6.63 (d, *J* = 5.3 Hz, 1H), 2.67 (tt, *J* = 8.4, 5.0 Hz, 1H), 1.32 – 1.20 (m, 2H), 0.93 – 0.84 (m, 2H); ^{13}C NMR (100 MHz, $CDCl_3$) δ 153.8, 137.3, 134.1, 125.6, 11.2, 10.7; MS (EI) calcd for $C_7H_7ClO_2S_2$ [M^+]: 222; found 222.

2. NMR spectral outtakes

2-Cyclopropylthiophene (1a)

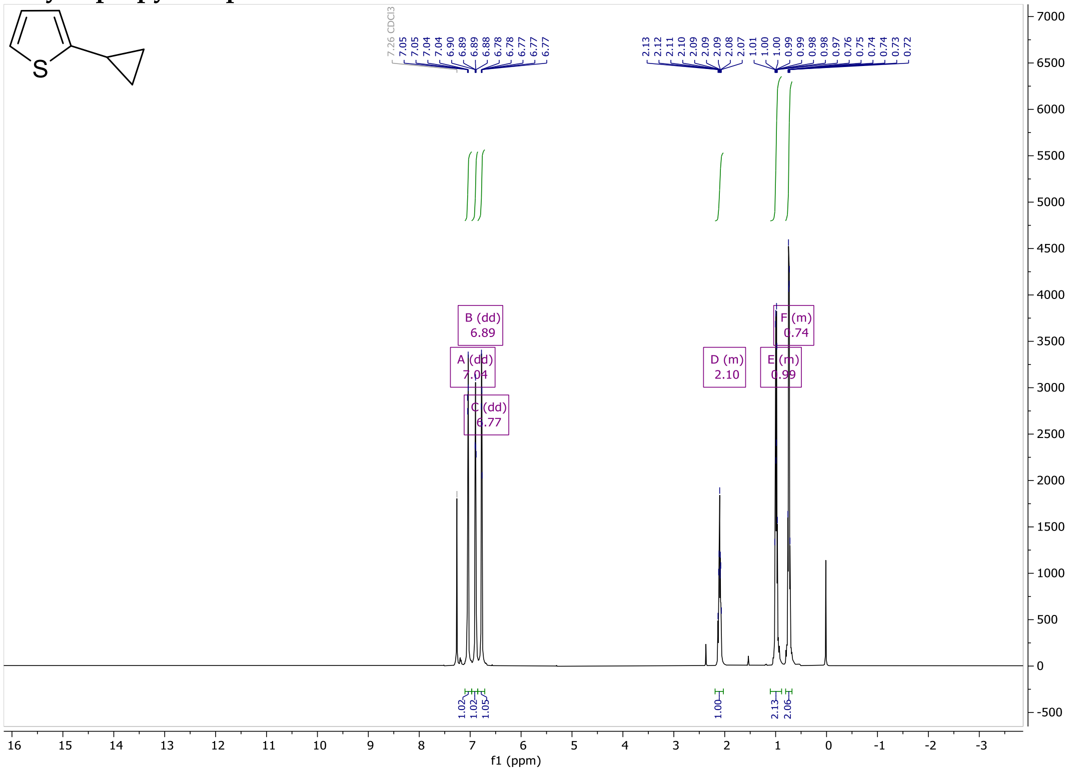

Figure S 1 ^{1}H NMR spectra of compound **1a** in $CDCl_3$ (400 MHz)

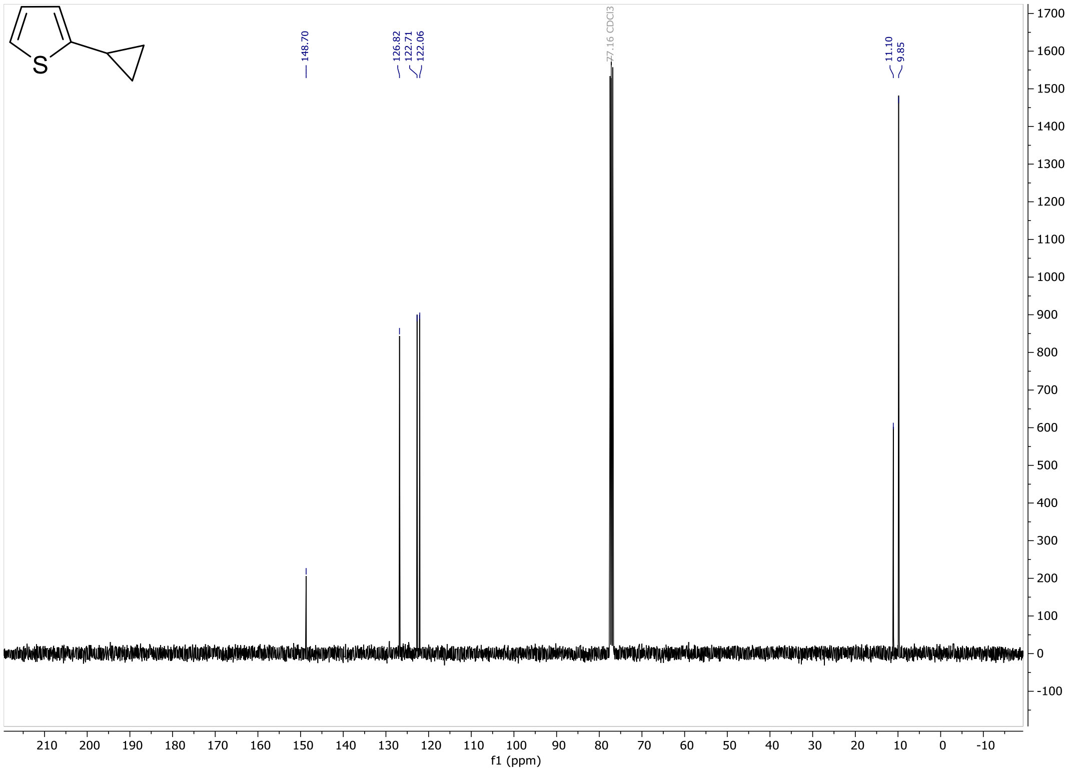

Figure S 2 ^{13}C NMR spectra of compound **1a** in $CDCl_3$ (100 MHz)

3-Cyclopropylthiophene (2a)

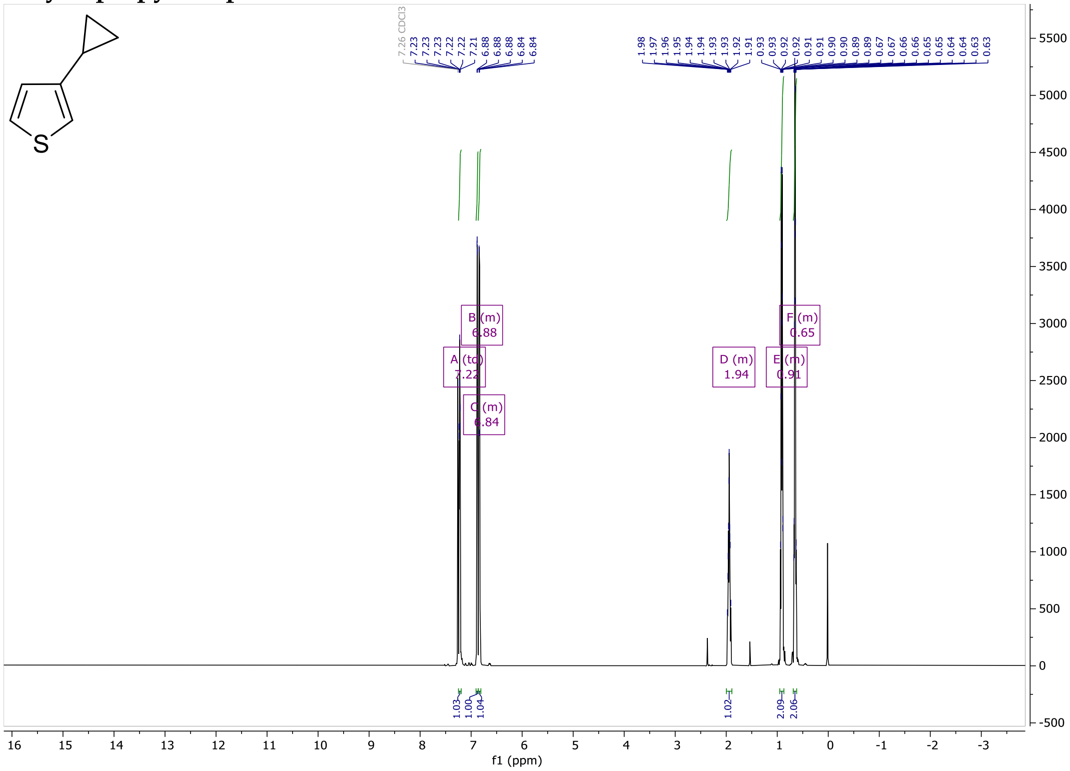

Figure S 3 ^{1}H NMR spectra of compound **2a** in $CDCl_3$ (400 MHz)

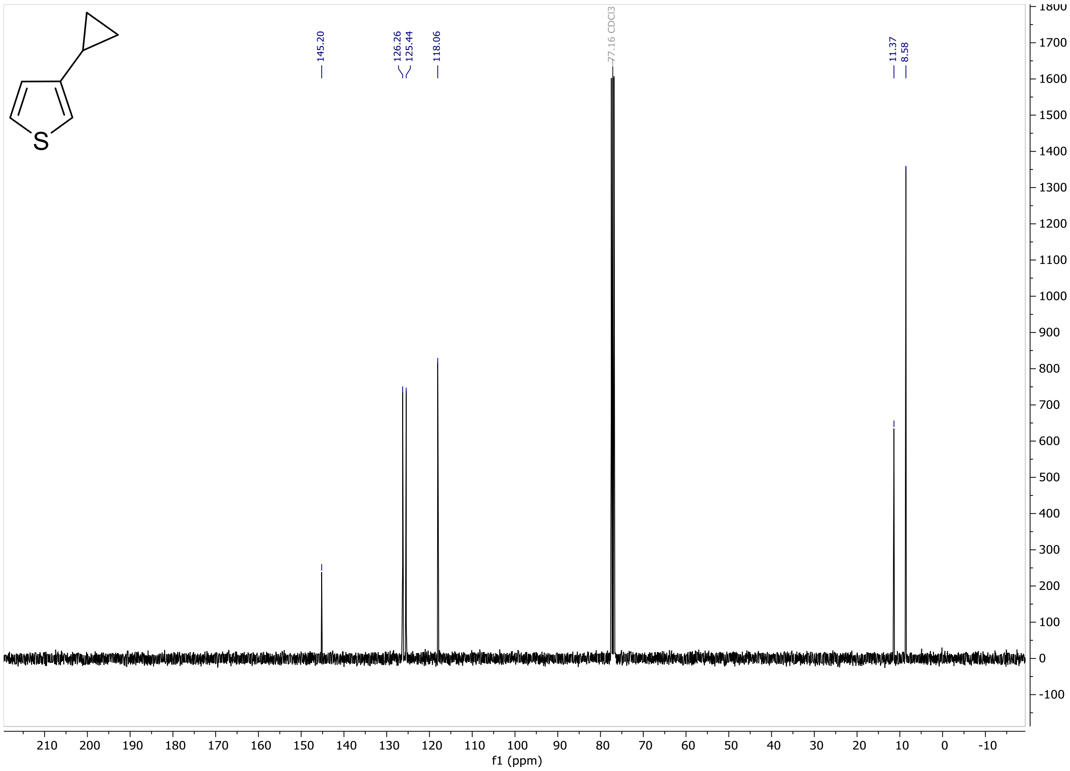

Figure S 4 ^{13}C NMR spectra of compound **2a** in $CDCl_3$ (100 MHz)

Methyl 5-cyclopropylthiophene-3-carboxylate (3a)

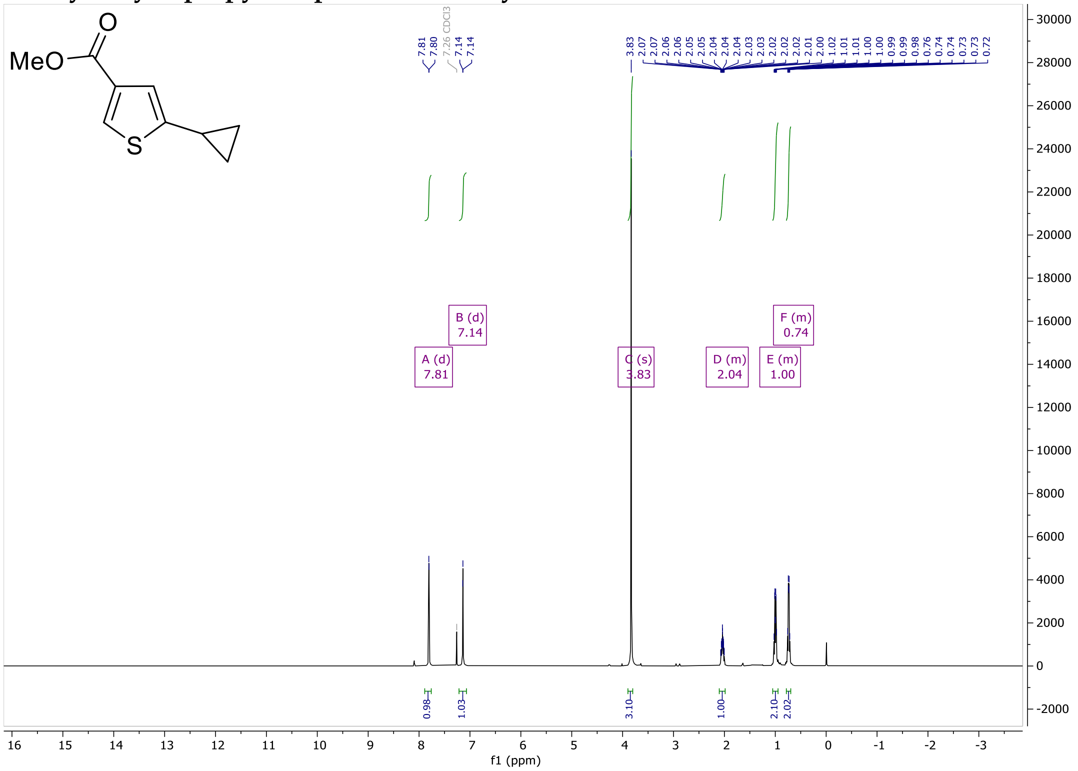

Figure S 5 ^{1}H NMR spectra of compound **3a** in $CDCl_3$ (400 MHz)

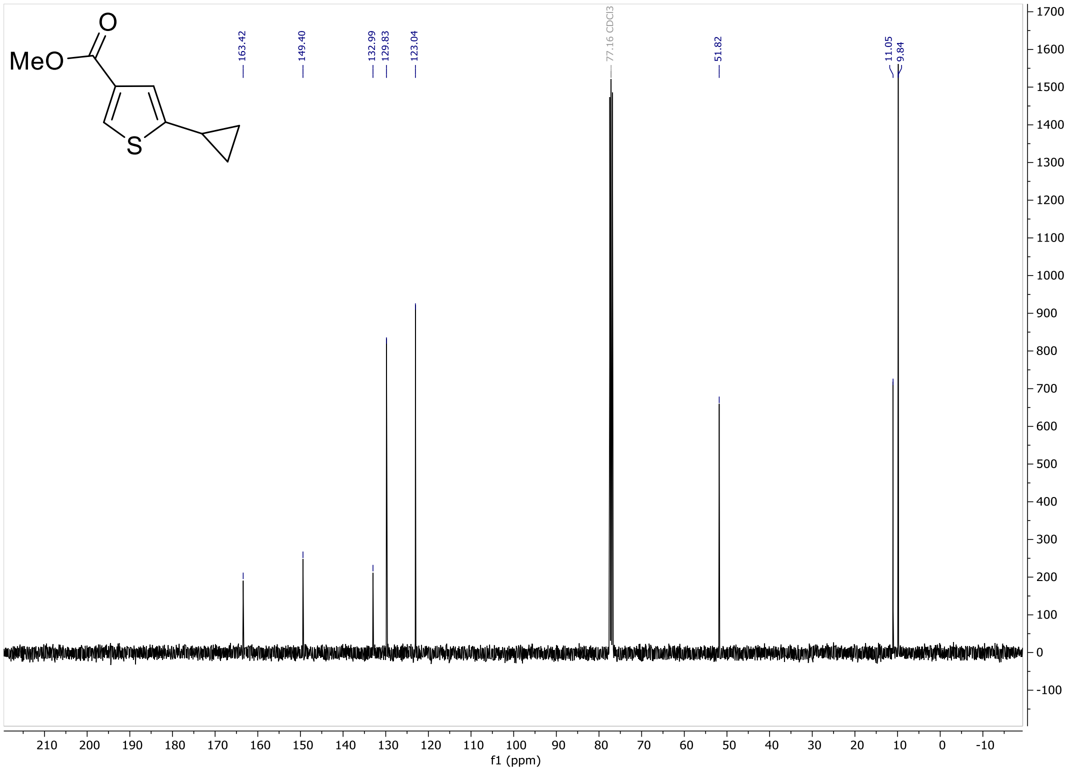

Figure S 6 ^{13}C NMR spectra of compound **3a** in $CDCl_3$ (100 MHz)

Methyl 5-cyclopropylthiophene-2-carboxylate (4a)

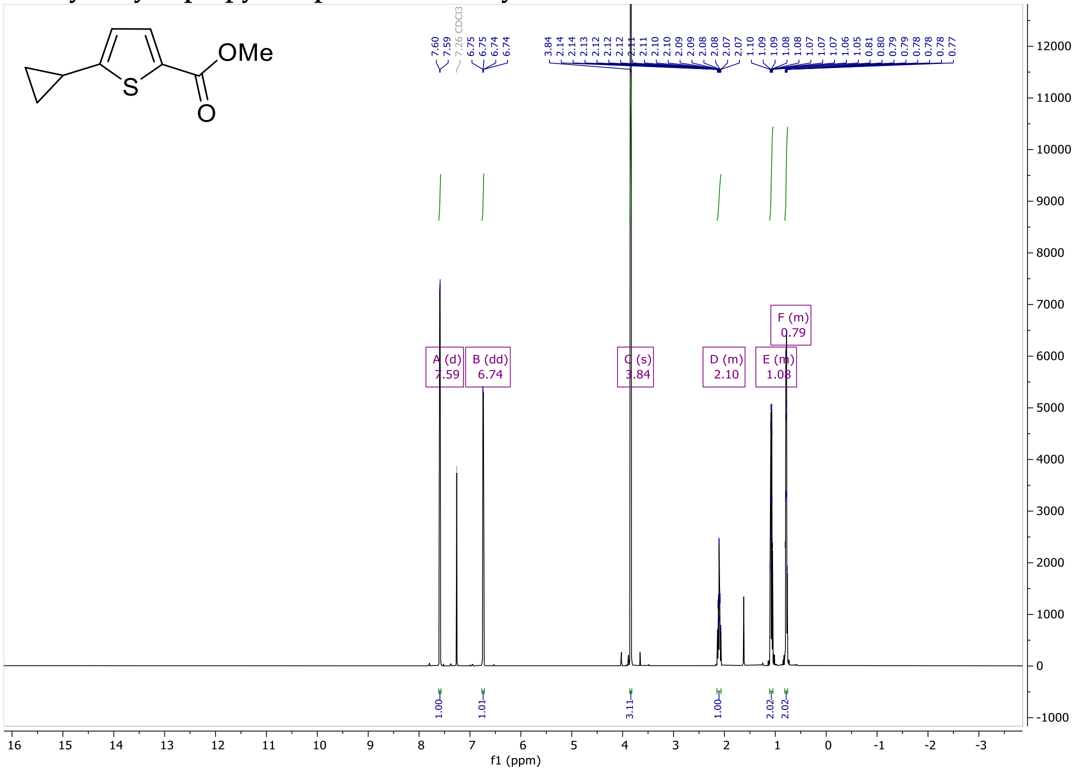

Figure S 7 ^{1}H NMR spectra of compound **4a** in $CDCl_3$ (400 MHz)

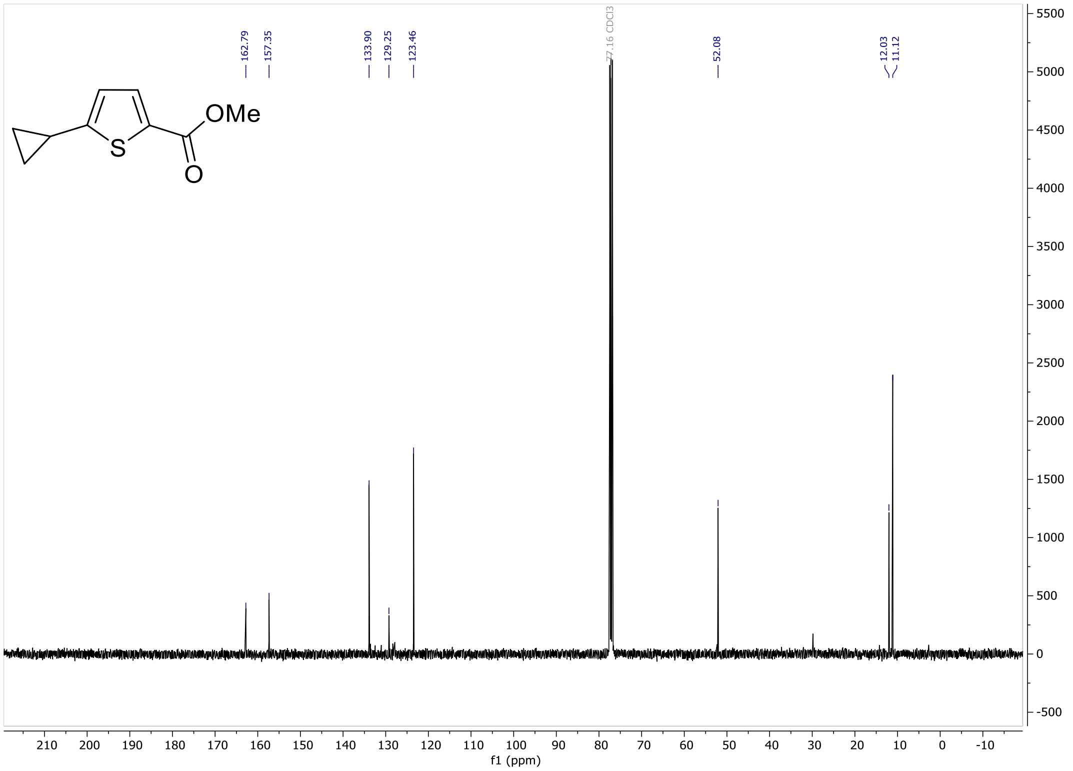

Figure S 8 ^{13}C NMR spectra of compound **4a** in $CDCl_3$ (100 MHz)

Methyl 3-cyclopropylthiophene-2-carboxylate (5a)

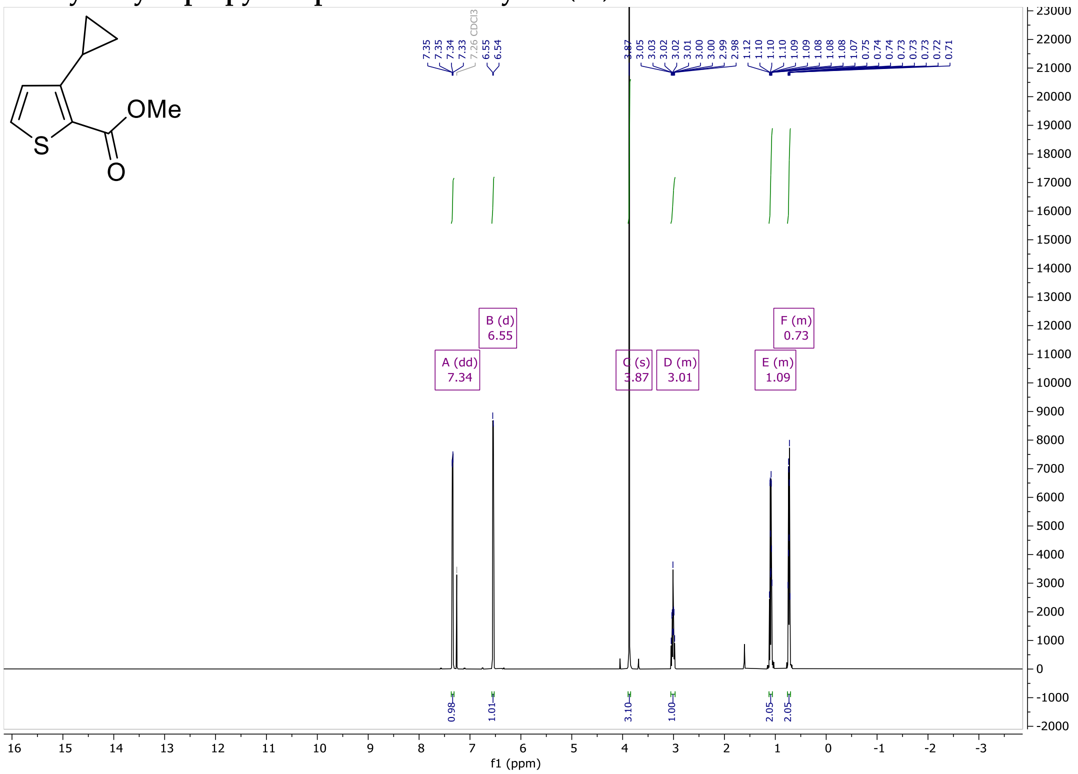

Figure S 9 ^{1}H NMR spectra of compound **5a** in $CDCl_3$ (400 MHz)

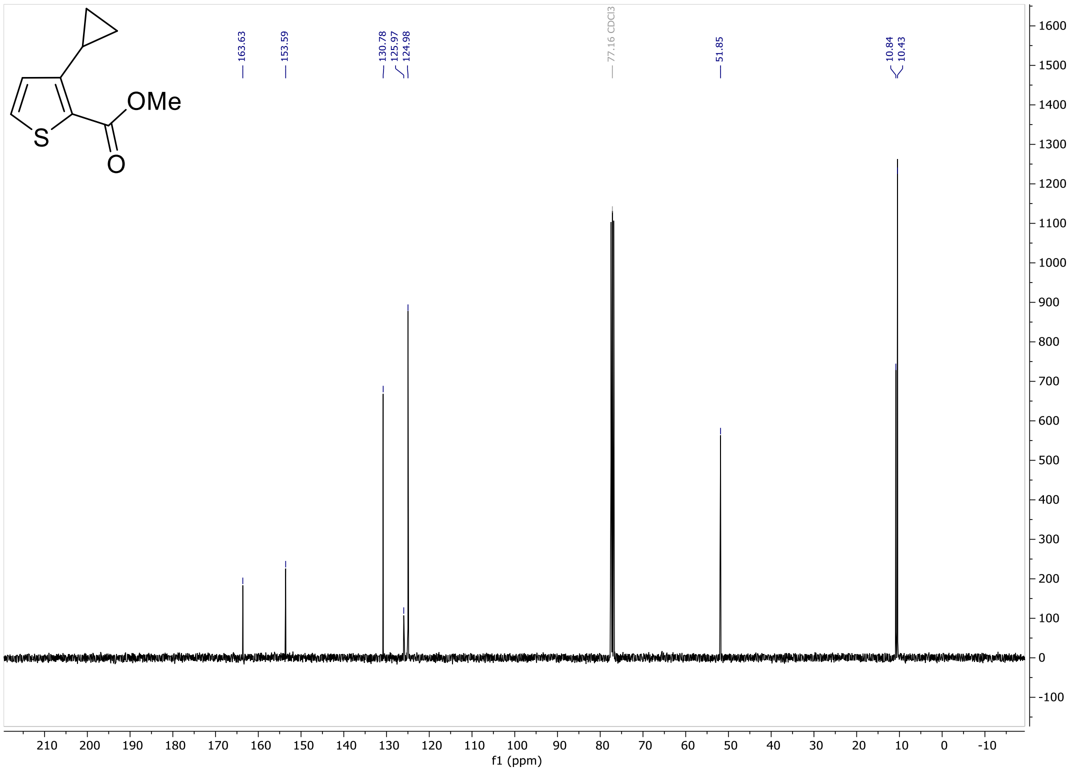

Figure S 10 ^{13}C NMR spectra of compound **5a** in $CDCl_3$ (100 MHz)

Methyl 4-cyclopropylthiophene-2-carboxylate (6a)

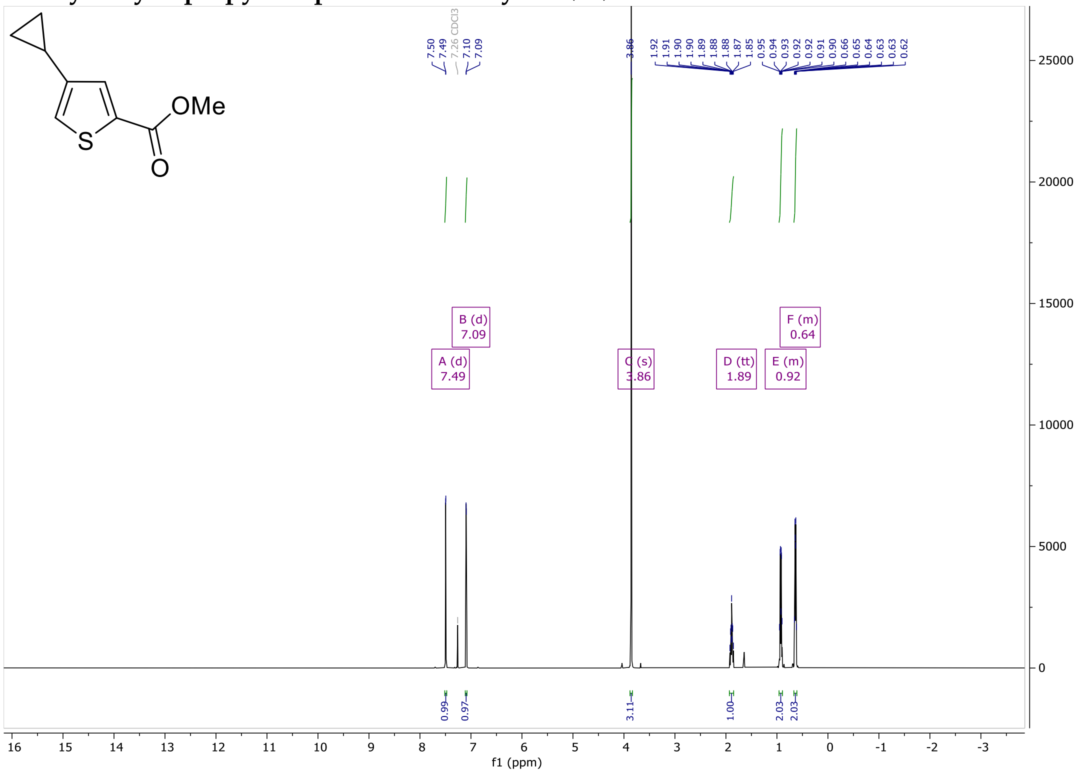

Figure S 11 ^{1}H NMR spectra of compound **6a** in $CDCl_3$ (400 MHz)

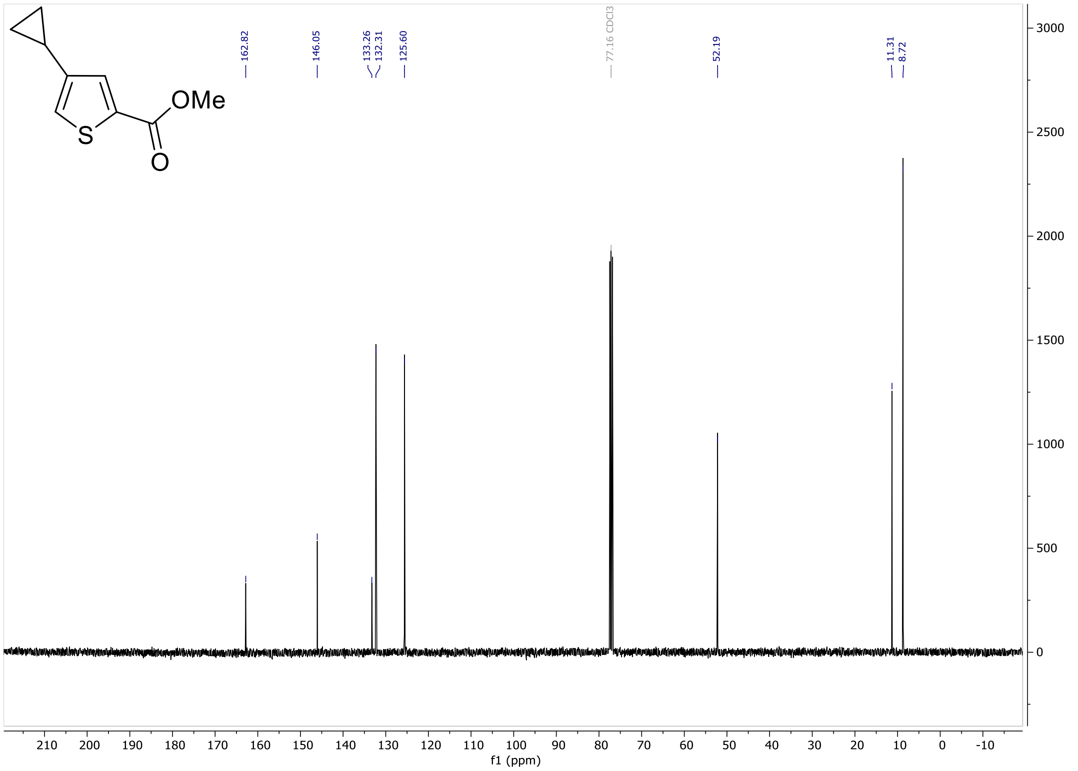

Figure S 12 ^{13}C NMR spectra of compound **6a** in $CDCl_3$ (100 MHz)

Methyl 2-cyclopropylthiophene-3-carboxylate (7a)

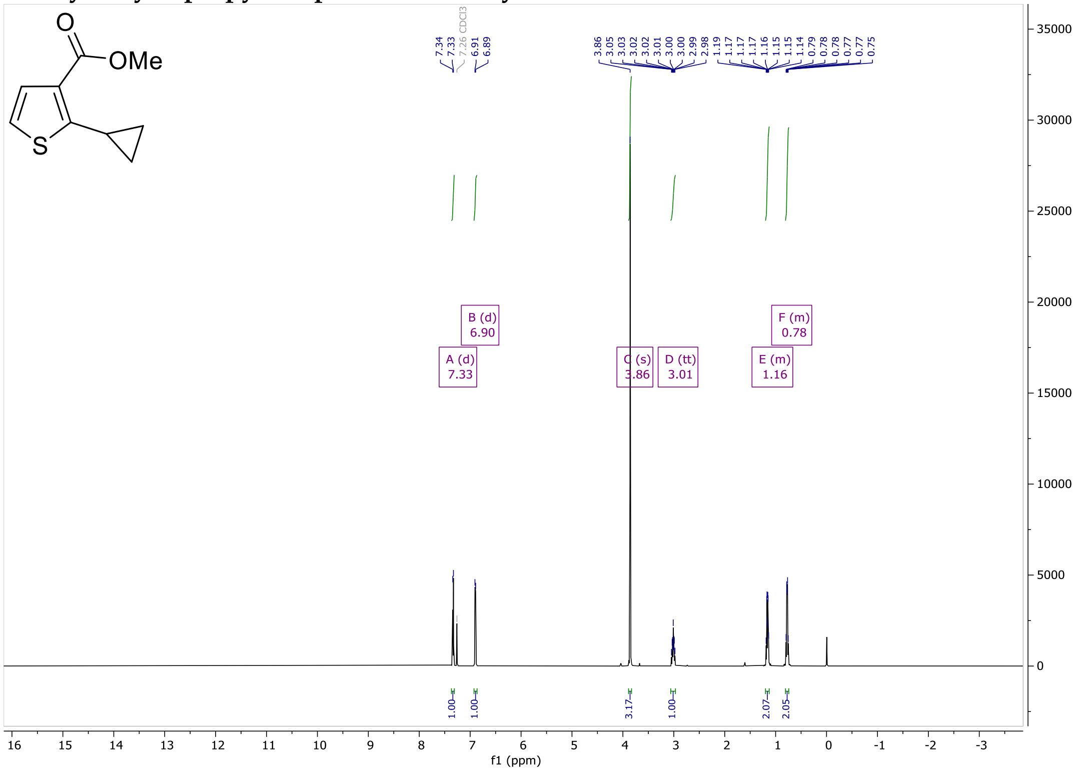

Figure S 13 ^{1}H NMR spectra of compound **7a** in $CDCl_3$ (400 MHz)

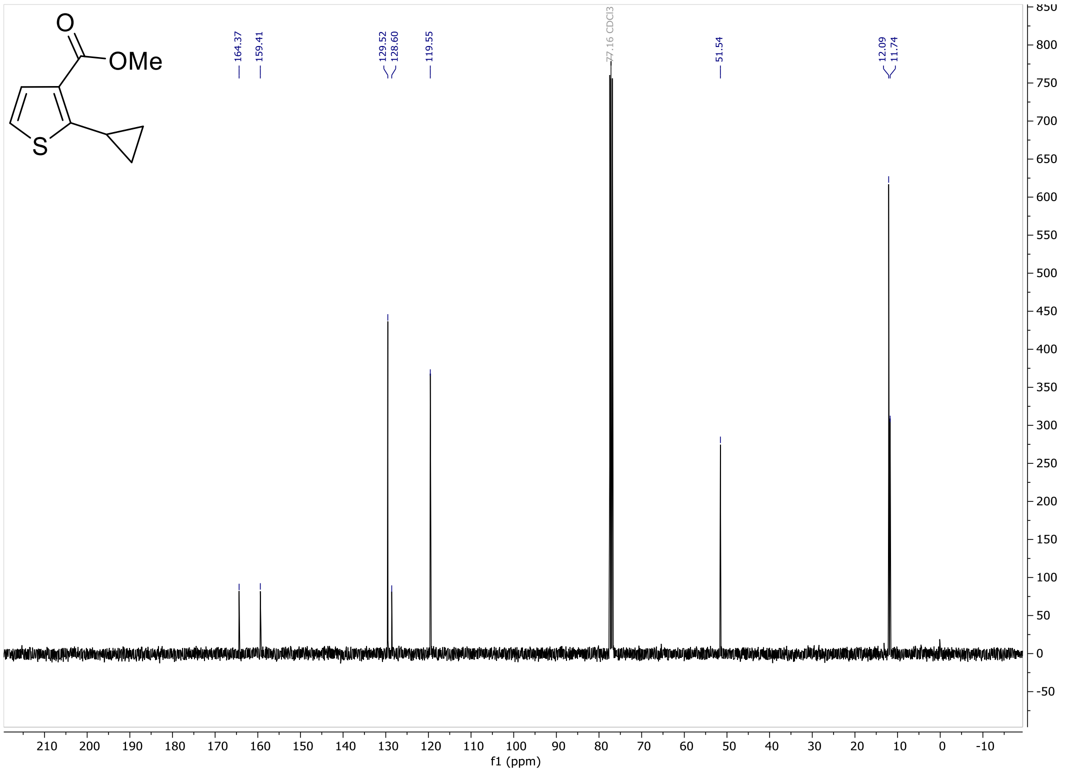

Figure S 14 ^{13}C NMR spectra of compound **7a** in $CDCl_3$ (100 MHz)

1-(4-Cyclopropylthiophen-2-yl)ethan-1-one (8a)

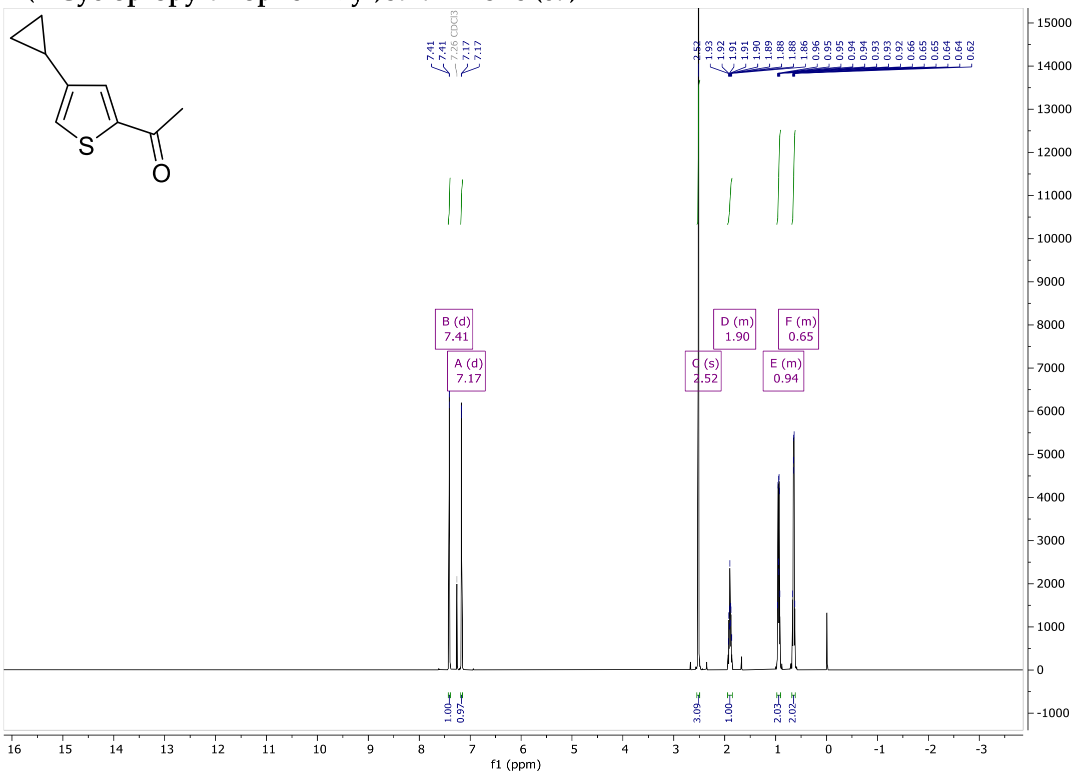

Figure S 15 ^{1}H NMR spectra of compound **8a** in $CDCl_3$ (400 MHz)

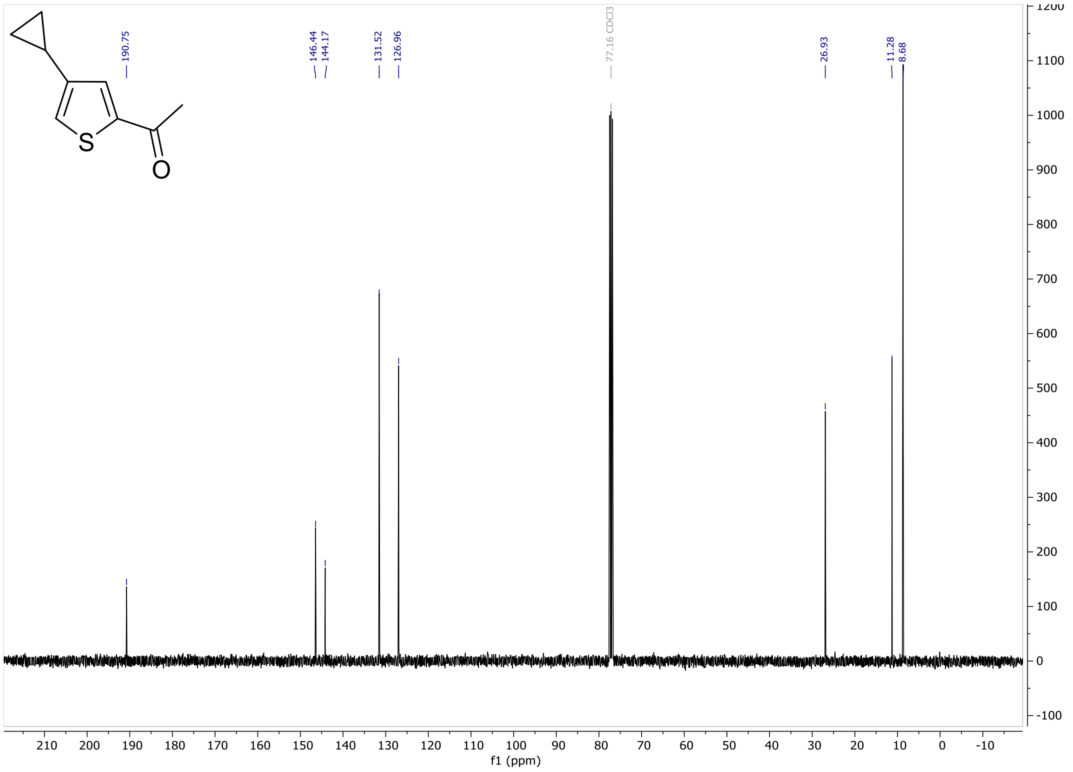

Figure S 16 ^{13}C NMR spectra of compound **8a** in $CDCl_3$ (100 MHz)

4-Cyclopropylthiophene-2-carbaldehyde (9a)

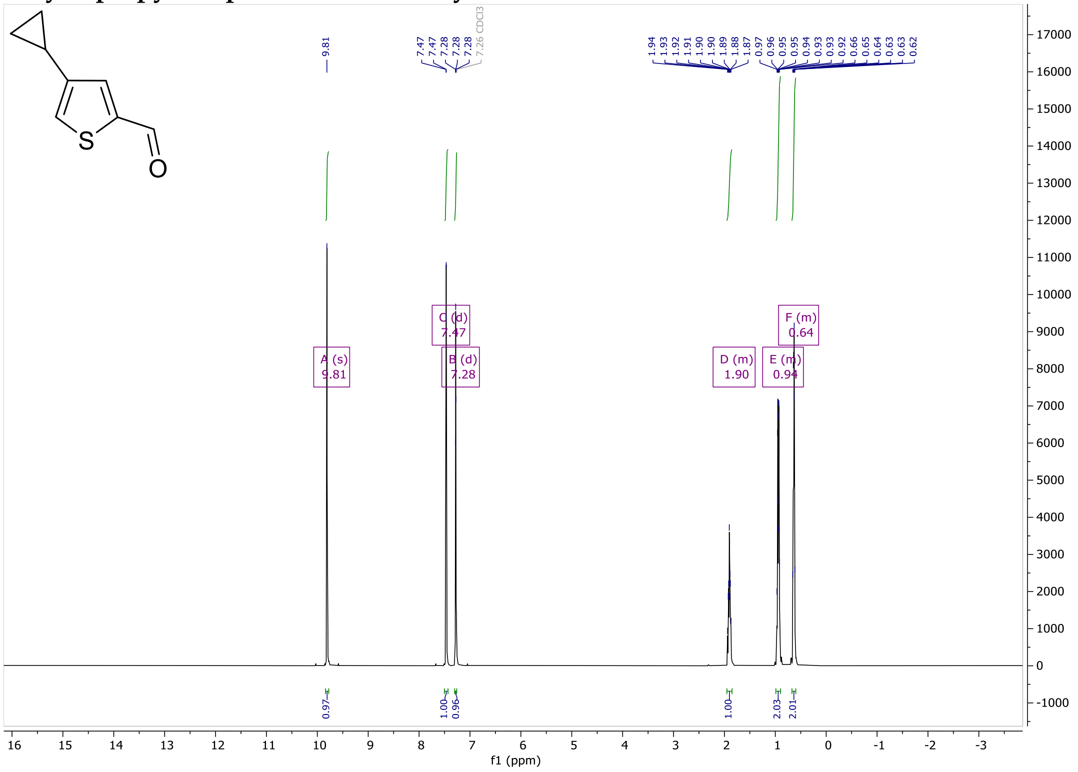

Figure S 17 ^{1}H NMR spectra of compound **9a** in $CDCl_3$ (400 MHz)

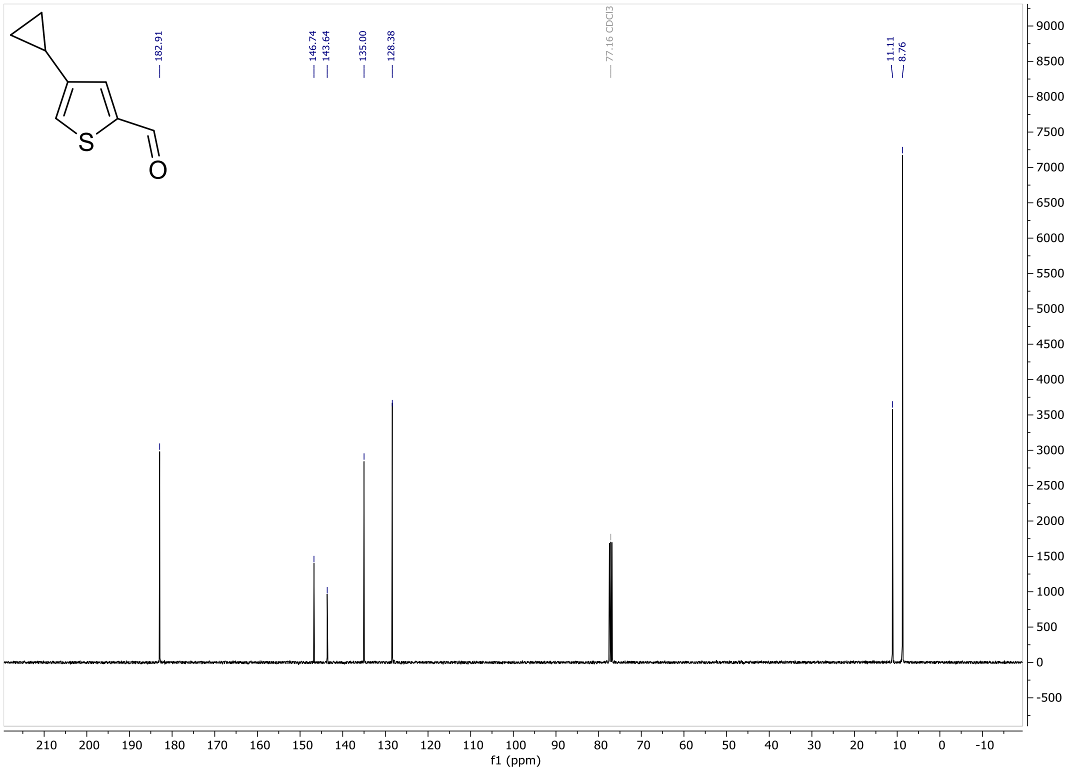

Figure S 18 ^{13}C NMR spectra of compound **9a** in $CDCl_3$ (100 MHz)

2-Cyclopropylthiophene-3-carbaldehyde (10a)

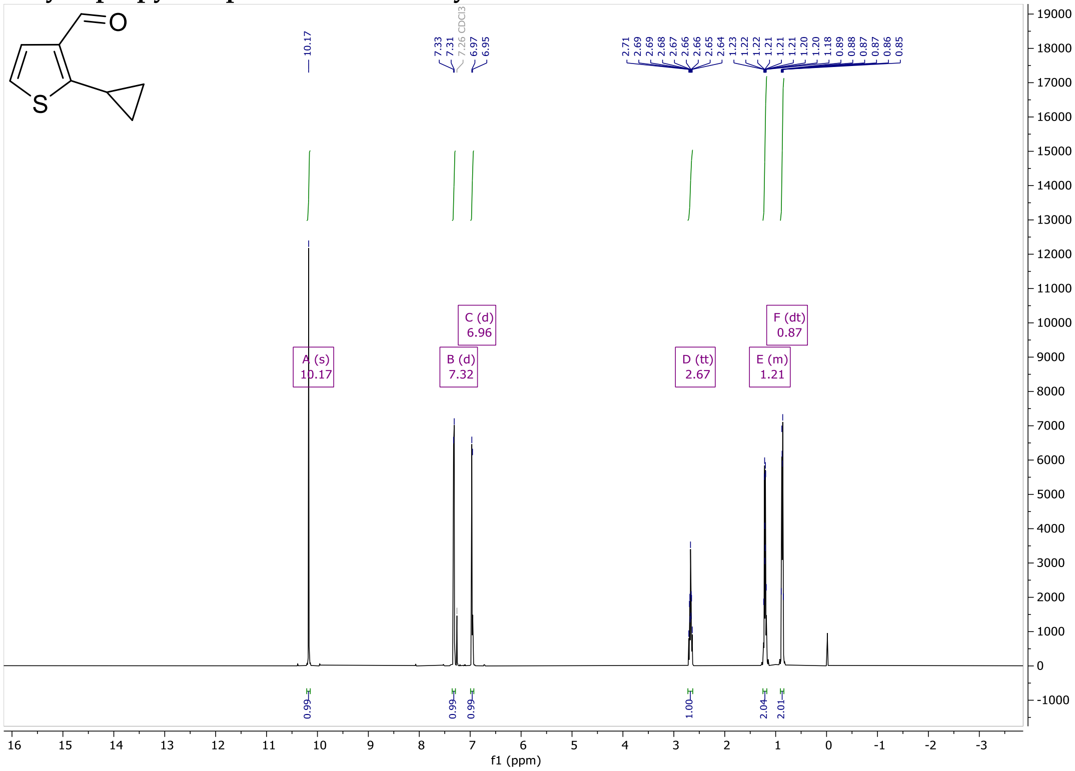

Figure S 19 ^{1}H NMR spectra of compound **10a** in $CDCl_3$ (400 MHz)

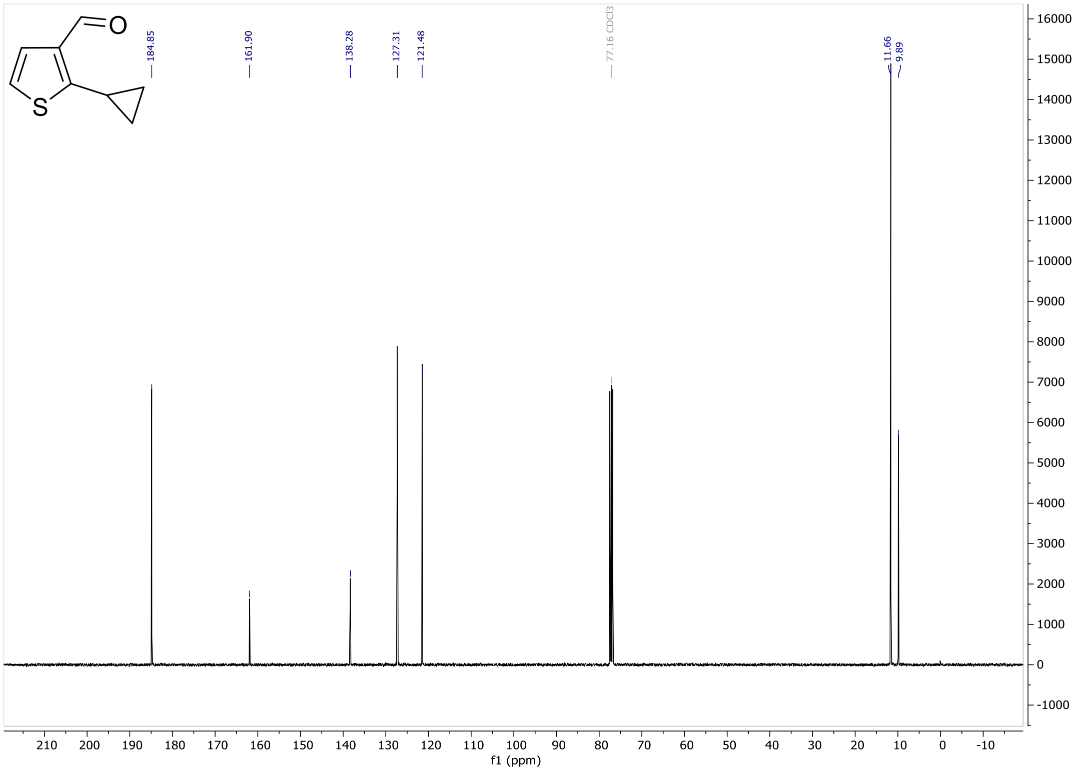

Figure S 20 ^{13}C NMR spectra of compound **10a** in $CDCl_3$ (100 MHz)

5-Cyclopropylthiophene-2-carbaldehyde (11a)

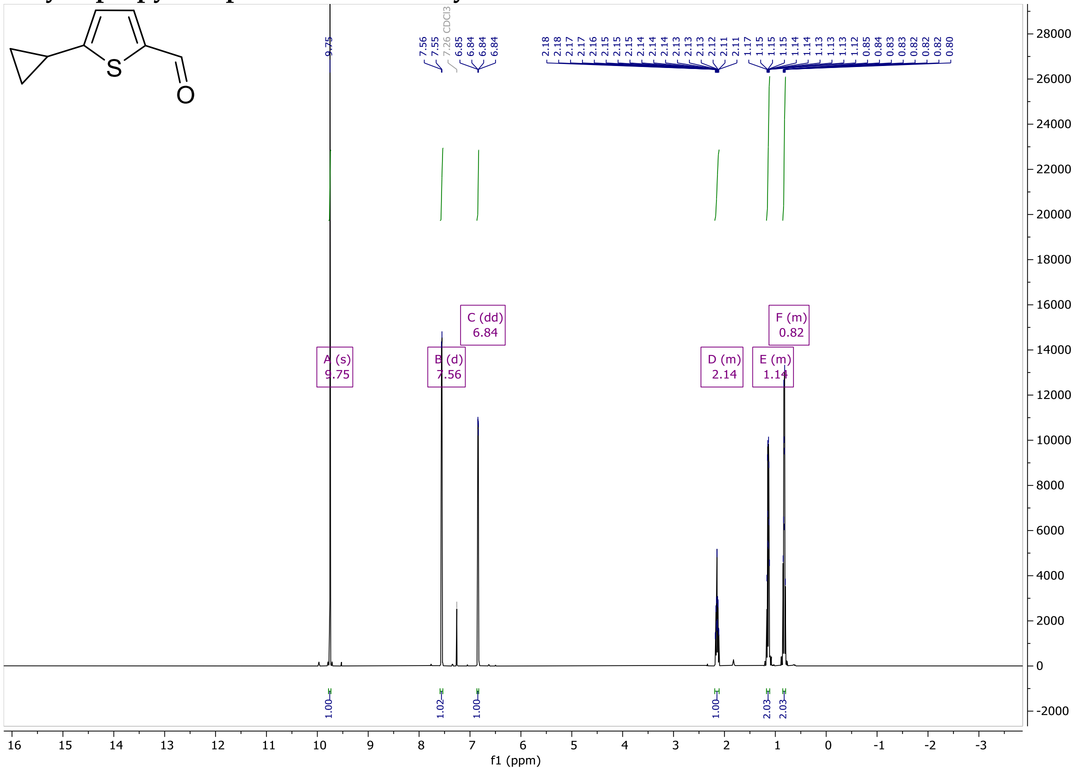

Figure S 21 ^{1}H NMR spectra of compound **11a** in $CDCl_3$ (400 MHz)

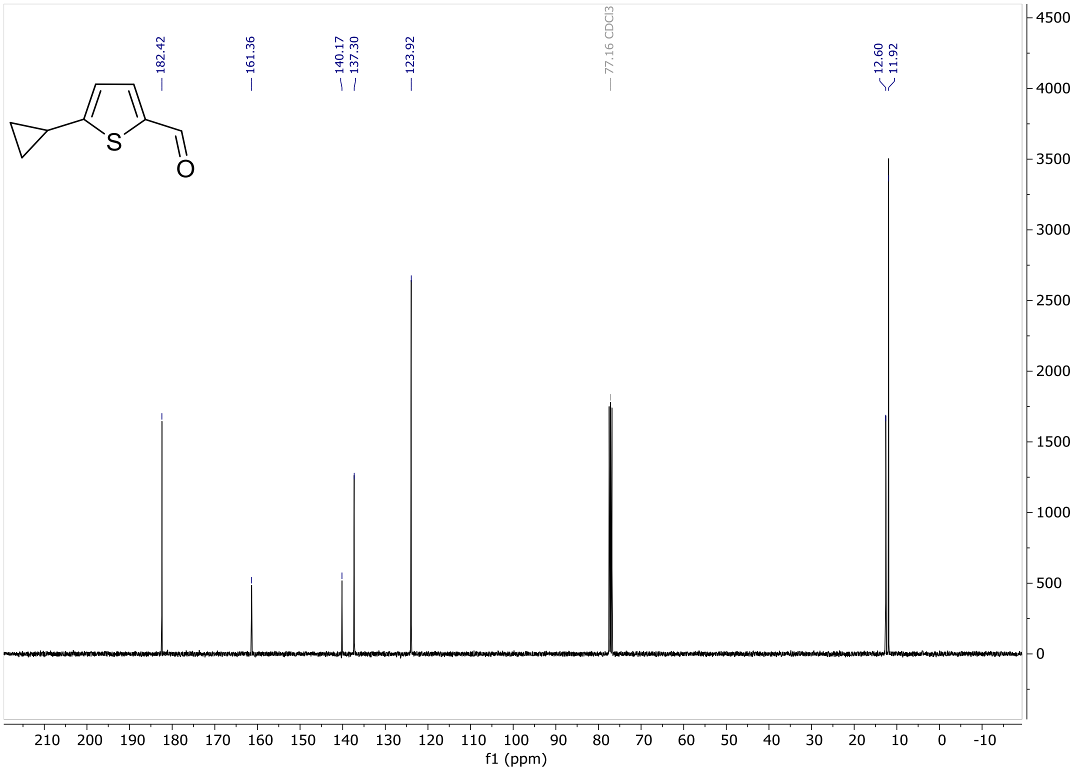

Figure S 22 ^{13}C NMR spectra of compound **11a** in $CDCl_3$ (100 MHz)

3-Cyclopropylthiophene-2-carbaldehyde (12a)

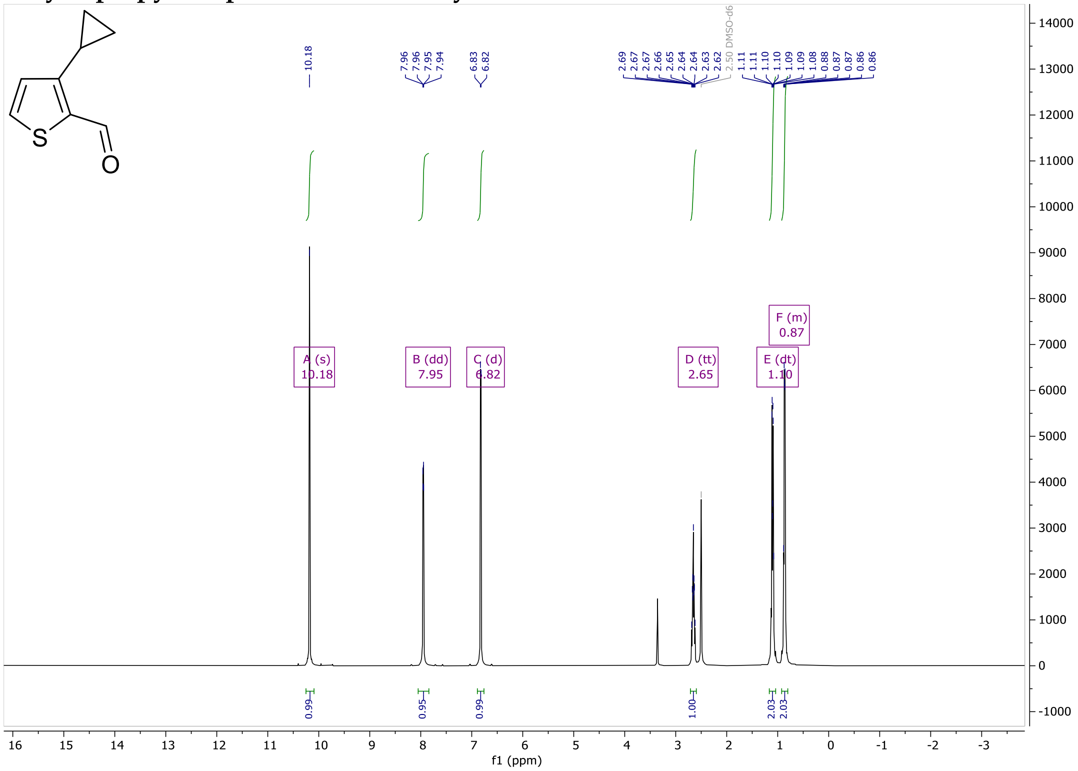

Figure S 23 ^{1}H NMR spectra of compound **12a** in DMSO-d_6 (400 MHz)

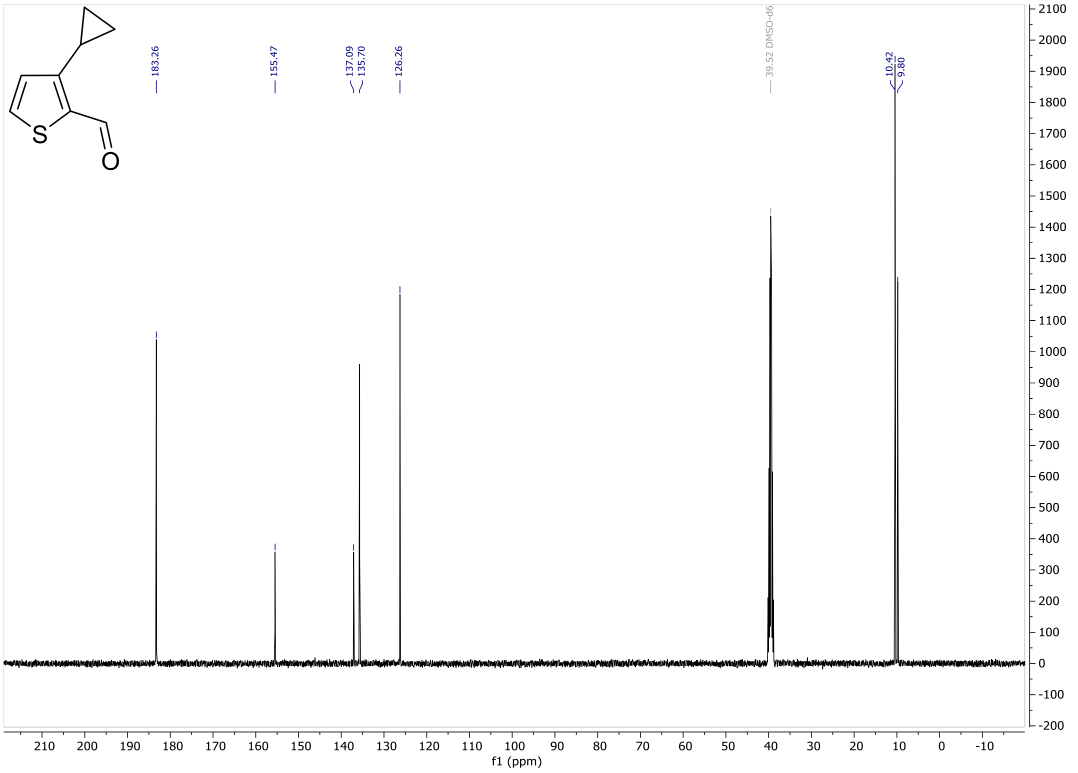

Figure S 24 ^{13}C NMR spectra of compound **12a** in DMSO-d_6 (100 MHz)

4-Cyclopropylthiophene-3-carbaldehyde (13a)

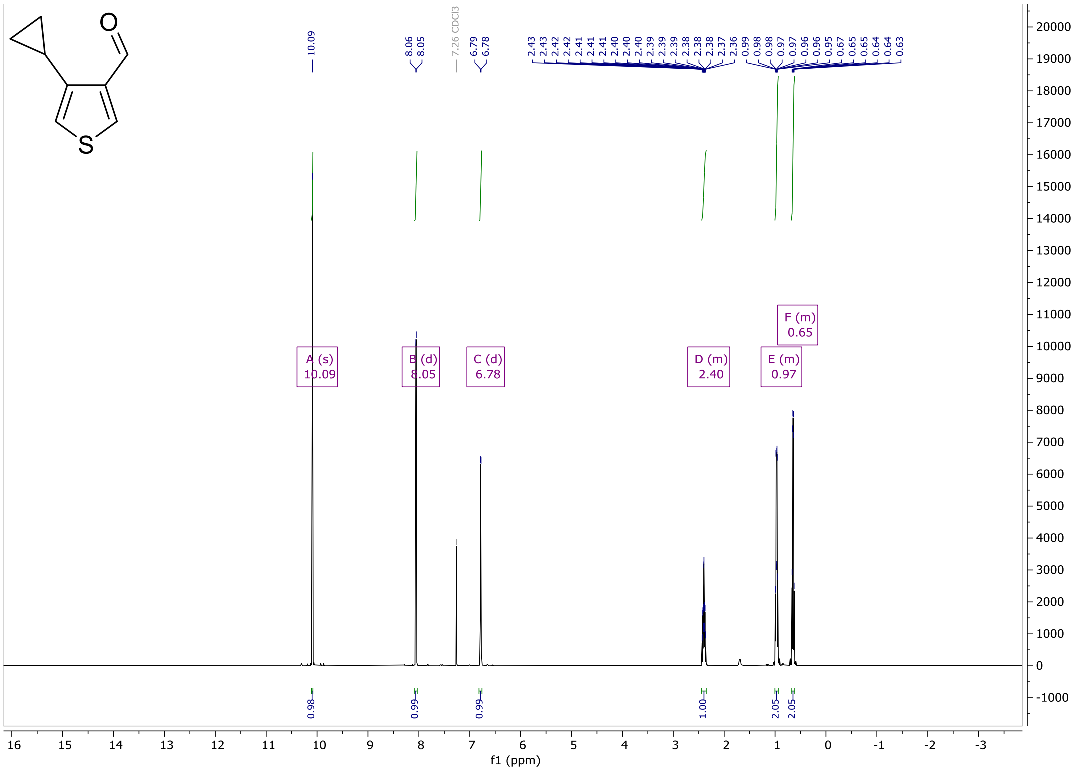

Figure S 25 ^{1}H NMR spectra of compound **13a** in $CDCl_3$ (400 MHz)

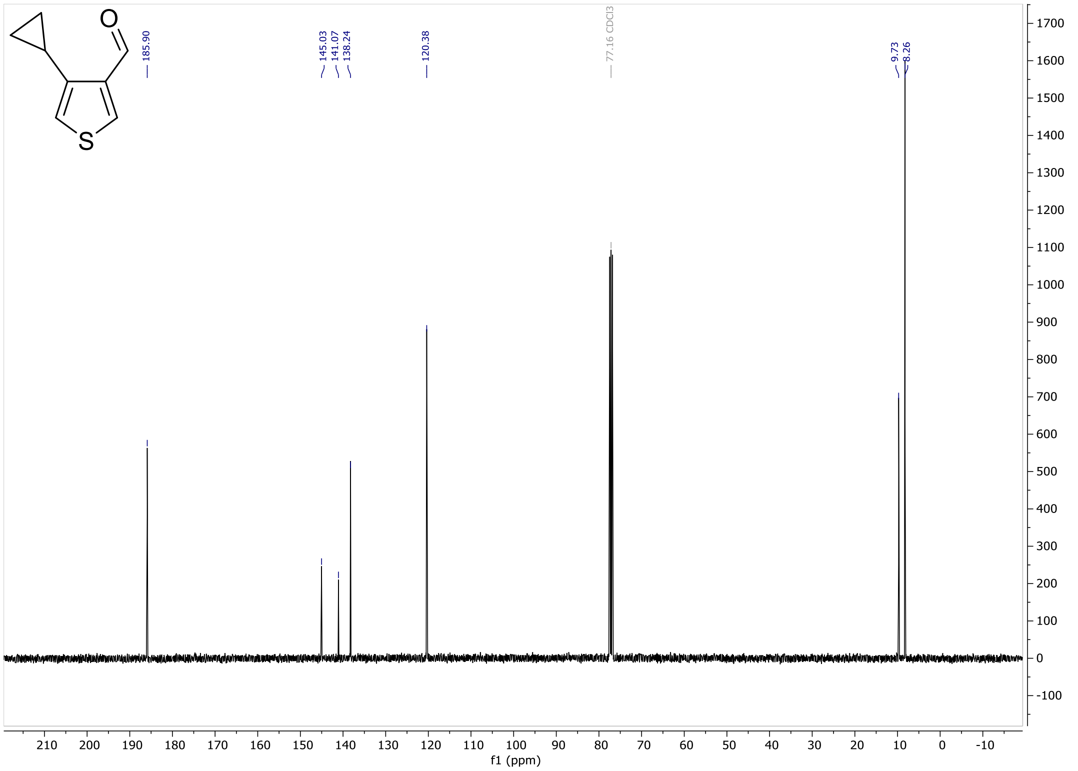

Figure S 26 ^{13}C NMR spectra of compound **13a** in $CDCl_3$ (100 MHz)

4-Cyclopropylthiophene-2-carbonitrile (15a)

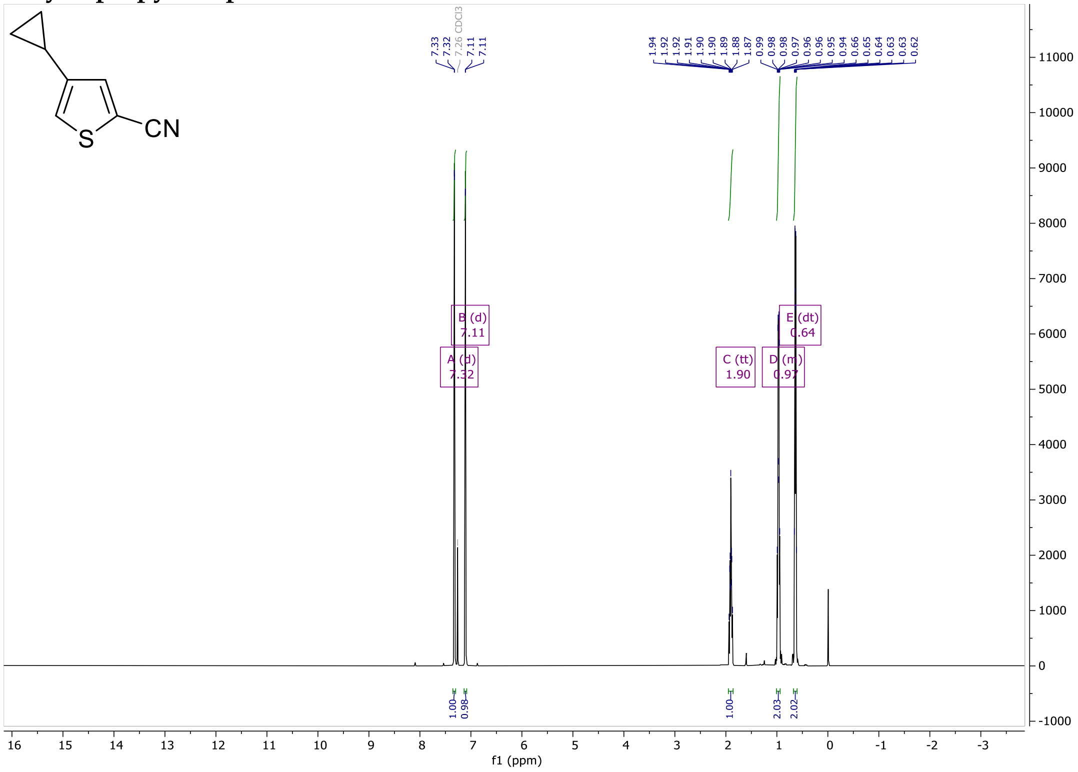

Figure S 27 ^{1}H NMR spectra of compound **15a** in $CDCl_3$ (400 MHz)

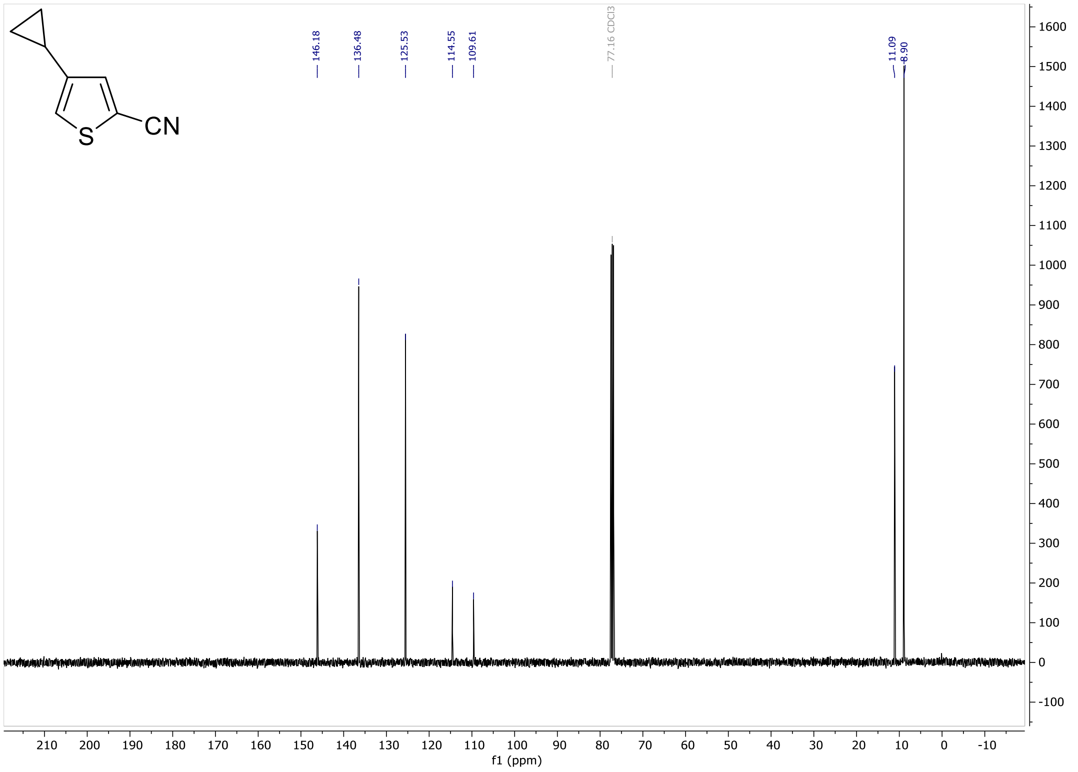

Figure S 28 ^{13}C NMR spectra of compound **15a** in $CDCl_3$ (100 MHz)

2-Cyclopropylthiophene-3-carbonitrile (16a)

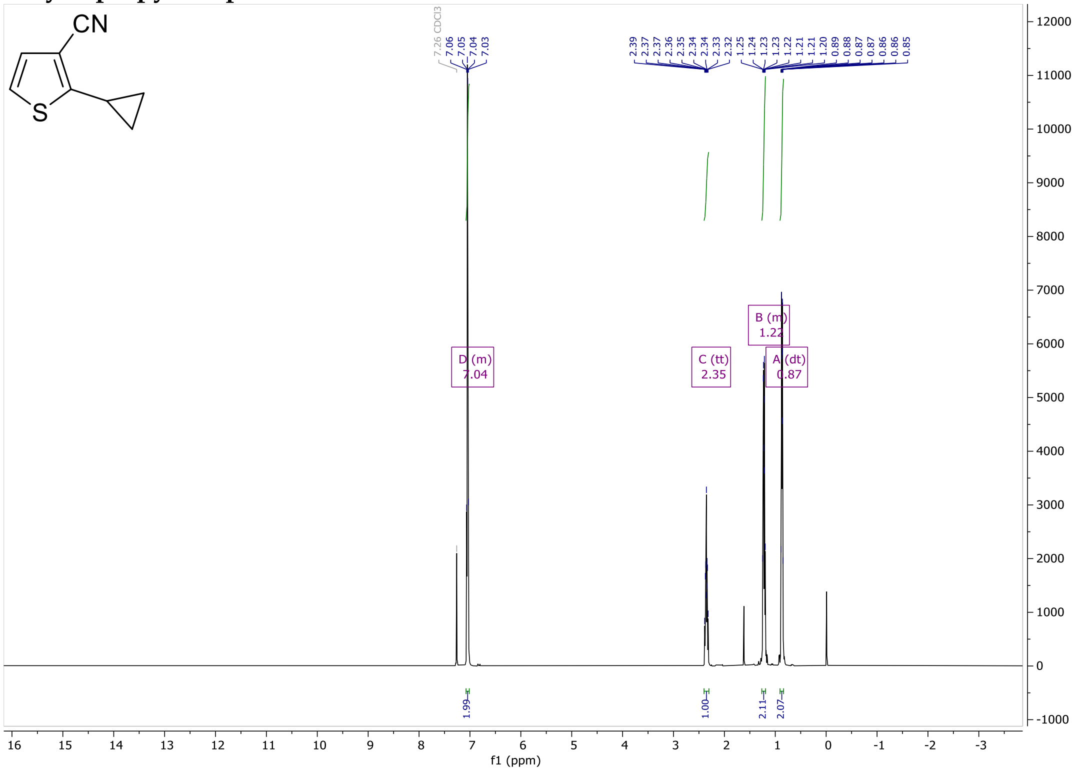

Figure S 29 ^{1}H NMR spectra of compound **16a** in $CDCl_3$ (400 MHz)

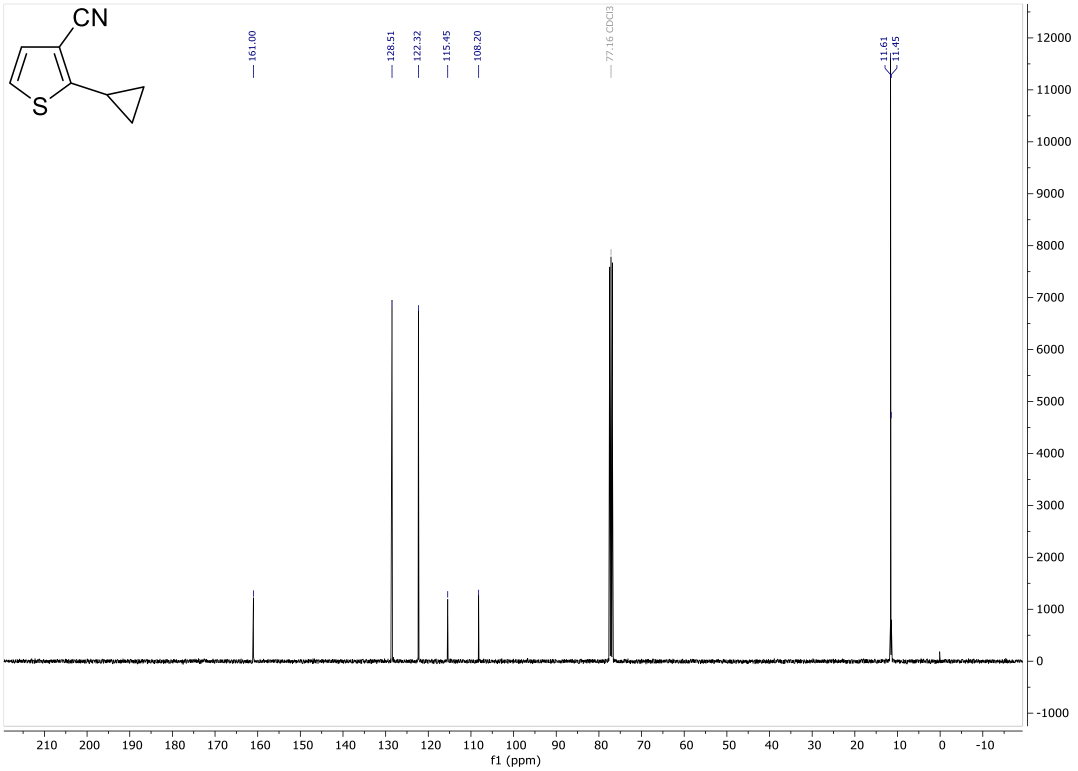

Figure S 30 ^{13}C NMR spectra of compound **16a** in $CDCl_3$ (100 MHz)

5-Cyclopropylthiophene-2-carbonitrile (17a)

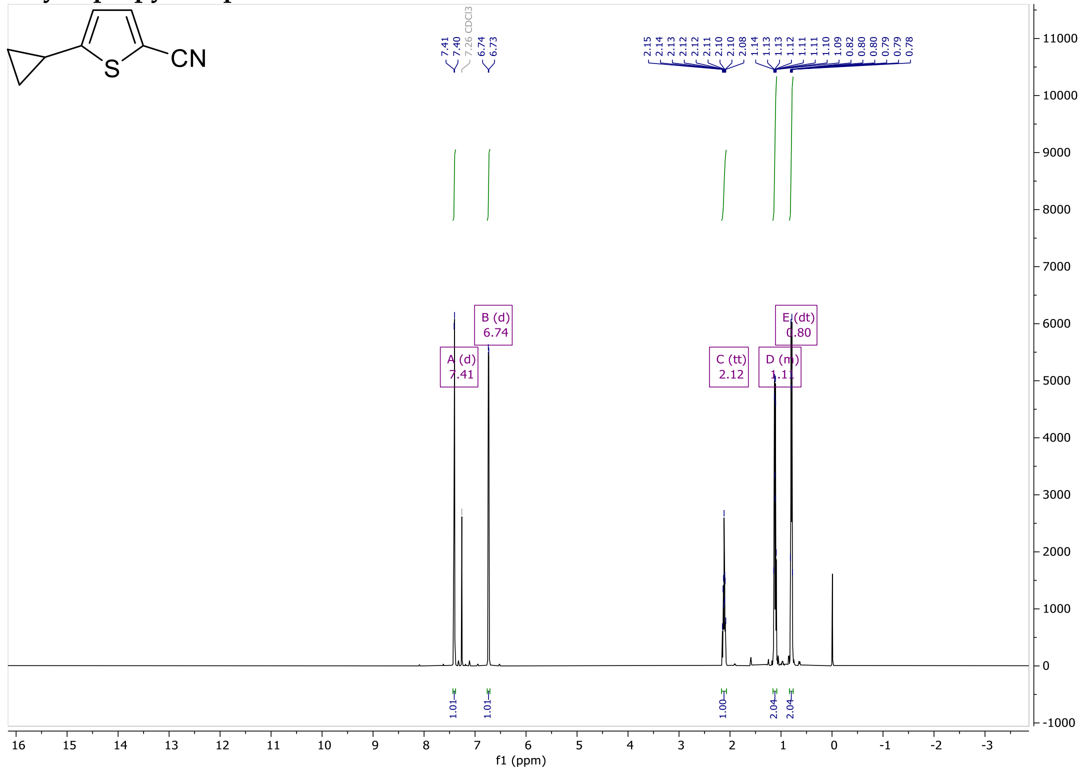

Figure S 31 ^{1}H NMR spectra of compound **17a** in $CDCl_3$ (400 MHz)

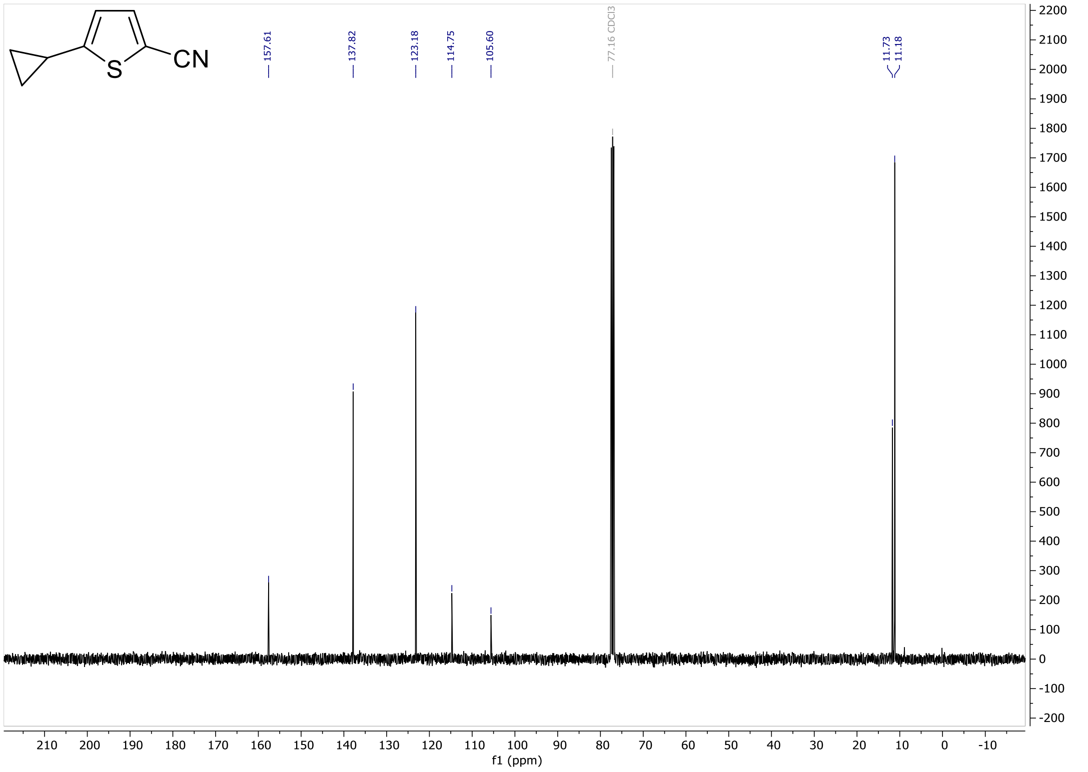

Figure S 32 ^{13}C NMR spectra of compound **17a** in $CDCl_3$ (100 MHz)

3-Cyclopropylthiophene-2-carbonitrile (18a)

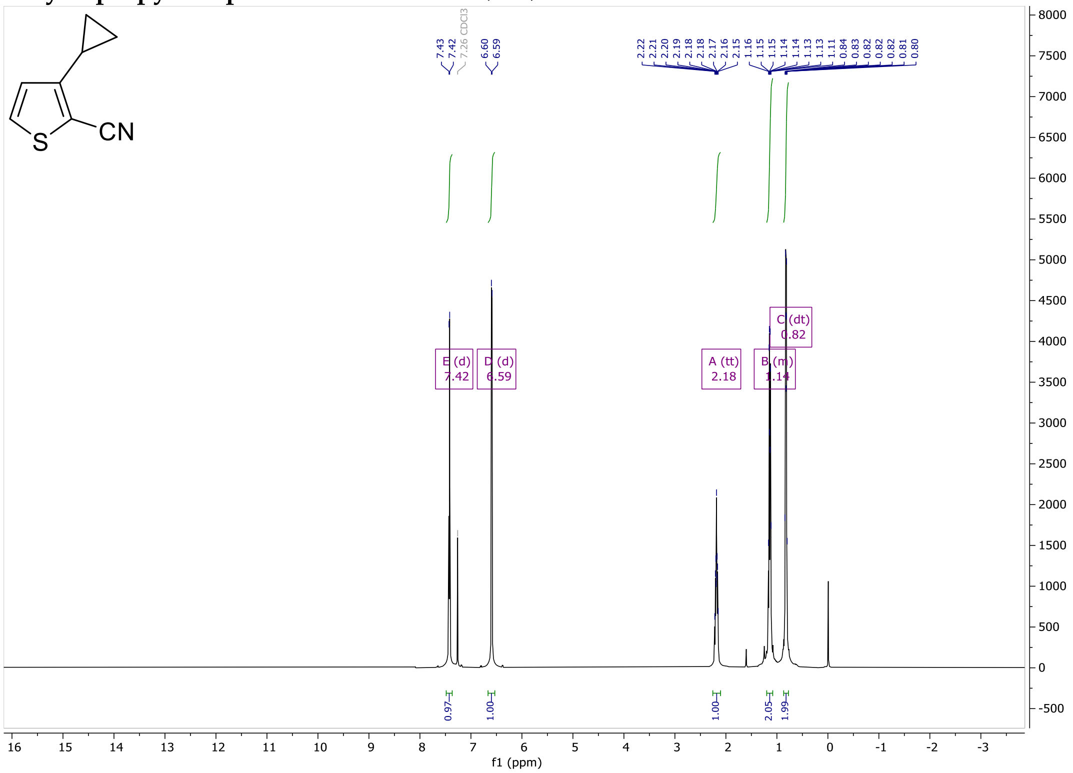

Figure S 33 ^{1}H NMR spectra of compound **18a** in $CDCl_3$ (400 MHz)

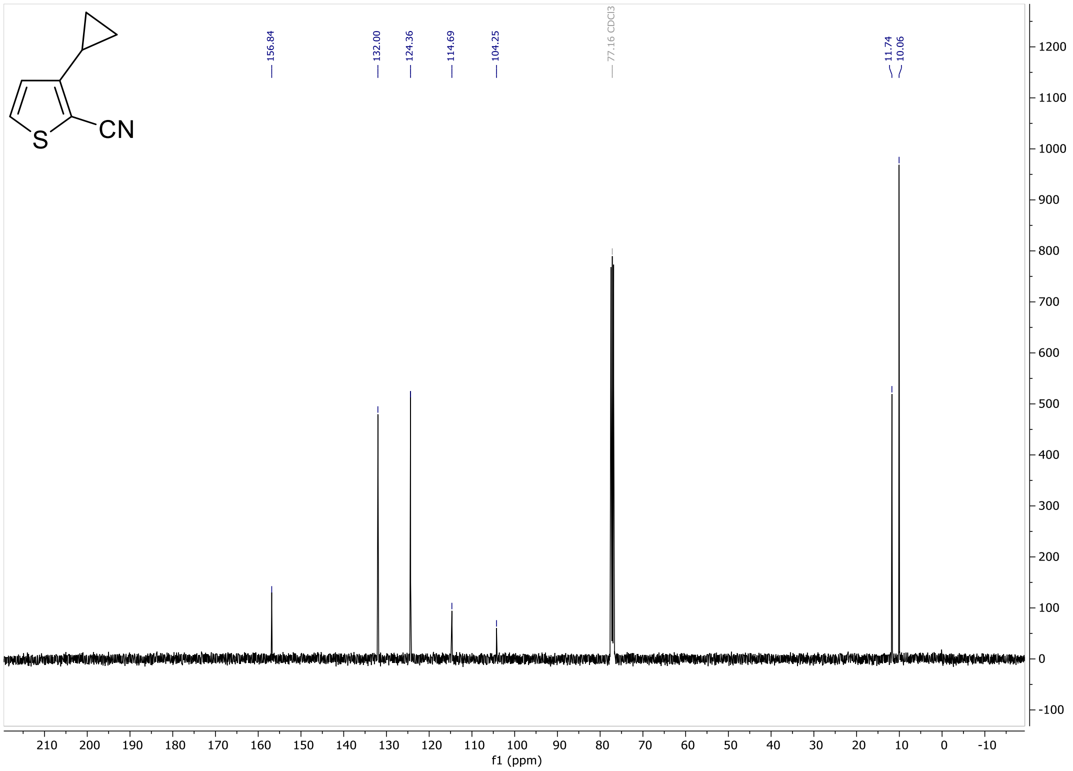

Figure S 34 ^{13}C NMR spectra of compound **18a** in $CDCl_3$ (100 MHz)

4-Cyclopropylthiophene-3-carbonitrile (19a)

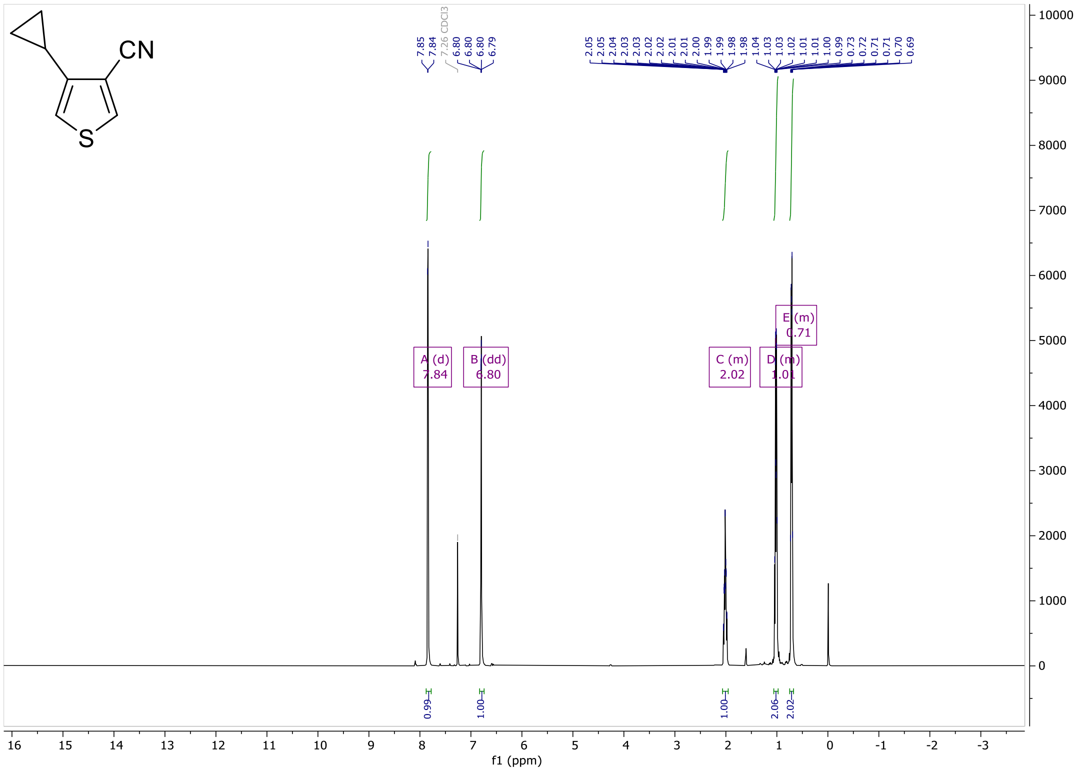

Figure S 35 ^{1}H NMR spectra of compound **19a** in $CDCl_3$ (400 MHz)

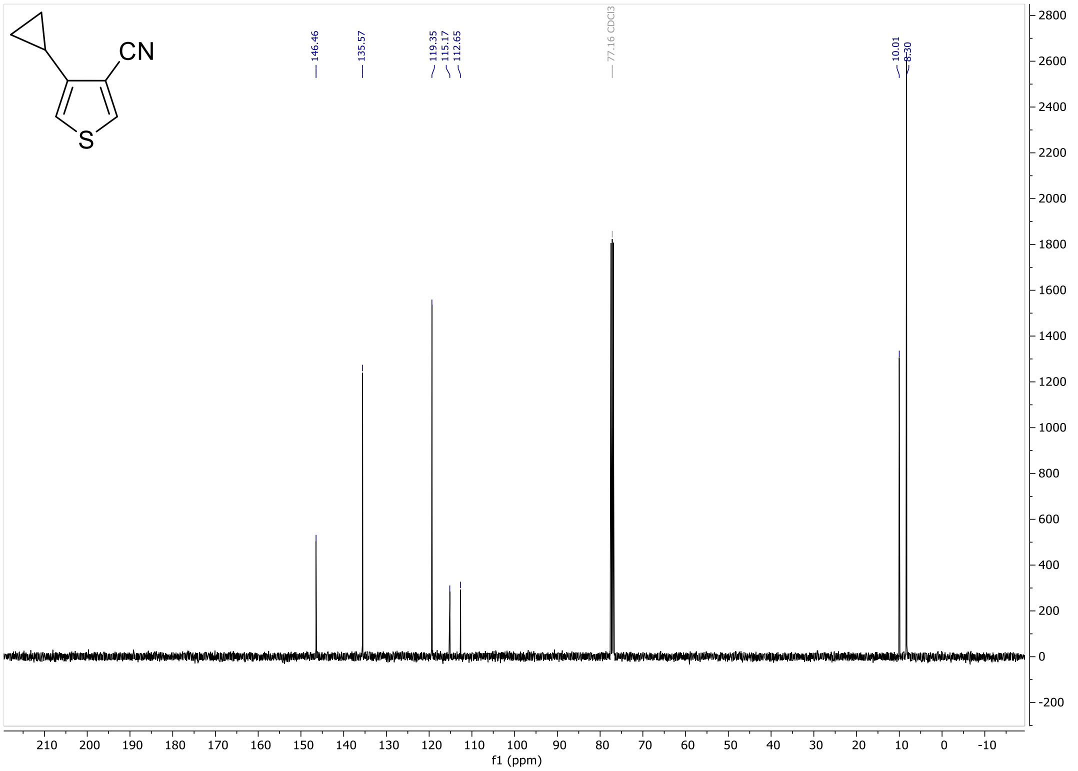

Figure S 36 ^{13}C NMR spectra of compound **19a** in $CDCl_3$ (100 MHz)

5-Bromo-4-cyclopropylthiophene-2-carbonitrile (15b)

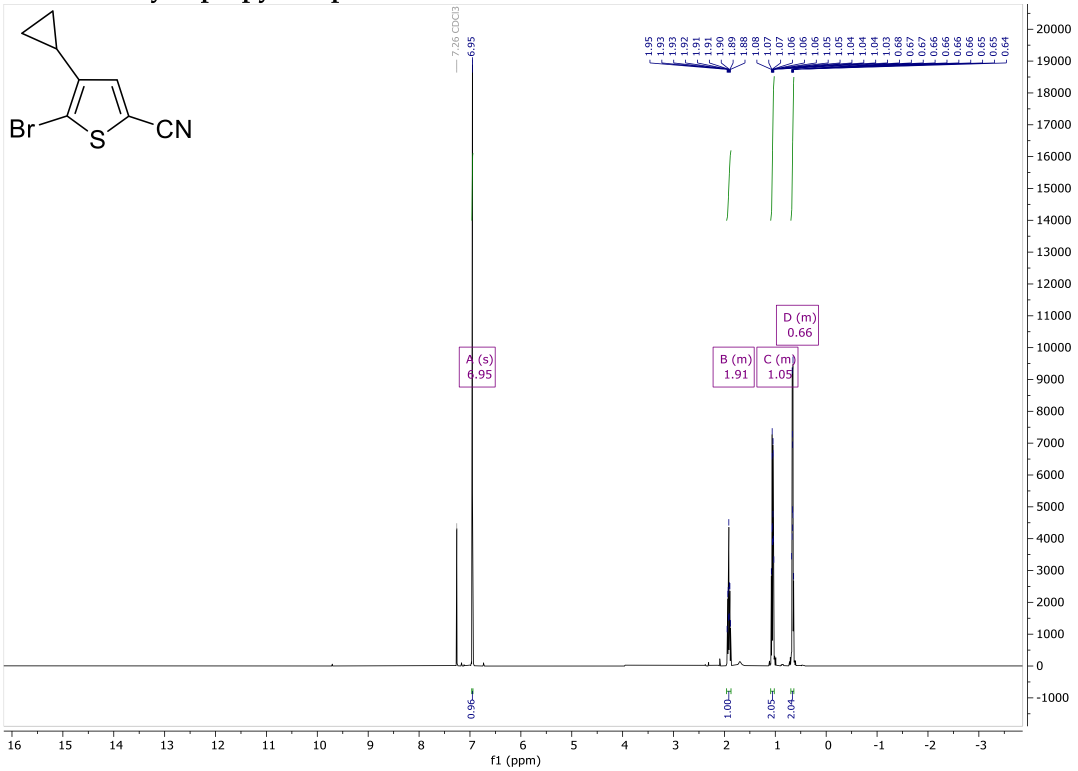

Figure S 37 ¹H NMR spectra of compound **15b** in $CDCl_3$ (400 MHz)

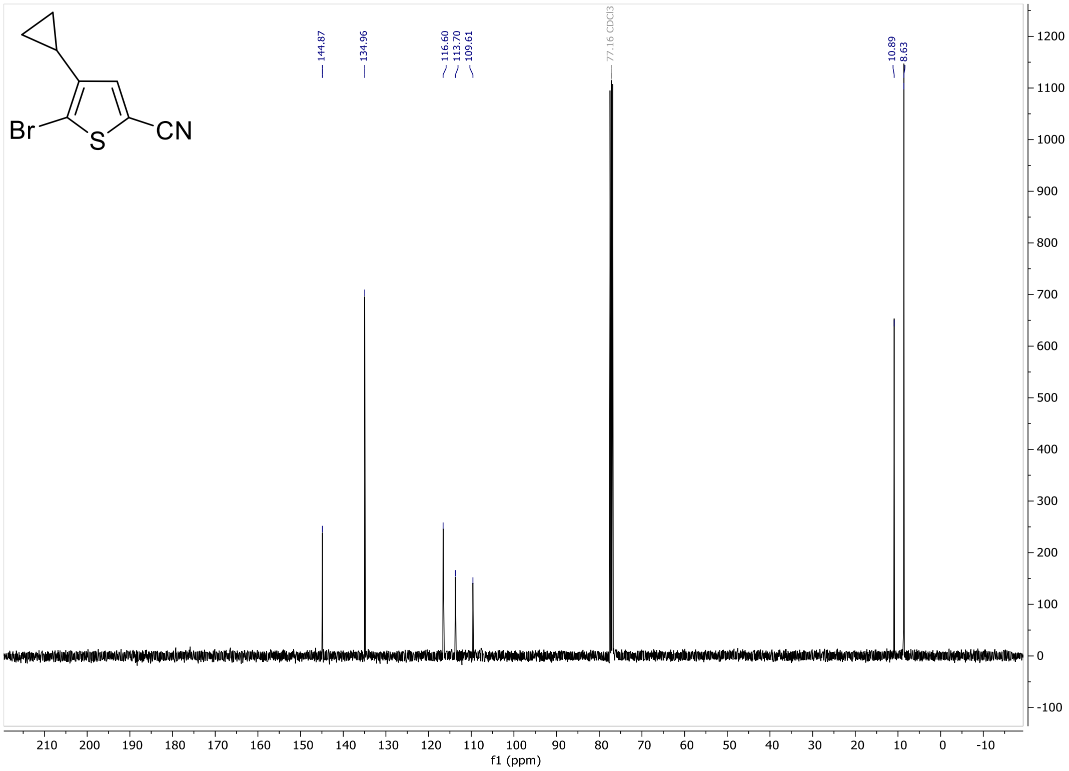

Figure S 38 ¹³C NMR spectra of compound **15b** in $CDCl_3$ (100 MHz)

5-Cyclopropylthiophene-3-carboxylic acid (20a)

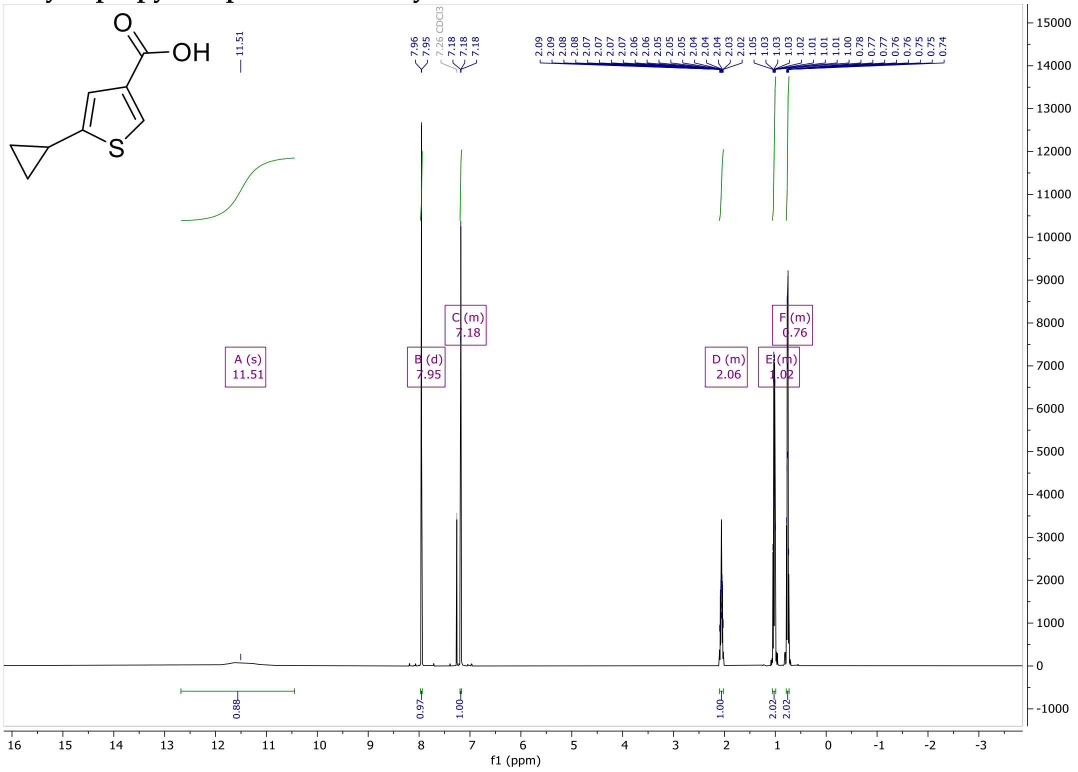

Figure S 39 ¹H NMR spectra of compound **20a** in $CDCl_3$ (400 MHz)

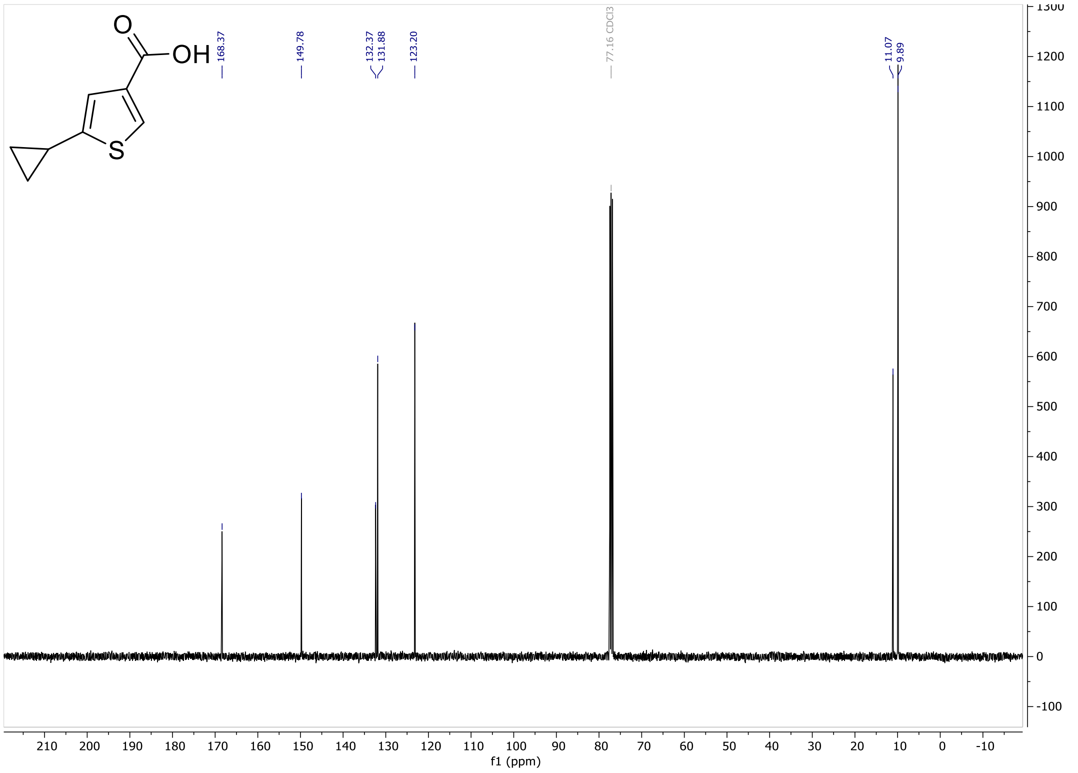

Figure S 40 ¹³C NMR spectra of compound **20a** in $CDCl_3$ (100 MHz)

3-Cyclopropylthiophene-2-carboxylic acid (21a)

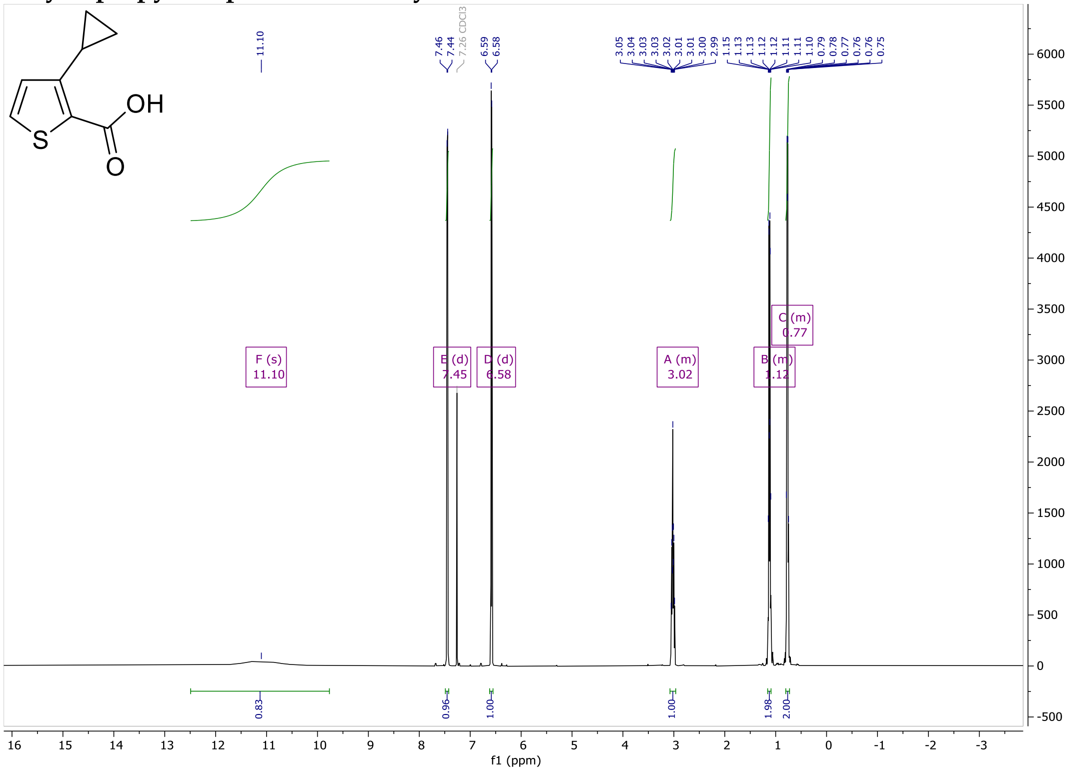

Figure S 41 ^{1}H NMR spectra of compound **21a** in $CDCl_3$ (400 MHz)

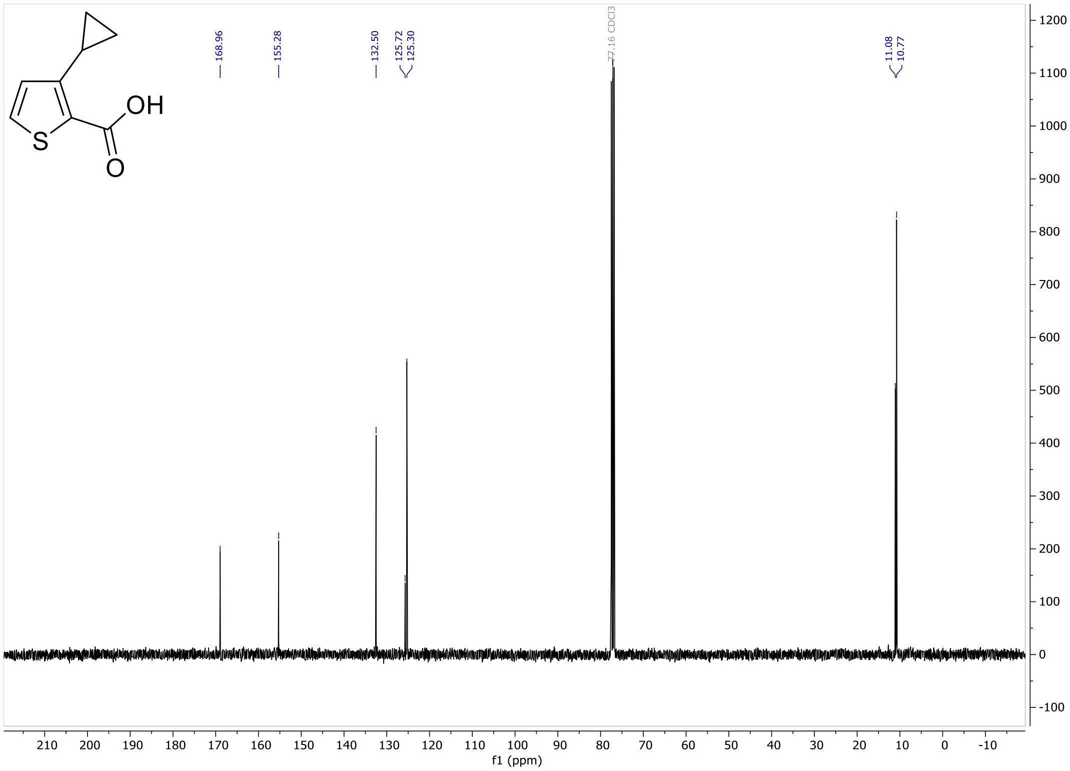

Figure S 42 ^{13}C NMR spectra of compound **21a** in $CDCl_3$ (100 MHz)

4-Cyclopropylthiophene-2-carboxylic acid (22a)

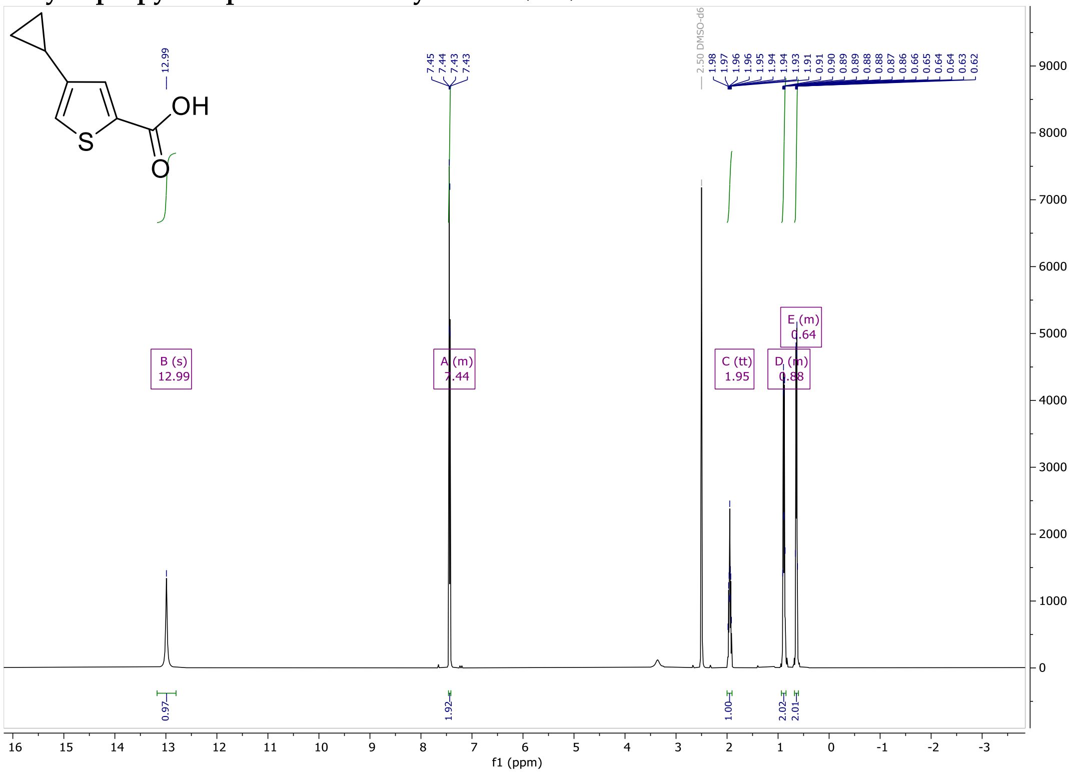

Figure S 43 ^{1}H NMR spectra of compound **22a** in DMSO-d_6 (400 MHz)

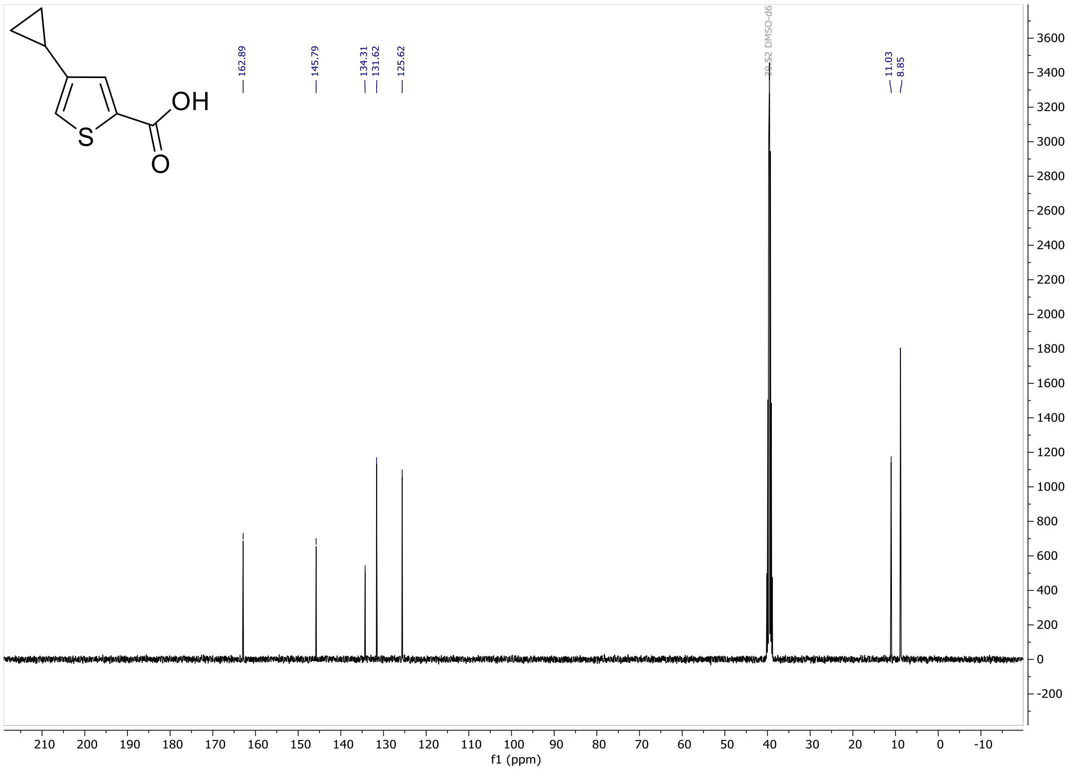

Figure S 44 ^{13}C NMR spectra of compound **22a** in DMSO-d_6 (100 MHz)

2-Bromo-5-cyclopropylthiophene (1b)

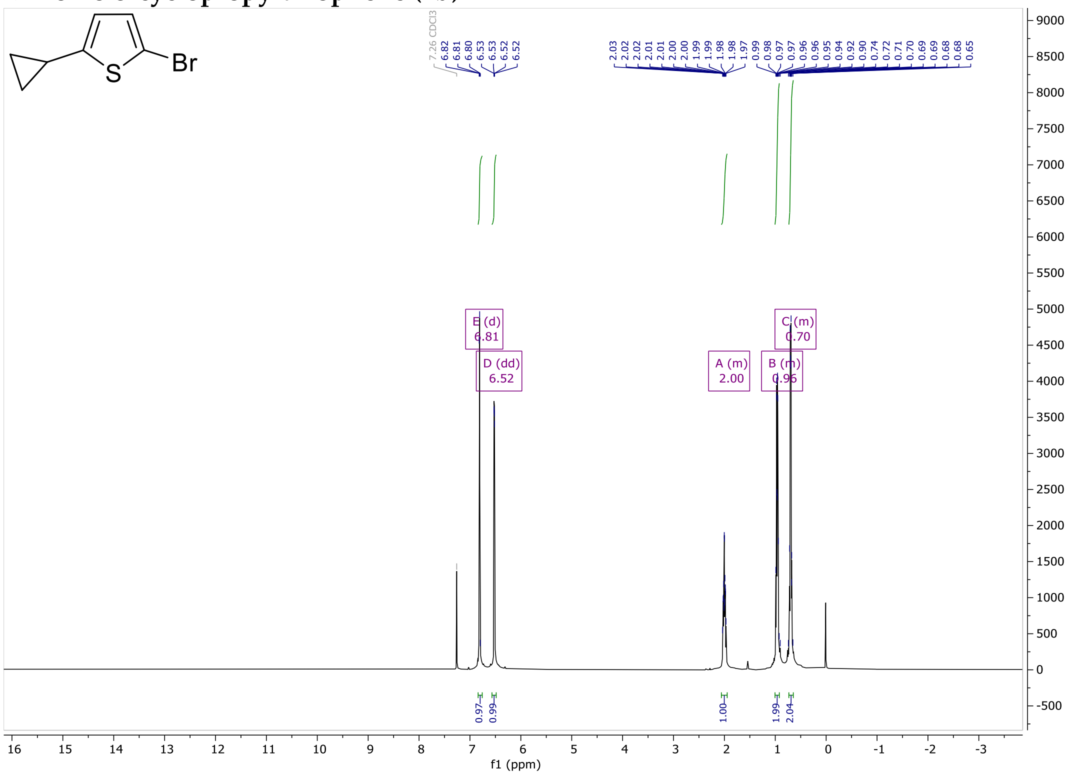

Figure S 45 ^{1}H NMR spectra of compound **1b** in $CDCl_3$ (400 MHz)

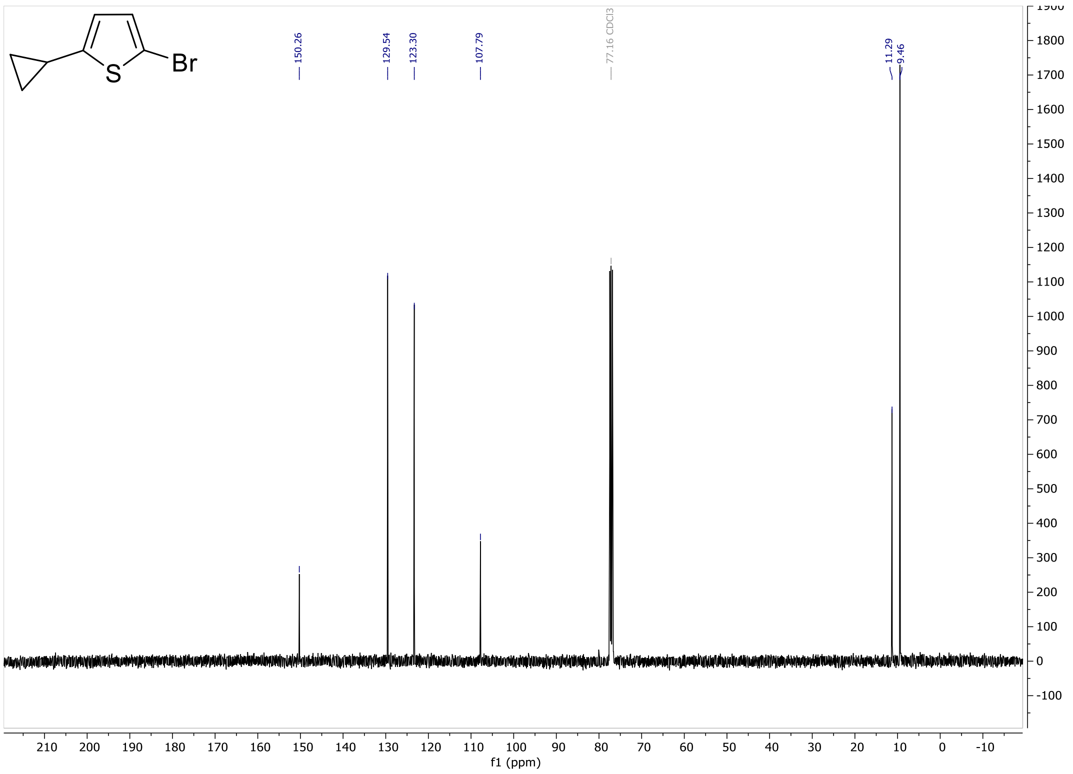

Figure S 46 ^{13}C NMR spectra of compound **1b** in $CDCl_3$ (100 MHz)

2-Bromo-3-cyclopropylthiophene (2b)

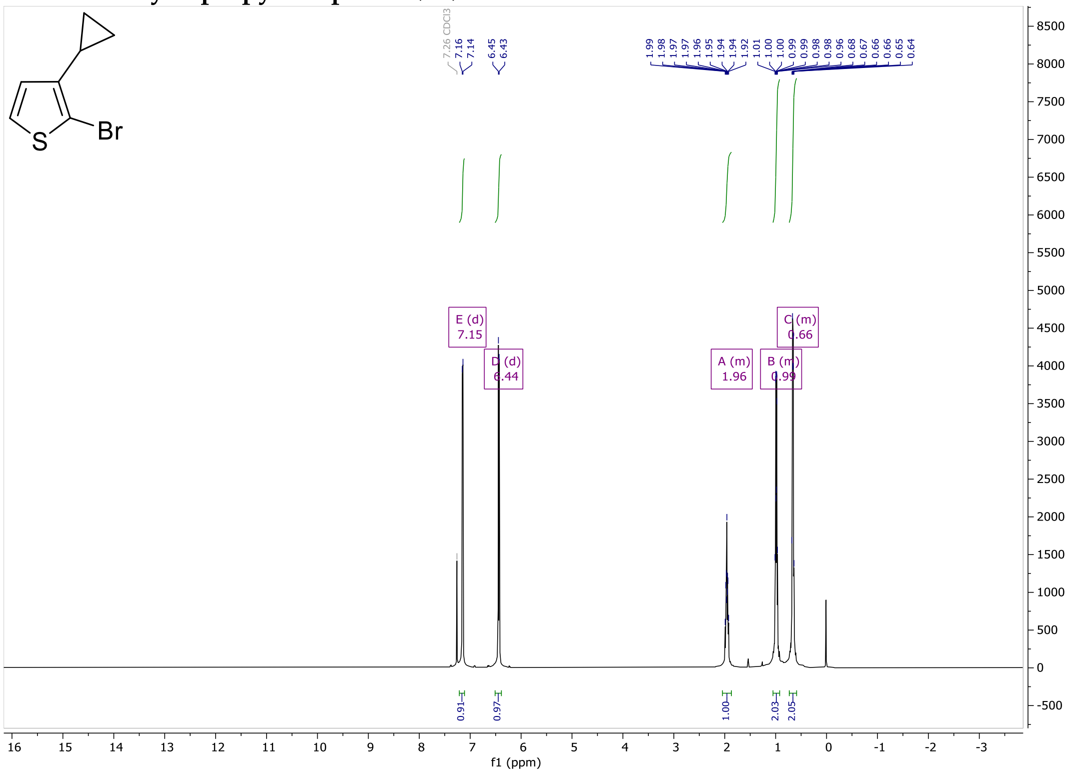

Figure S 47 ^{1}H NMR spectra of compound **2b** in $CDCl_3$ (400 MHz)

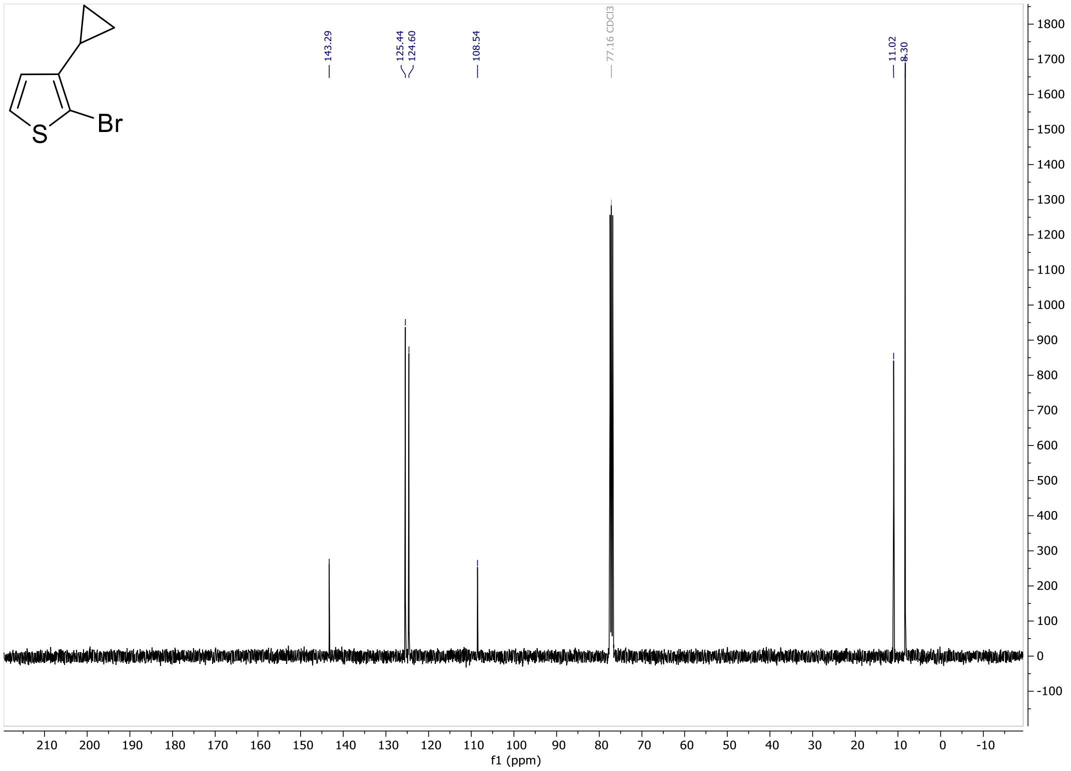

Figure S 48 ^{13}C NMR spectra of compound **2b** in $CDCl_3$ (100 MHz)

Methyl 5-bromo-4-cyclopropylthiophene-2-carboxylate (6b)

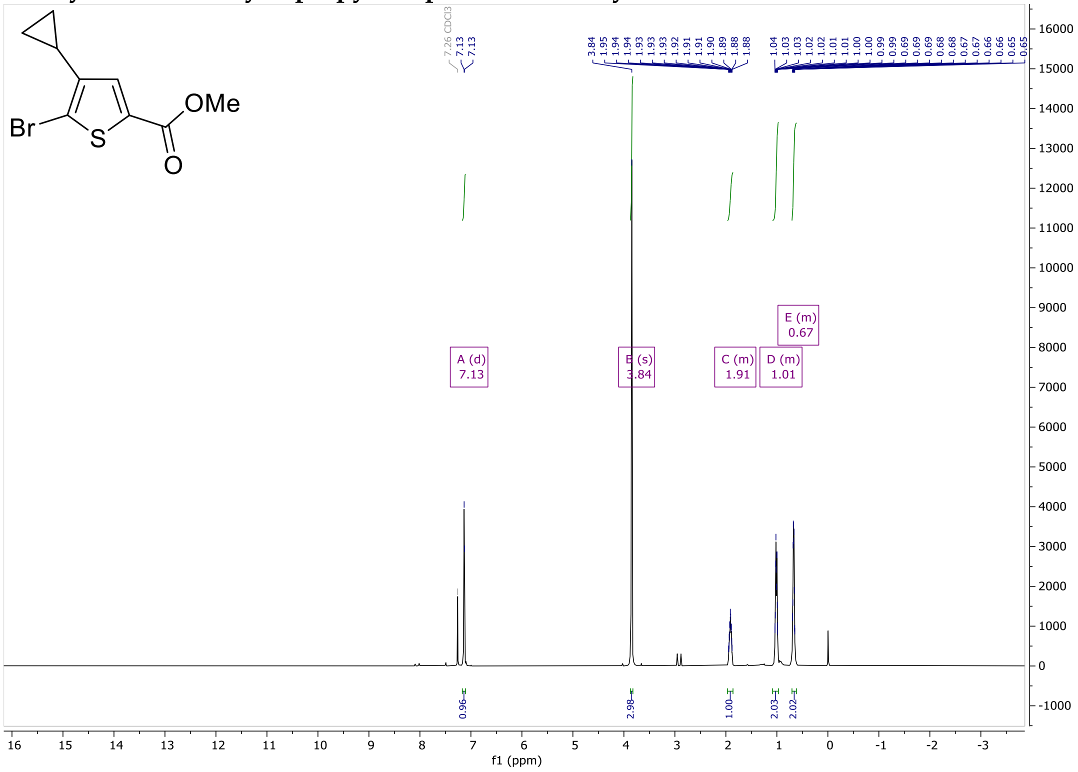

Figure S 49 ^{1}H NMR spectra of compound **6b** in $CDCl_3$ (400 MHz)

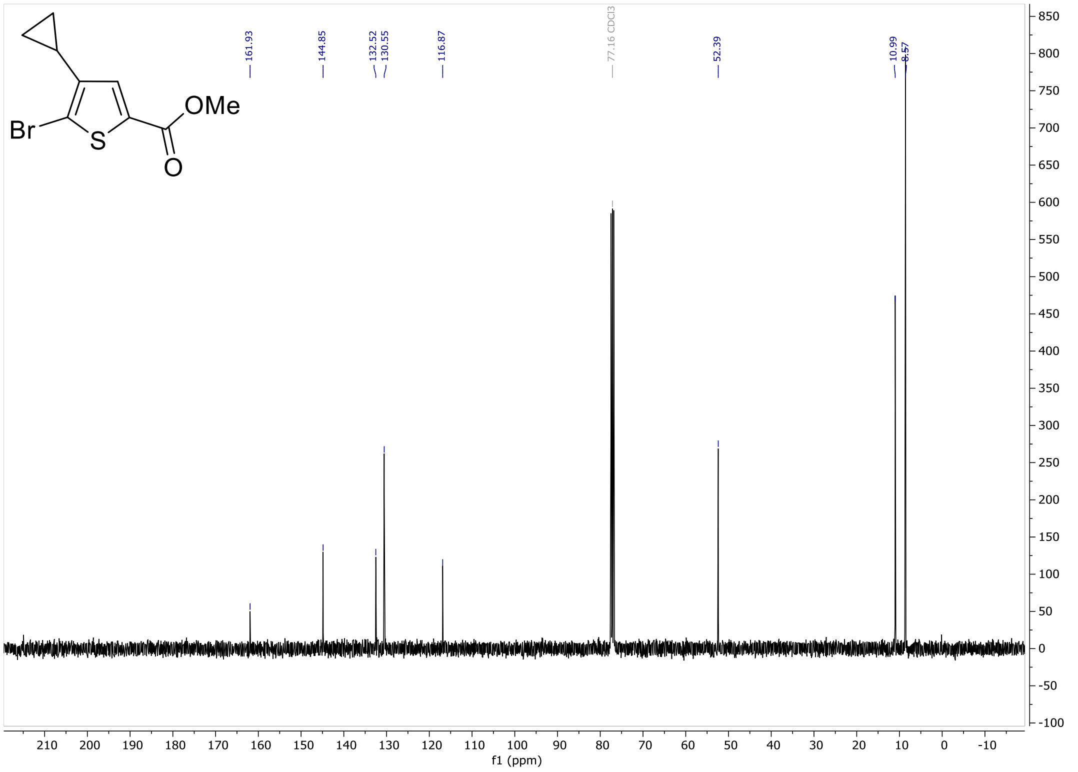

Figure S 50 ^{13}C NMR spectra of compound **6b** in $CDCl_3$ (100 MHz)

Methyl 5-bromo-2-cyclopropylthiophene-3-carboxylate (7b)

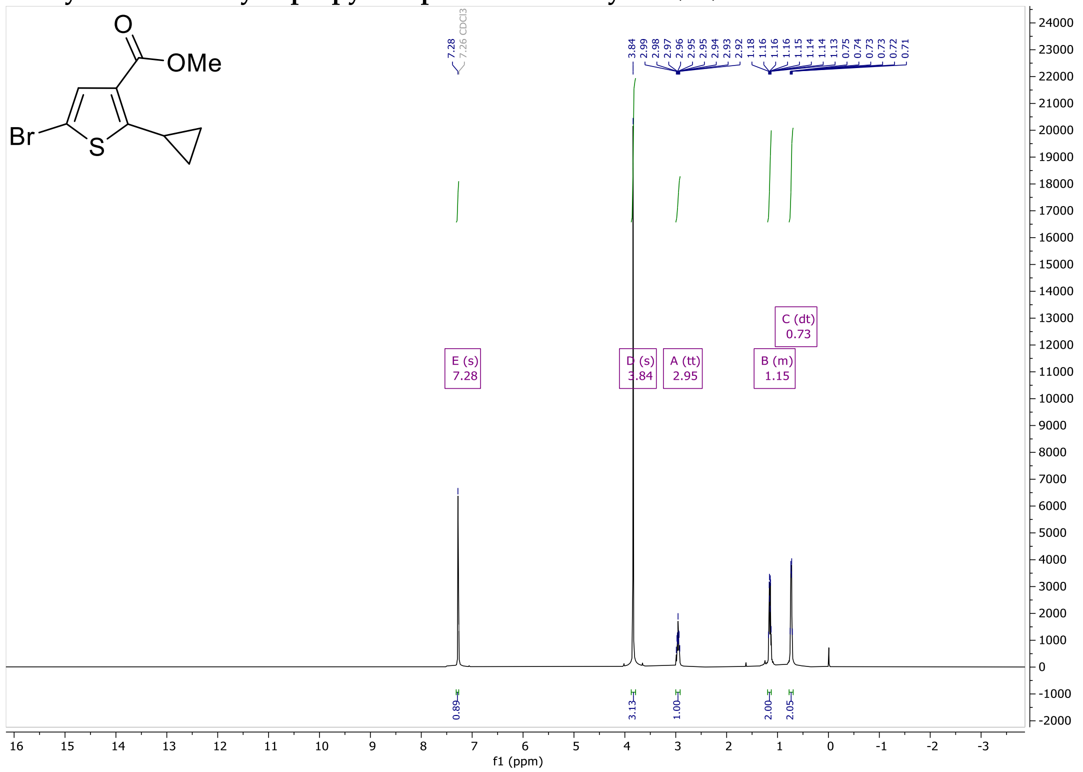

Figure S 51 ^{1}H NMR spectra of compound **7b** in $CDCl_3$ (400 MHz)

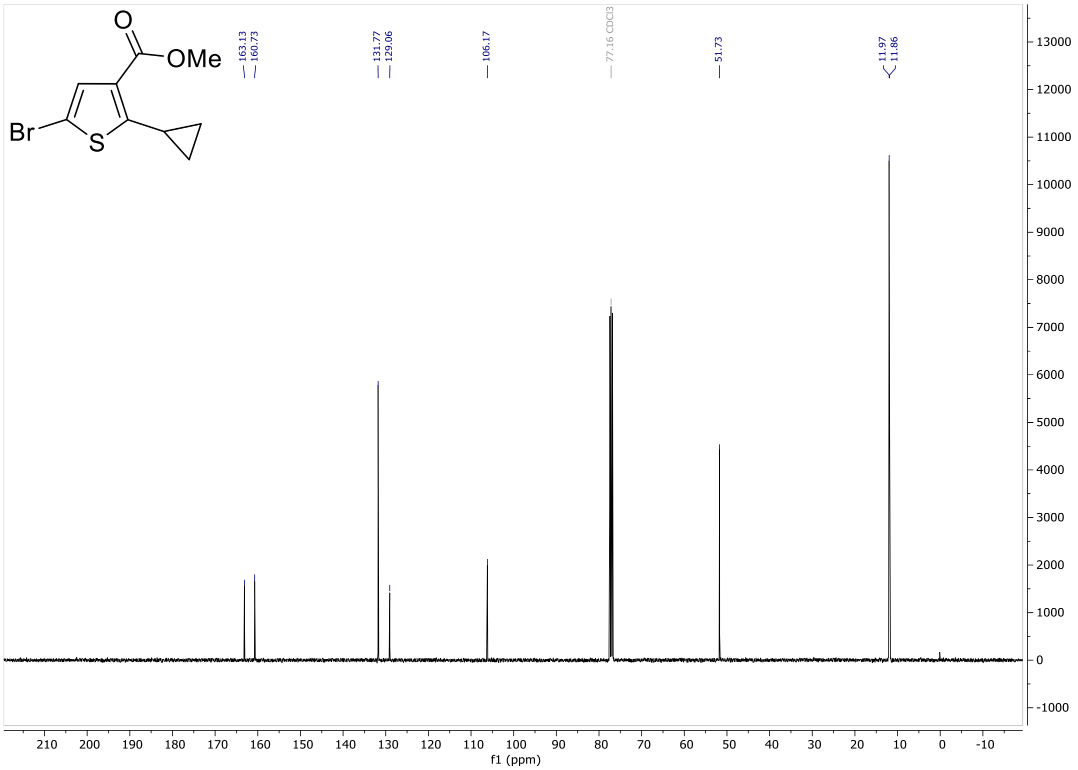

Figure S 52 ^{13}C NMR spectra of compound **7b** in $CDCl_3$ (100 MHz)

1-(5-Bromo-4-cyclopropylthiophen-2-yl)ethan-1-one (8b)

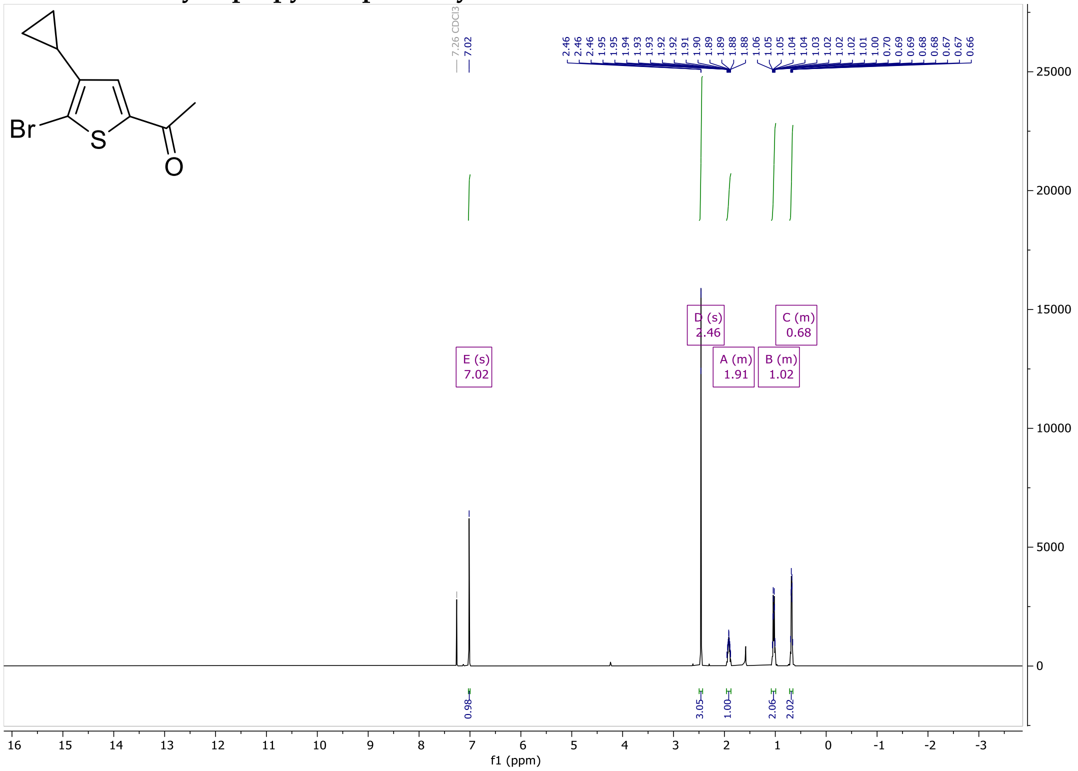

Figure S 53 ^{1}H NMR spectra of compound **8b** in $CDCl_3$ (400 MHz)

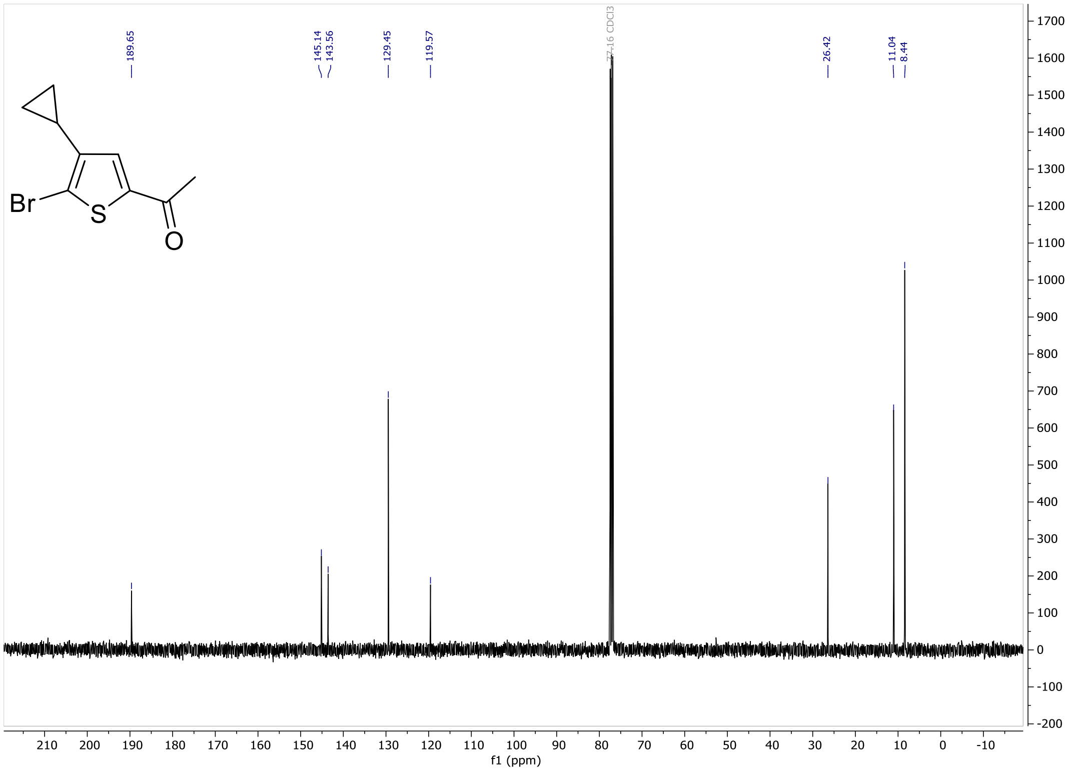

Figure S 54 ^{13}C NMR spectra of compound **8b** in $CDCl_3$ (100 MHz)

5-Bromo-4-cyclopropylthiophene-2-carbaldehyde (9b)

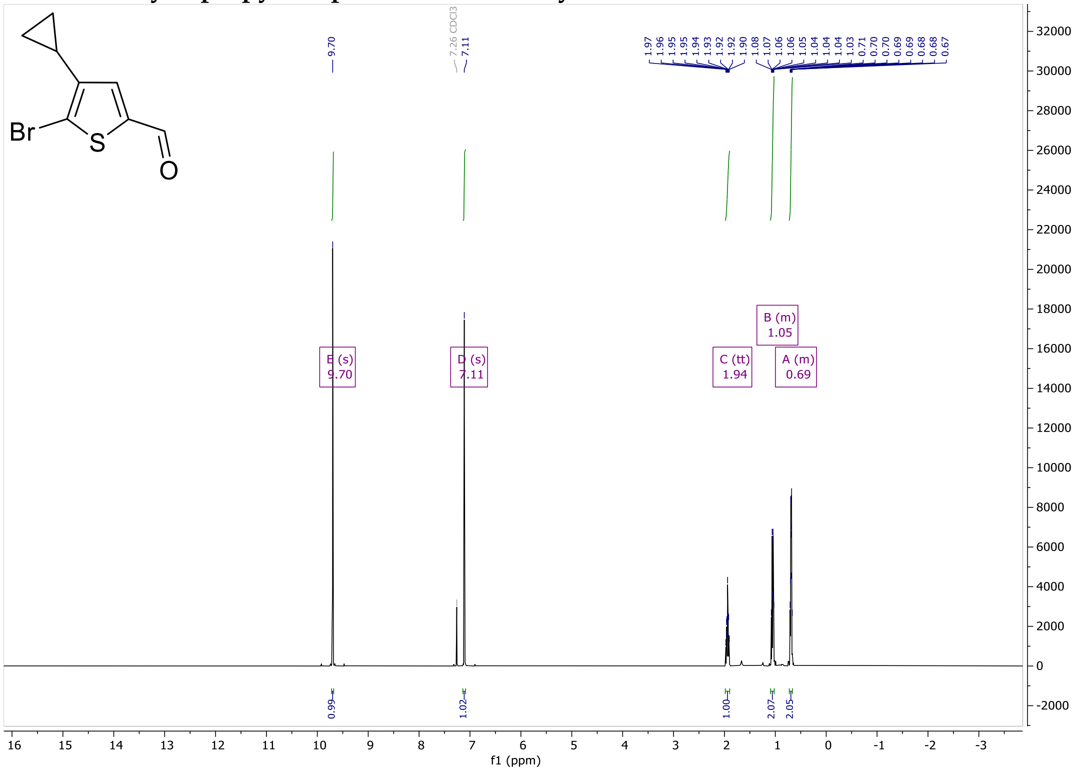

Figure S 55 ^{1}H NMR spectra of compound **9b** in $CDCl_3$ (400 MHz)

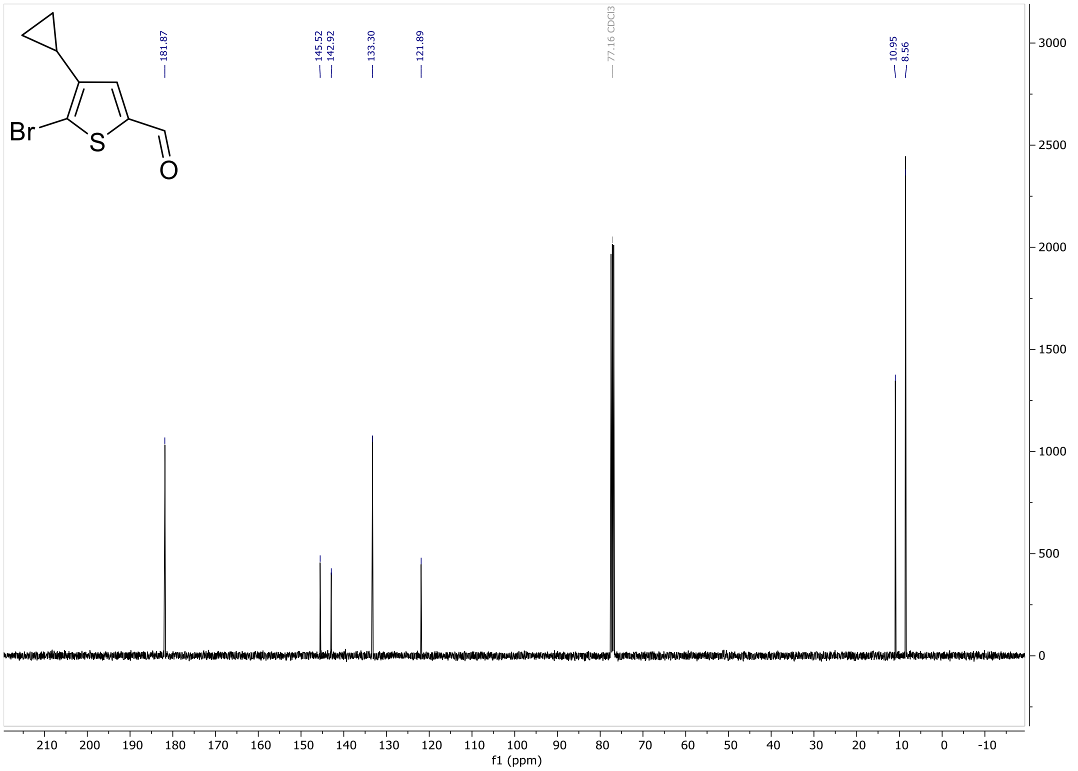

Figure S 56 ^{13}C NMR spectra of compound **9b** in $CDCl_3$ (100 MHz)

5-Bromo-2-cyclopropylthiophene-3-carbonitrile (16b)

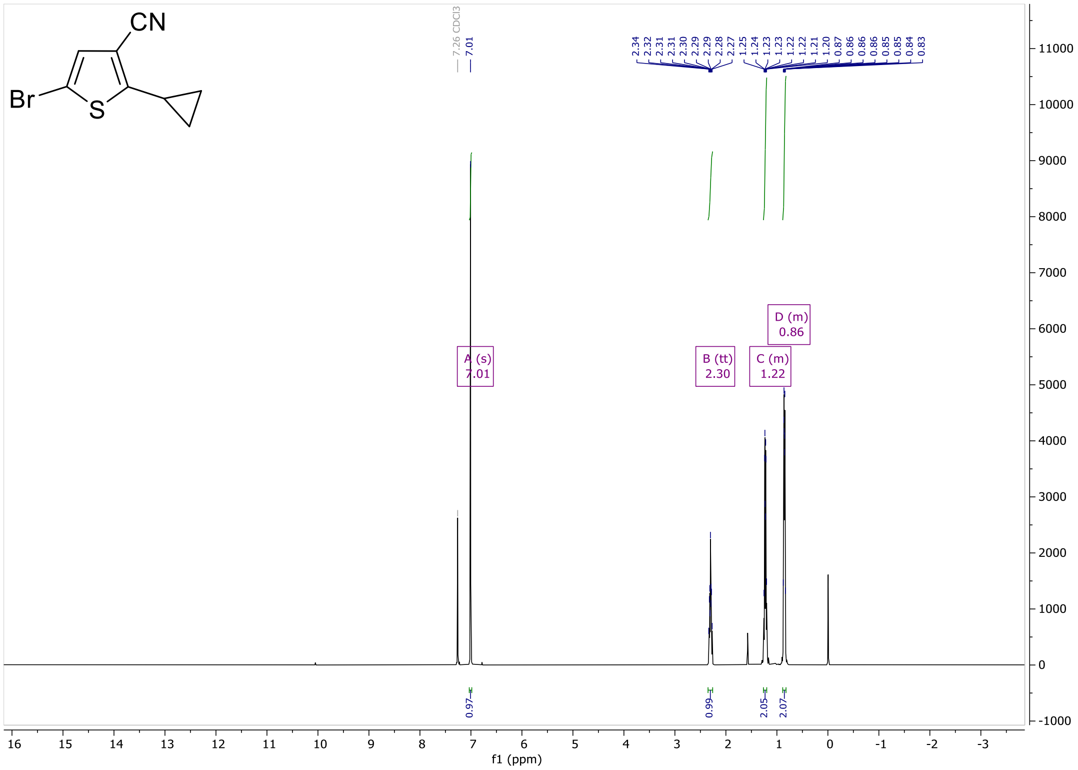

Figure S 57 ^{1}H NMR spectra of compound **16b** in $CDCl_3$ (400 MHz)

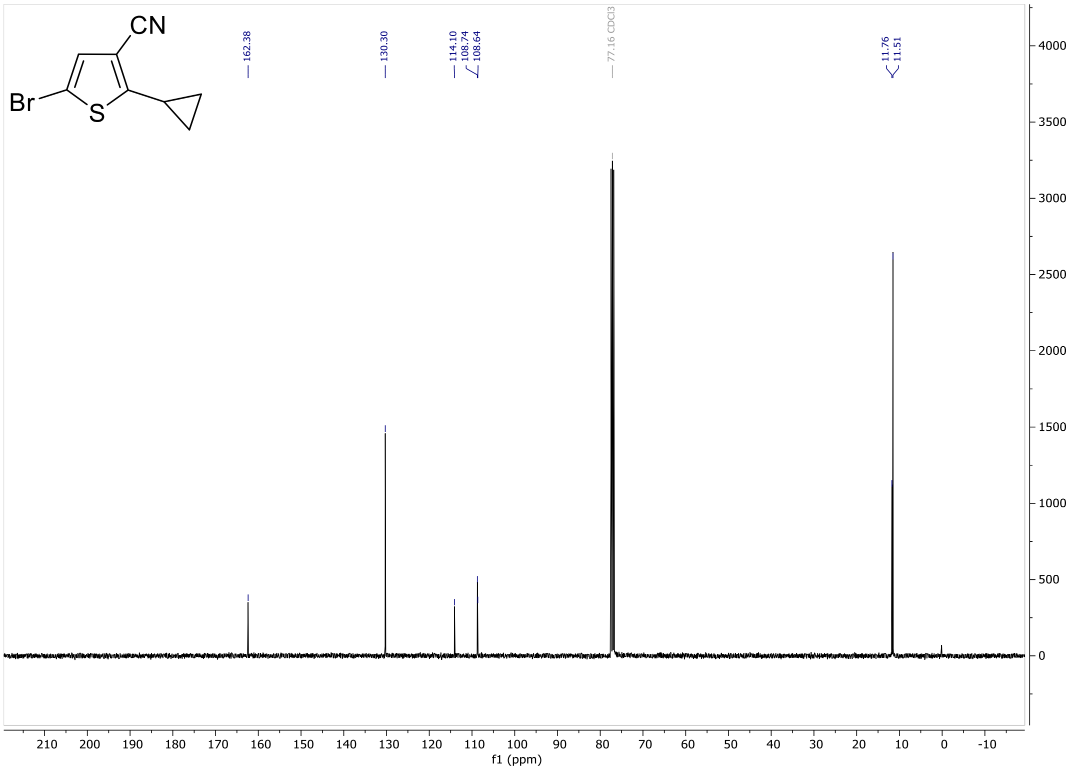

Figure S 58 ^{13}C NMR spectra of compound **16b** in $CDCl_3$ (100 MHz)

2-Bromo-5-cyclopropylthiophene-3-carboxylic acid (20b)

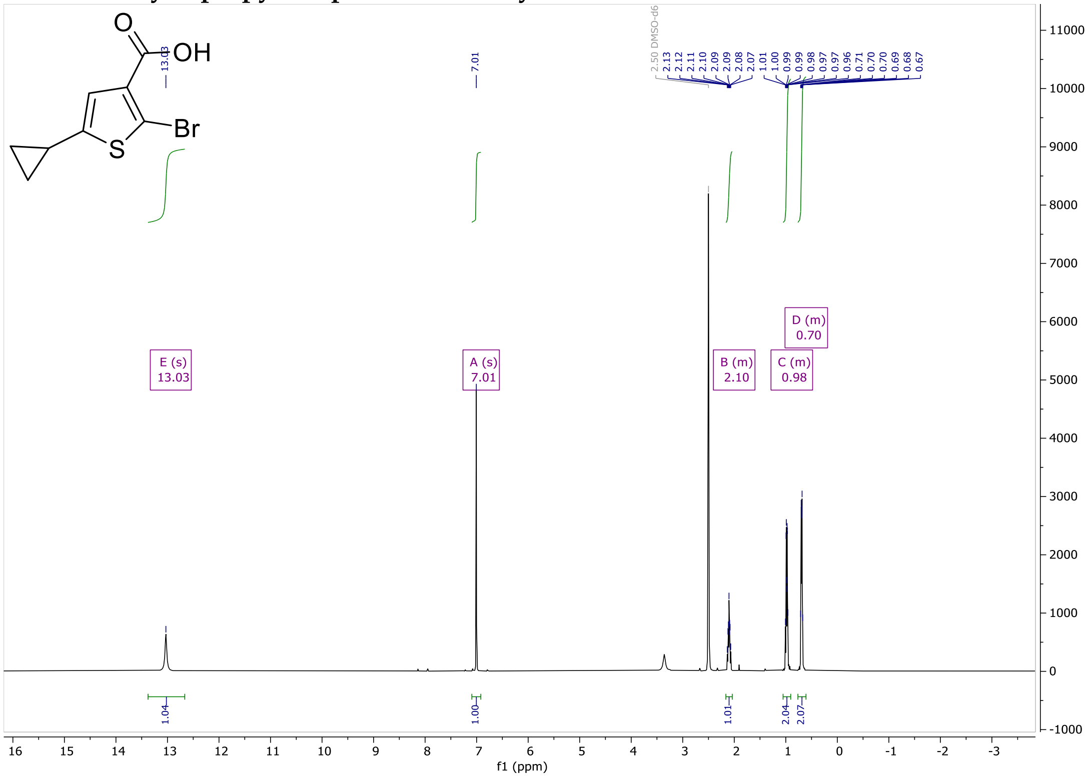

Figure S 59 ^{1}H NMR spectra of compound **20b** in DMSO-d_6 (400 MHz)

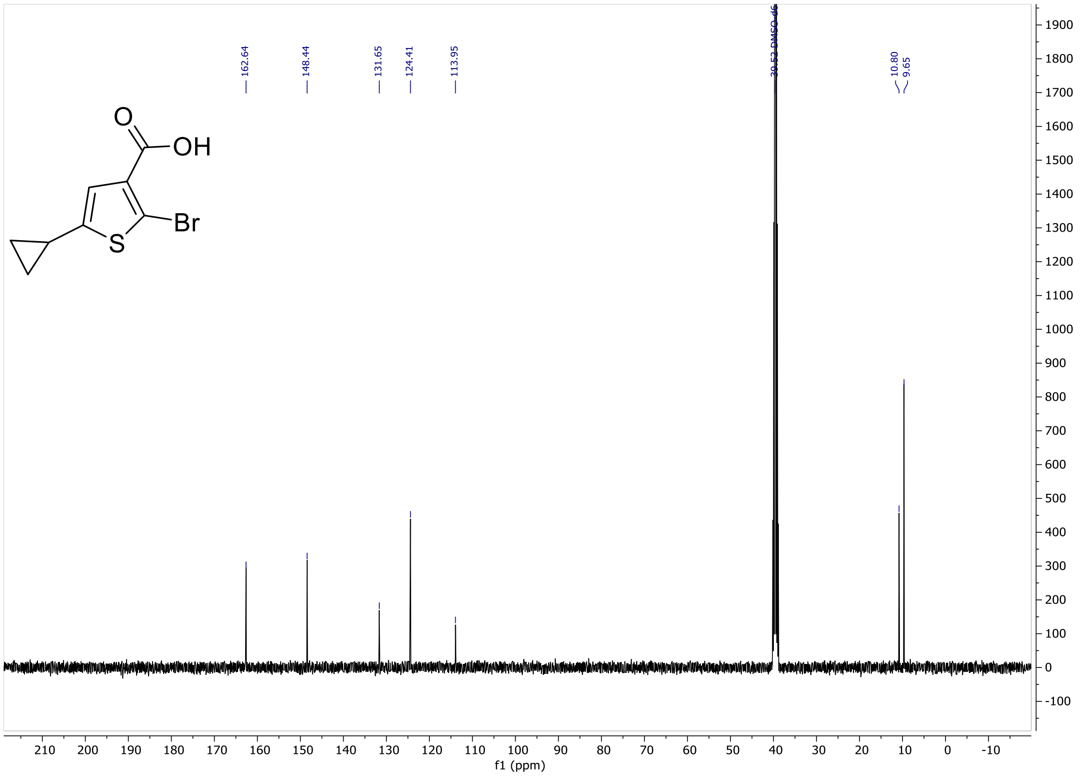

Figure S 60 ^{13}C NMR spectra of compound **20b** in DMSO-d_6 (100 MHz)

5-Bromo-4-cyclopropylthiophene-2-carboxylic acid (22b)

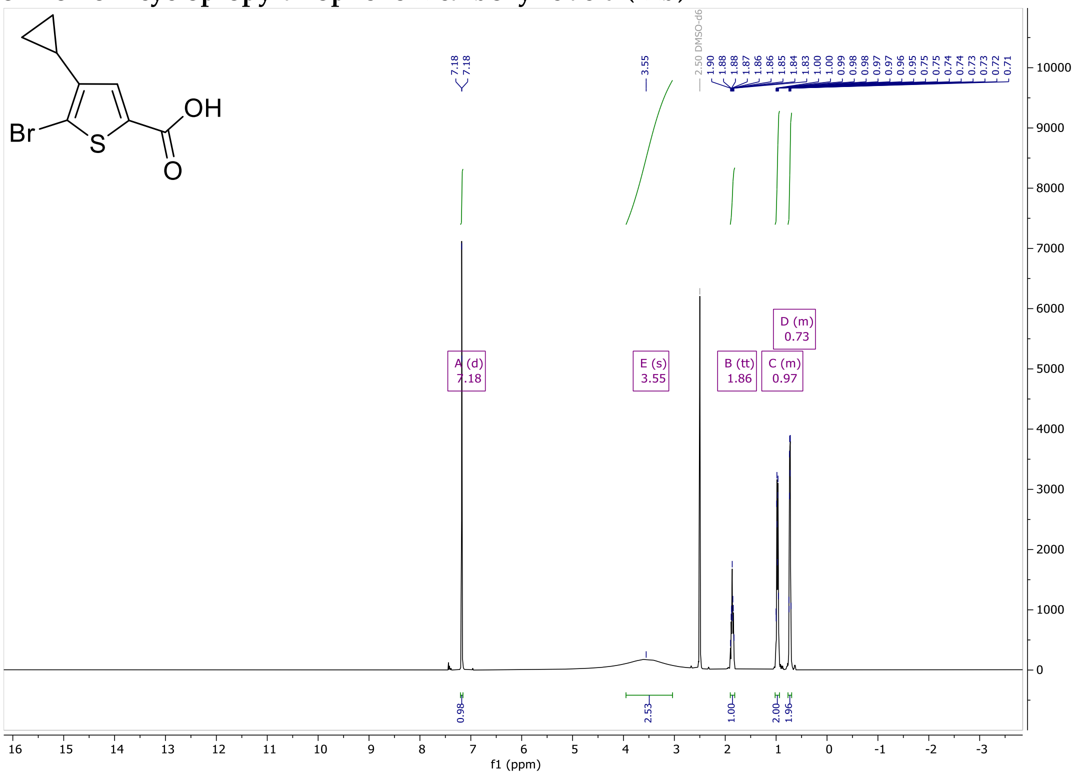

Figure S 61 ^{1}H NMR spectra of compound **22b** in DMSO-d_6 (400 MHz)

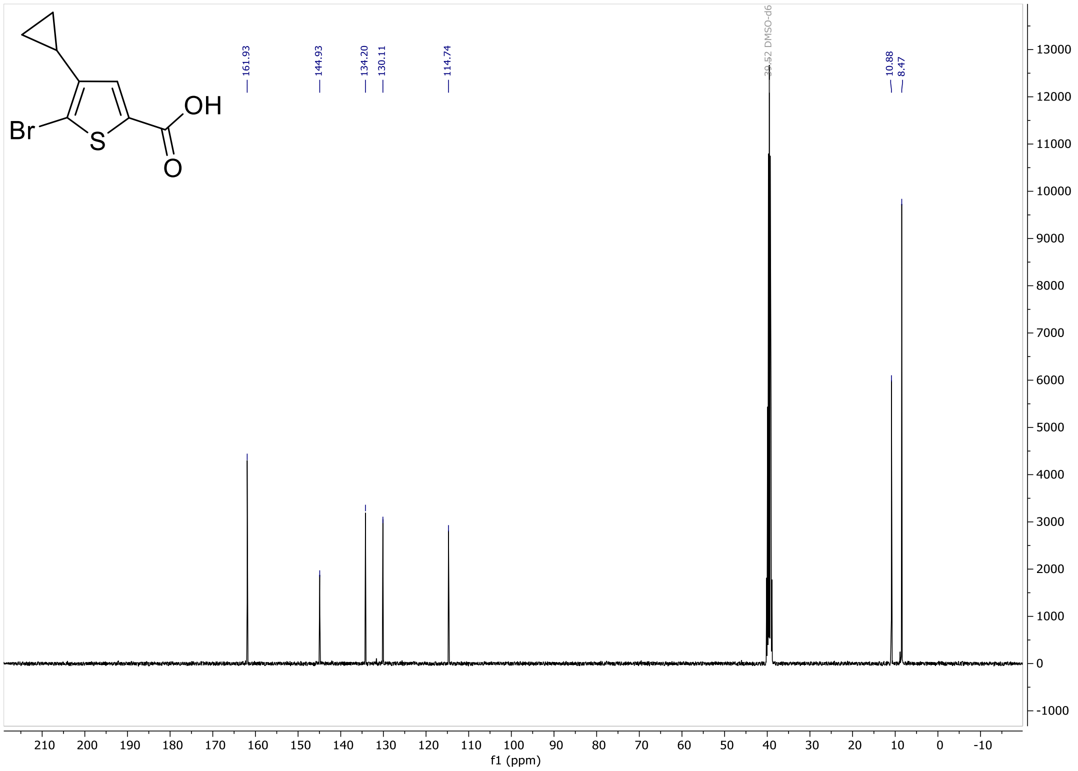

Figure S 62 ^{13}C NMR spectra of compound **22b** in DMSO-d_6 (100 MHz)

5-Cyclopropylthiophene-2-sulfonyl chloride (23a)

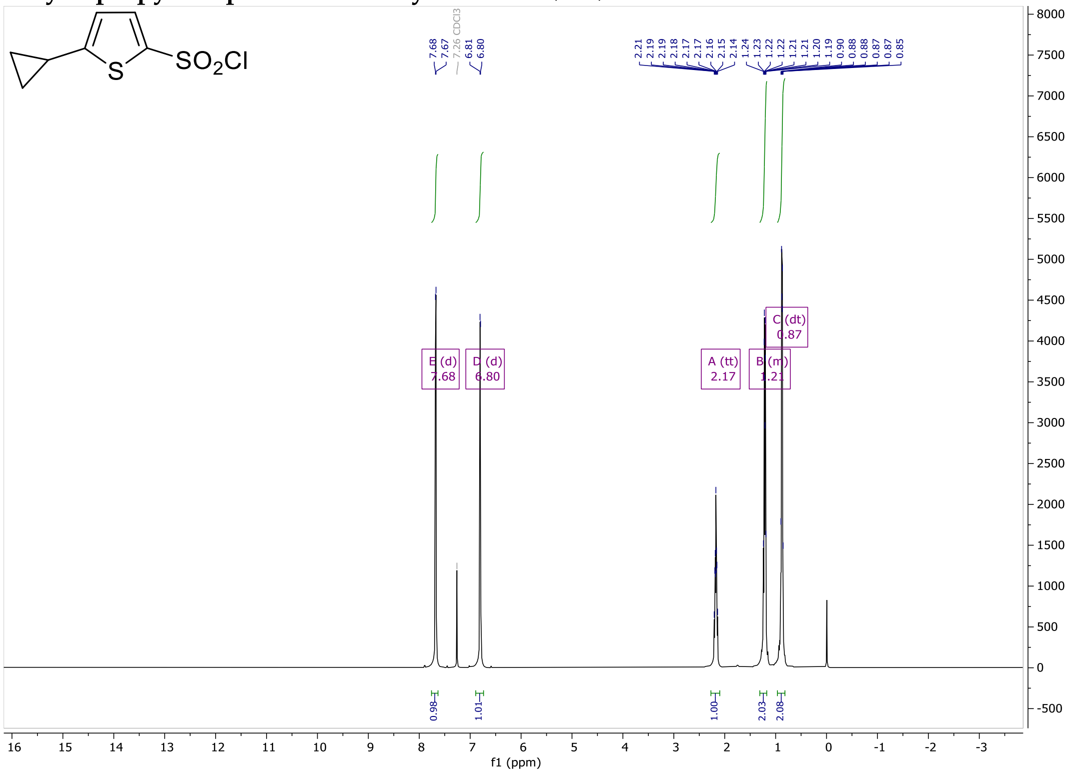

Figure S 63 ^{1}H NMR spectra of compound **23a** in $CDCl_3$ (400 MHz)

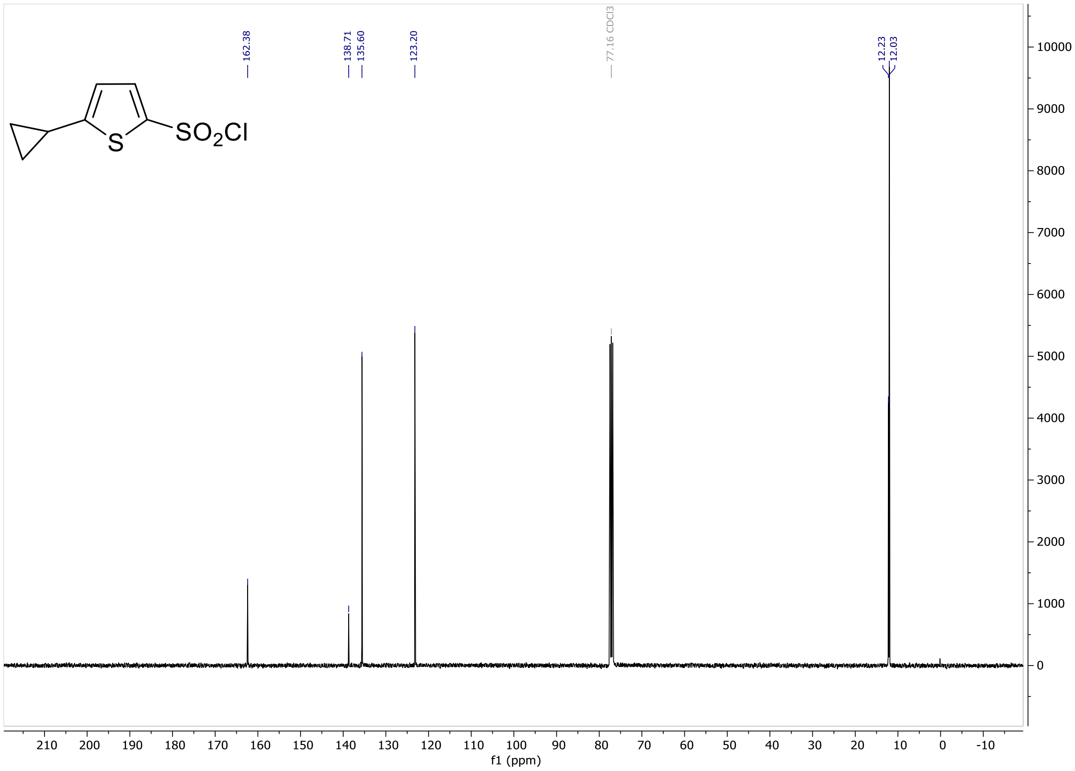

Figure S 64 ^{13}C NMR spectra of compound **23a** in $CDCl_3$ (100 MHz)

4-Cyclopropylthiophene-2-sulfonyl chloride (24a)

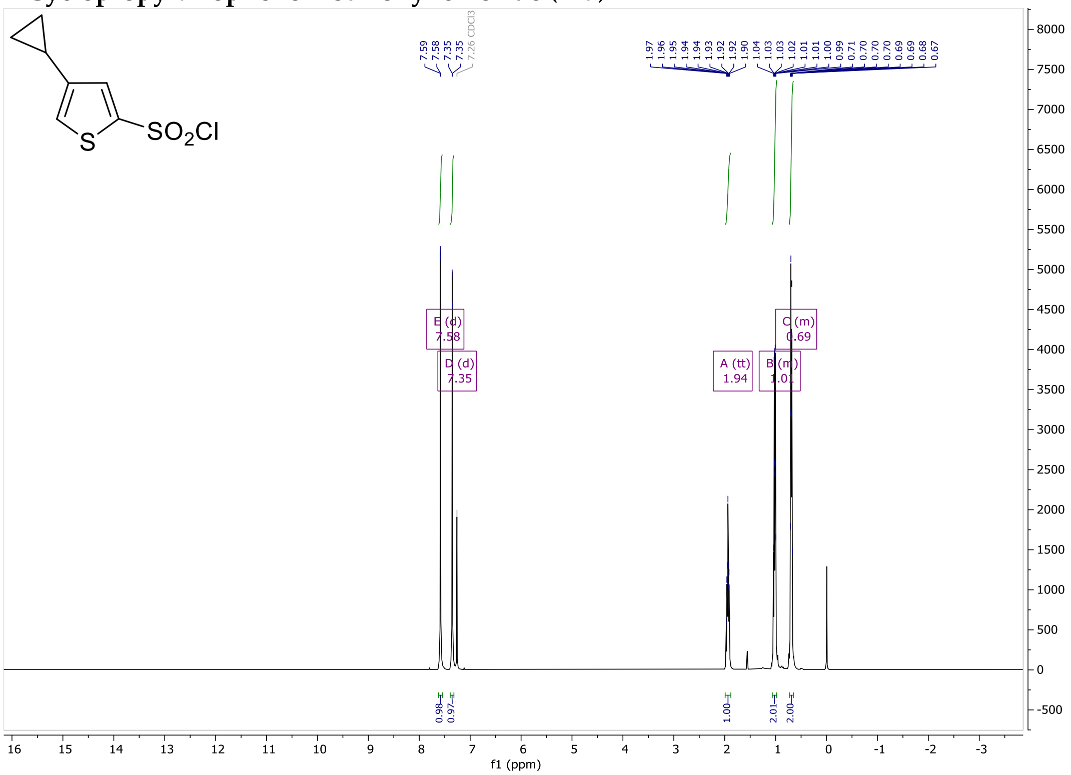

Figure S 65 ^{1}H NMR spectra of compound **24a** in $CDCl_3$ (400 MHz)

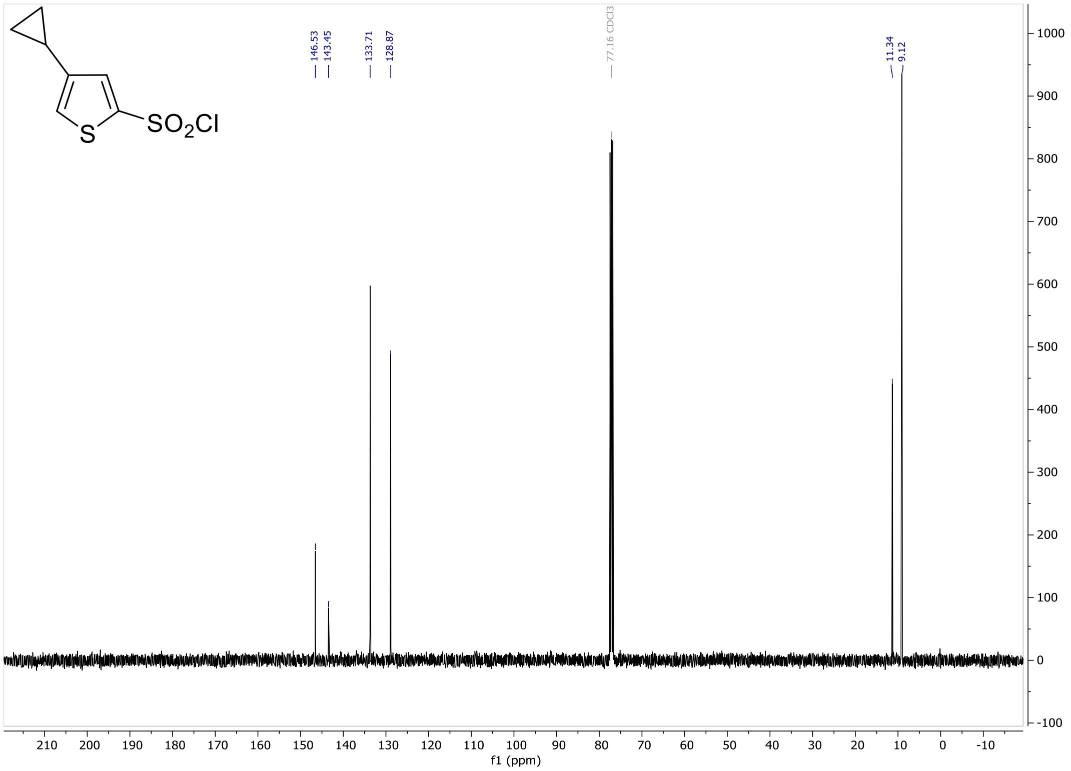

Figure S 66 ^{13}C NMR spectra of compound **24a** in $CDCl_3$ (100 MHz)

3-Cyclopropylthiophene-2-sulfonyl chloride (25a)

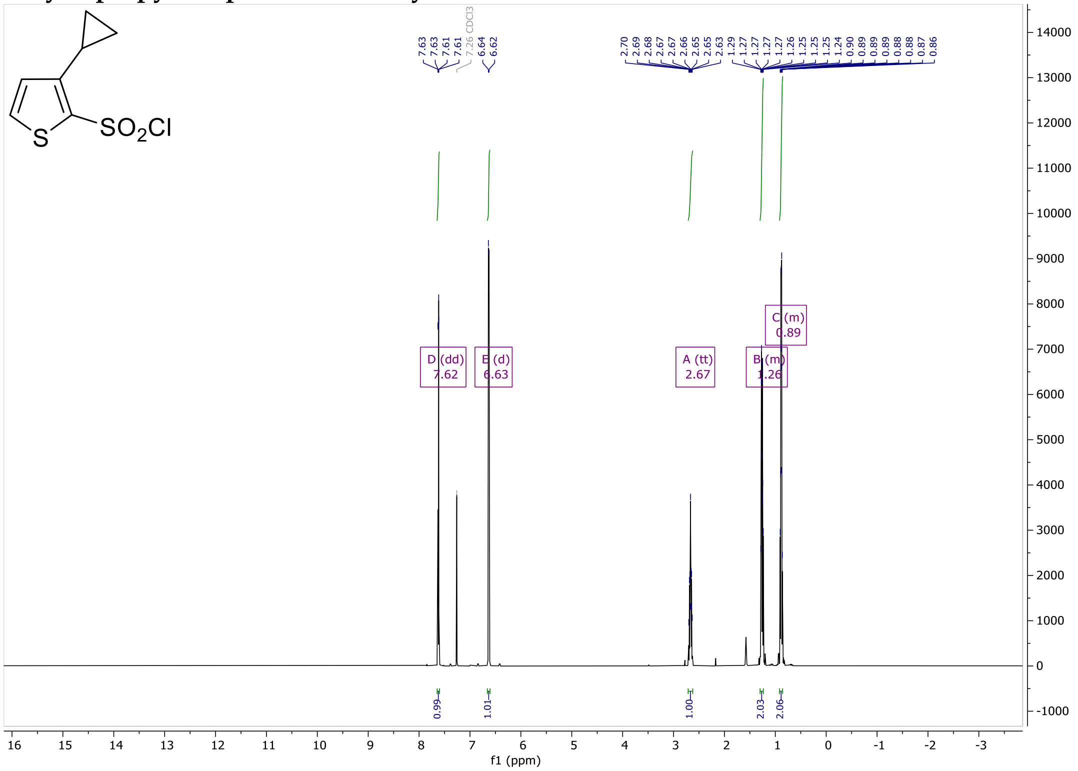

Figure S 67 ^{1}H NMR spectra of compound **25a** in $CDCl_3$ (400 MHz)

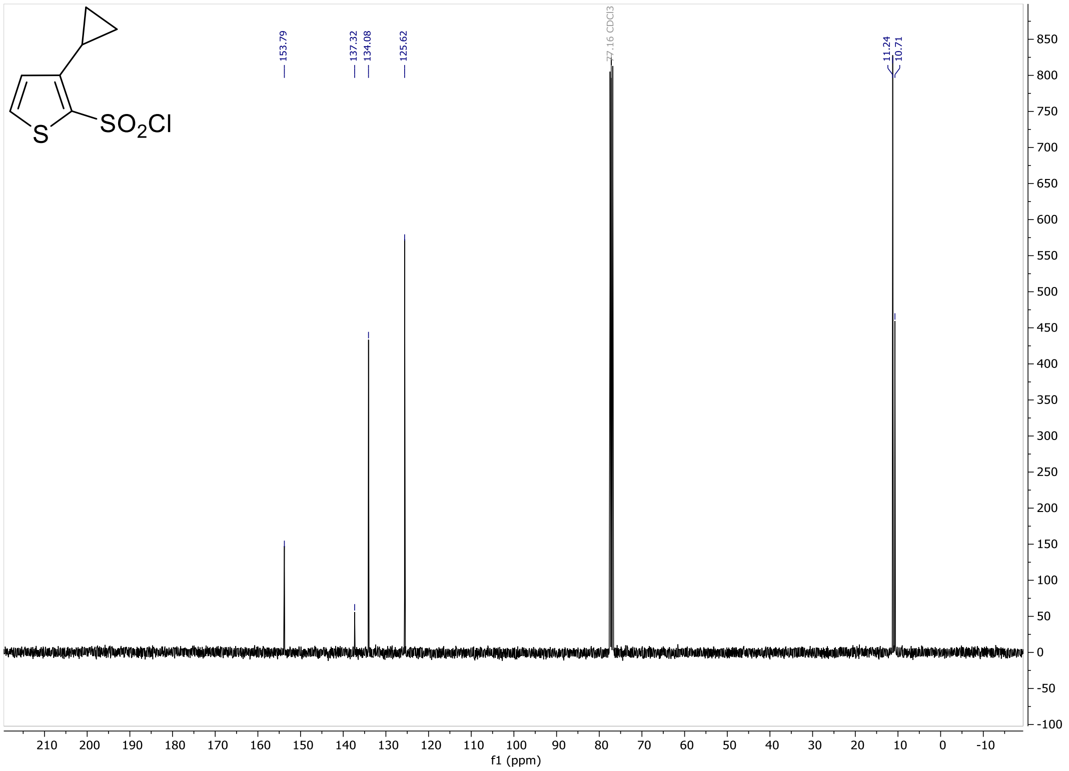

Figure S 68 ^{13}C NMR spectra of compound **25a** in $CDCl_3$ (100 MHz)

3. References

1. Kellogg, R. M.; Buter, J. Cyclopropylthiophenes. Syntheses, Reactions, and Ultraviolet Spectra. *J. Org. Chem*. **1971**, *36* (16), 2236–2244. DOI: 10.1021/jo00815a008.
2. Zhang, H.; Xiao, H.; Jiang, F.; Fang, Y.; Zhu, L.; Li, C. Copper-Catalyzed Ring-Opening 1,3-Aminotrifluoromethylation of Arylcyclopropanes. *Org. Lett.* **2021**, *23* (6), 2268–2272. DOI: 10.1021/acs.orglett.1c00390.
3. Gagnon, A.; Duplessis, M.; Alsabeh, P.; Barabé, F. Palladium-Catalyzed Cross-Coupling Reaction of Tricyclopropylbismuth with Aryl Halides and Triflates. *J. Org. Chem*. **2008**, *73* (9), 3604–3607. DOI: 10.1021/jo702377h.
4. Zhao, X.; Li, R.; Zhou, Y.; Xiao, M.; Ma, C.; Yang, Z.; Zeng, S.; Du, Q.; Yang, C.; Jiang, H.; Hu, Y.; Wang, K.; Ka Pun Mok, C.; Sun, P.; Dong, J.; Cui, W.; Wang, J.; Tu, Y.; Yang, Z.; Hu, W. Discovery of Highly Potent Pinanamine-Based Inhibitors against Amantadine- and Oseltamivir-Resistant Influenza A Viruses. *J. Med. Chem.* **2018**, *61* (12), 5187–5198. DOI: 10.1021/acs.jmedchem.8b00042.
